# Effects of high-heeled shoes on lower extremity biomechanics and balance in females: a systematic review and meta-analysis

**DOI:** 10.1186/s12889-023-15641-8

**Published:** 2023-04-20

**Authors:** Ziwei Zeng, Yue Liu, Xiaoyue Hu, Pan Li, Lin Wang

**Affiliations:** Key Laboratory of Exercise and Health Sciences (Shanghai University of Sport), Ministry of Education, Shanghai, China

**Keywords:** High-heeled shoes, Gait, Kinematics, Kinetics, Meta-analysis

## Abstract

**Background:**

High-heeled shoes (HHS) are widely worn by women in daily life. Limited quantitative studies have been conducted to investigate the biomechanical performance between wearing HHS and wearing flat shoes or barefoot. This study aimed to compare spatiotemporal parameters, kinematics, kinetics and muscle function during walking and balance between wearing HHS and flat shoes or barefoot.

**Methods:**

According to the Preferred Reporting Items for Systematic reviews and Meta-Analyses (PRISMA) statement, PubMed Medline, Cochrane, EMBASE, CINAHL Complete and Web of Science databases were searched from the earliest record to December 2021. A modified quality index was applied to evaluate the risk of bias, and effect sizes with 95% confidence intervals were calculated as the standardized mean differences (SMD). Potential publication bias was evaluated graphically using funnel plot and the robustness of the overall results was assessed using sensitivity analyses.

**Results:**

Eighty-one studies (*n* = 1501 participants) were included in this study. The reduced area of support requires the body to establish a safer and more stable gait pattern by changing gait characteristics when walking in HHS compared with walking in flats shoes or barefoot. Walking in HHS has a slight effect on hip kinematics, with biomechanical changes and adaptations concentrated in the knee and foot–ankle complex. Females wearing HHS performed greater ground reaction forces earlier, accompanied by an anterior shift in plantar pressure compared with those wearing flat shoes/barefoot. Furthermore, large effect sizes indicate that wearing HHS resulted in poor static and dynamic balance.

**Conclusion:**

Spatiotemporal, kinematic, kinetic and balance variables are affected by wearing HHS. The effect of specific heel heights on women’s biomechanics would benefit from further research.

**Supplementary Information:**

The online version contains supplementary material available at 10.1186/s12889-023-15641-8.

## Background

High-heeled shoes (HHS) have been widely worn among women throughout several centuries all over the world. HHS is a type of footwear where the heel is higher than the forepart and usually features a narrow toe section, curved plantar area and a stiff heel cap [1]. Previous evidence showed that 37% to 69% of women wear HHS daily and that 59% of women wear them for 1–8 h per day [2, 3]. However, wearing HHS has been reported to be related to hallux valgus, musculoskeletal pain and first-party injuries, with the incidence of injuries almost doubling from 2002 to 2012 (7.1% to 14.1%) [4–6]. Therefore, identifying the risk factors associated with the incidence of these injuries is essential to protect foot health and implement prevention strategies, such as reduced foot length and increased arch height [7], increased knee varus moment [8] and decreased postural stability [9].

Most studies have confirmed that the effects of wearing HHS are not limited to the foot–ankle complex. The kinematic effects are transmitted up the lower extremity in a chain reaction [1, 10], ultimately leading to changes in spatiotemporal outcomes [11–13], kinematics [11, 14–20], kinetics [12, 21, 22], muscle activity [7, 23–25] and energy expenditure [16, 26]. The available evidence suggests that walking in HHS requires a special neural control that differs from that used in barefooted walking [27]. HHS alter the alignment of the body, thus influencing the body’s centre of gravity (COG) and adversely affecting gait biomechanics and postural stability, impairing static and dynamic balance, and increasing the risk of falls for HHS wearers [9, 28–31].

Various studies have investigated the effect of HHS on gait, posture and relevant injuries on young women [1, 5, 32–34]. A meta-analysis found that walking in HHS increased knee flexion moment, flexion angle and varus moment during the early stance phase [35]. However, to date, meta-analyses of the effects of HHS on gait spatiotemporal outcomes, joint kinematics, kinetics, muscle activity and balance in female have been lacking. Changes in women’s gait parameters when wearing HHS and the associated neuromuscular and biomechanical adaptations can provide accurate and effective recommendations for future efforts to eliminate the negative effects of high heels [1]. Therefore, the aim of this review is to collect the available evidence to investigate the effects of wearing HHS on lower limbs biomechanics and balance in females and provide guidance for further research in this area.

## Methods

The systematic review was conducted in accordance with guidelines provided by the Preferred Reporting of Systematic Reviews and Meta-Analysis (PRISMA) statement (PROSPERO registration number CRD42021291135) [36].

### Search strategy

PubMed Medline, Cochrane, EMBASE, CINAHL Complete and Web of Science electronic databases were searched from inception until December 2021. Full search terms and strategies are available in Additional file 1. No restrictions were set on literature type or publication status.

### Eligibility criteria

Journal articles that evaluated the effects of HHS on lower extremity biomechanics and balance in healthy women were included, covering indicators such as gait spatiotemporal, joint kinematics, kinetics and muscle activity variables during horizontal walking, as well as static and dynamic balance variables. The specific inclusion and exclusion criteria can be found in Additional file 2.

### Study selection

All the studies searched were imported into EndNote X9 (Clarivate Analytics), and the duplicate articles were removed by a reviewer (ZZ). Each title and abstract were screened for eligibility inclusion by two independent reviewers (YL and XH). Then, the reference lists of all included articles were screened manually to identify any relevant studies that might have been missed by electronic searches. Any disagreements between the two reviewers were resolved with a consensus meeting, if necessary, and the decision was made by a third reviewer (LW).

### Data extraction

Study characteristics were extracted by one reviewer (ZZ) and verified by a second (YL) using a standardized template, including (1) article details (authors name and year of publication), (2) participant characteristics (sample size, age, body height, body mass and HHS wearing experience), (3) HHS used (heel height), (4) experimental characteristics (comparisons and walking speed) and (5) biomechanical variables investigated (spatiotemporal, kinematics, kinetics, muscle function and balance). Any discrepancies were discussed by all reviewers.

### Assessment of risk of bias

A modified Downs and Black’s Quality Index (QI) tool with high reliability and validity was used by two reviewers (PL and XH) to assess the methodological quality [37]. According to the purpose of this review, the QI contains following four categories: study reporting (items 1–4, 6–7 and 10), external validity (items 11 and 12), internal validity (items 16, 18, 20, 22 and 23) and power (item 27) [38, 39] (see Additional file 3). Articles with scores of 6 and below (≤ 40%), 6–12 (40%–80%) and 12 and above (≥ 80%) were considered low, moderate and high quality, respectively [40]. Discrepancies were discussed and resolved through a consensus meeting. If a consensus was not achieved, then a third reviewer (LW) served as the tiebreaker. The mean kappa agreement between the reviewers was 0.96.

### Data analysis

Means and standard deviations for included variables were extracted to calculate the between-group standardized mean difference (SMD) and 95% confidence interval (95% CI) for study comparisons [38]. Meta-analysis for each outcome was performed using Review Manager (version 5.3, The Cochrane Collaboration, Copenhagen, Denmark) with at least two studies with available data on HHS (≥ 3 cm) compared with flat shoes or barefoot [41]. When a study was conducted on females with and without experience wearing HHS, they were included in the meta-analysis as two groups of data. To include as much data as possible, when studies were reported for both limbs, they were treated as independent data. Heterogeneity was evaluated using the I^2^ and Q statistics, where I^2^ > 50% or a significant Q statistic indicated statistical heterogeneity, when I^2^ values were > 50%, the random-effect model was applied for data analyses, otherwise the fixed-effect model was used [42]. The effect size was categorized as trivial (≤ 0.20), small (0.20–0.50), moderate (0.50–0.80) or large (≥ 0.80) [43]. Subgroup analysis was conducted based on different biomechanical variables. Sensitivity analyses were performed to investigate the robust of the pooled results and funnel plots were applied to evaluate the potential publication bias [44]. Statistical significance was set at *p* < 0.05.

## Results

### Search results

**Fig. 1:**
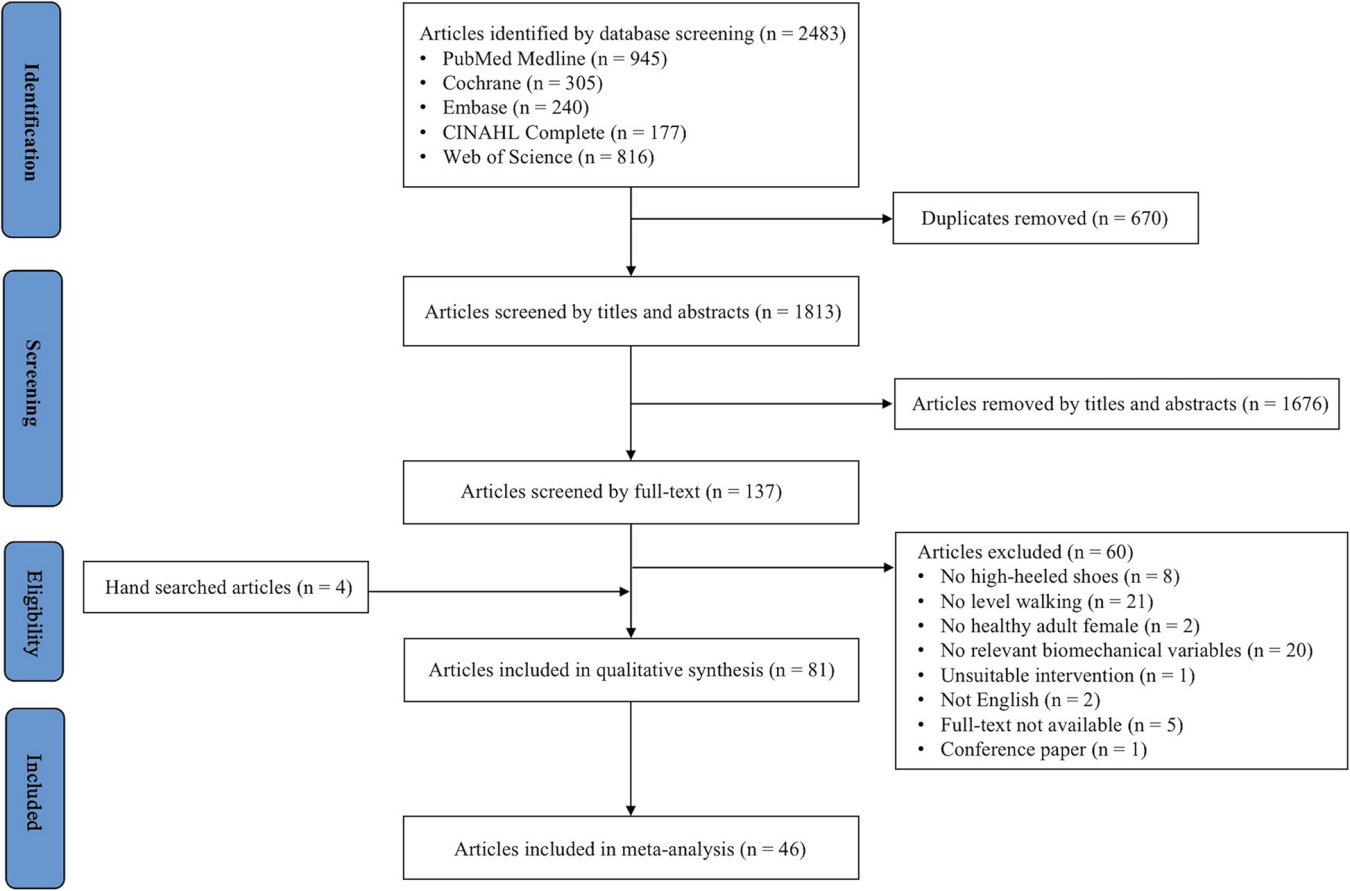
Flowchart of the systematic review selection process

The initial electronic database searches identified 2483 records (Fig. 1). After duplicates and screening of titles and abstracts were removed, 137 studies remained. An additional four records were included through cross-referencing. After full-text screening, 81 articles met the eligibility criteria and were included in this review.

### Quality assessment of included studies

**Fig. 2:**
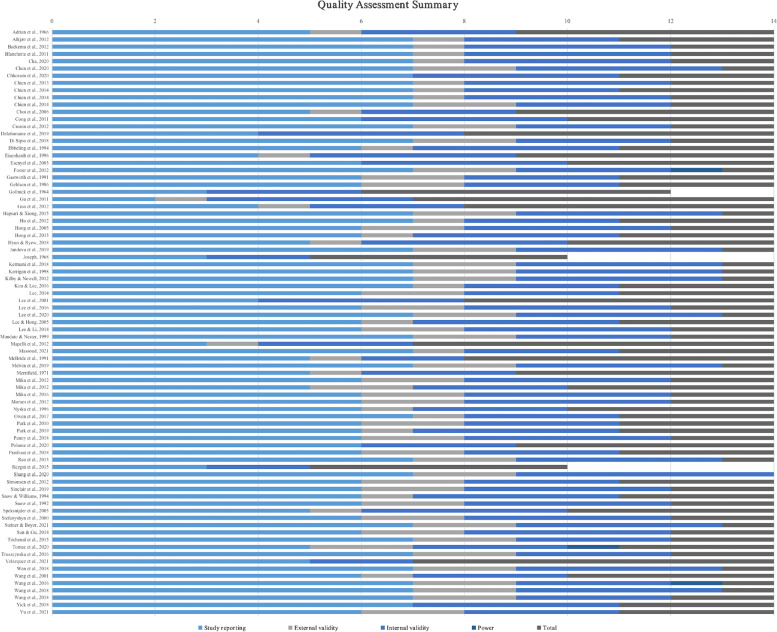
Methodological quality for the included studies

Total scores from the QI and a breakdown for each category for all included articles are shown in Fig. 2. The number of studies graded as low, moderate and high quality was 3, 63 and 15, respectively. The QI scores of assessed studies ranged from 5 to 14, with a mean (SD) = 10.95 (1.91) points.

### Study characteristics

The sample sizes ranged from 3 to 71, with 15 being the most common (*n* = 14), and the mean (SD) was 18 (12). The mean (SD) age of all participants was 25.08 (4.08) years and 24.74 (4.19) years for studies included in the review and meta-analysis, respectively. Nine articles compared women with and without HHS wearing experience [9, 16, 18, 31, 45–49]. In addition to several studies that used the subjects’ own HHS with different heel heights [17, 22, 50–56], the heel heights involved in this study ranged from 3 to 18 cm, with 9 cm (*n* = 11), 10 cm (*n* = 11), 6 cm (*n* = 10) and 7 cm (*n* = 10) being most commonly used. The walking speed involved a wide range from 0.56 m/s to 1.61 m/s, with 1.3 m/s being the most frequent (*n* = 7) and the mean (SD) was 1.17 (0.21) m/s (see Additional file 4).

### Effect of HHS on lower extremity biomechanics and balance

#### Spatiotemporal characteristics

**Fig. 3:**
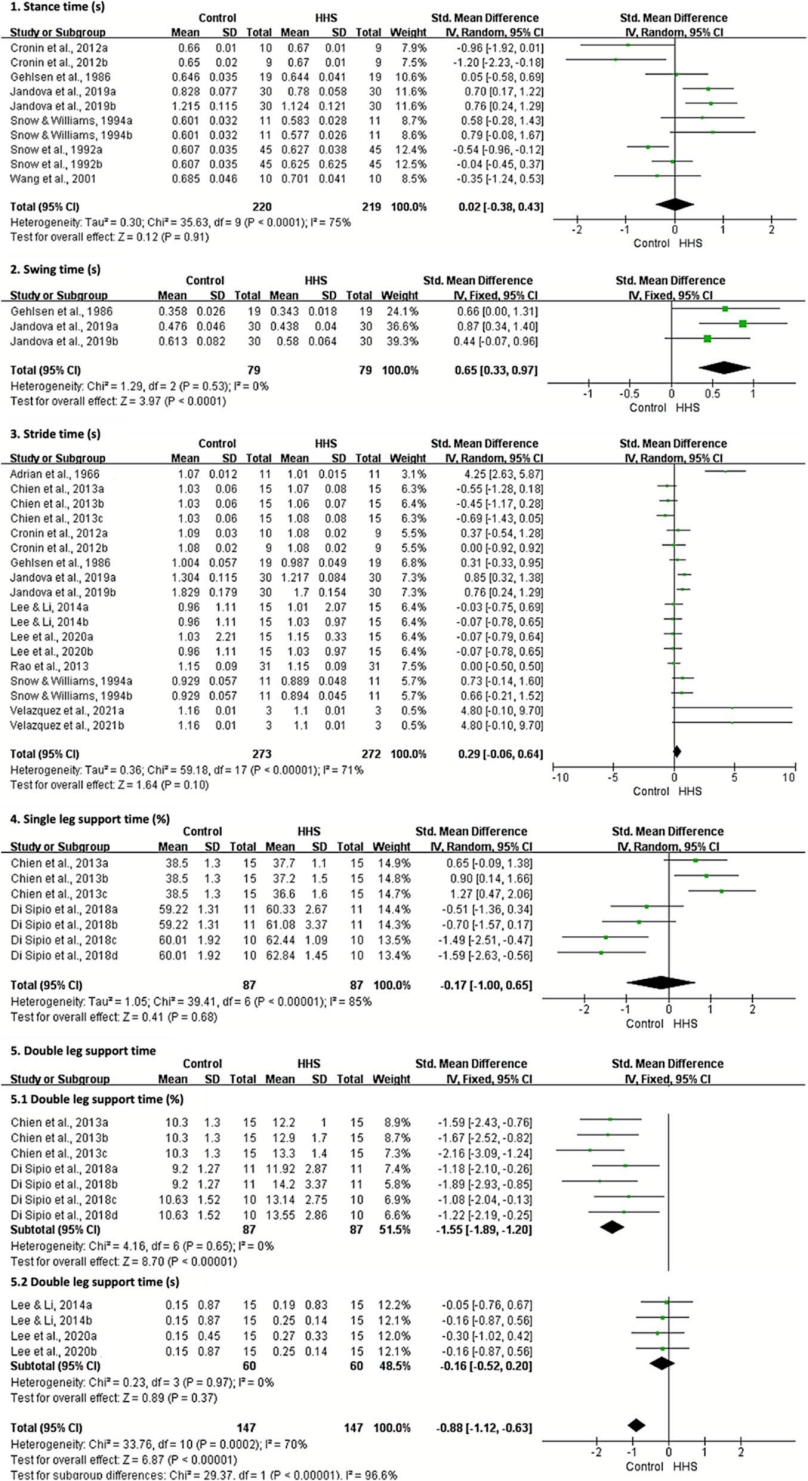
Meta-analysis of temporal characteristics during walking in high-heeled shoes compared with flat shoes or barefoot. IV inverse variance, CI confidence interval, HHS high-heeled shoes

Meta-analysis showed no statistically significant effects for wearing HHS on stance time, stride time, single leg support time and step frequency during level walking (*p* ≥ 0.10). Large effects indicated that walking in HHS resulted in shorter swing time (SMD = 0.65; 95% CI 0.33, 0.97; *p* < 0.001), step length (SMD = 1.49 95% CI 0.88, 2.11; *p* < 0.001), stride length (SMD = 0.78; 95% CI 0.44, 1.11; *p* < 0.001), step width (SMD = 0.85; 95% CI 0.53, 1.16; p < 0.001) and walking velocity (SMD = 0.75; 95% CI 0.48, 1.03; *p* < 0.001). The subgroup analysis showed that walking with HHS provoked a longer double leg support time (percentage: SMD =  − 1.55; 95% CI − 1.89, − 1.20; *p* < 0.001; total: SMD =  − 0.88; 95% CI − 1.12, − 0.63; *p* < 0.001) (Fig. 3 and Additional file 5). No clear asymmetries were identified in the funnel plots for the related parameters, except for step length and step width (see Additional file 6). For all relevant parameters, the exclusion of individual studies did not trigger a significant change in the overall results in the sensitivity analyses.

### Kinematics

#### Hip

Meta-analysis showed a statistically significant increase in range of motion (ROM) (SMD =  − 1.59; 95% CI − 2.26, − 0.91; *p* < 0.001) during gait cycle when walking with HHS. HHS did not have a statistically significant effect on peak flexion and extension during stance phase and gait cycle and flexion at foot strike (*p* ≥ 0.05) (see Additional file 5). For all relevant parameters, no clear asymmetries were identified in the funnel plots (see Additional file 6). In the sensitivity analyses, only the peak flexion angle during gait cycle showed a significant change in the overall result after removing the article written by Snow and Williams [15].

### Knee

Meta-analysis showed a statistically significant increase in flexion at foot strike (SMD =  − 0.42; 95% CI − 0.63, − 0.22; *p* < 0.001) and flexion during mid-stance (SMD =  − 0.71; 95% CI − 0.97, − 0.45; *p* < 0.001), but a decrease in peak flexion (SMD = 0.61; 95% CI 0.05, 1.16; p 0.03) and ROM (SMD = 1.12; 95% CI 0.39, 1.85; p 0.003) during gait cycle and flexion at toe-off (SMD = 0.84; 95% CI 0.53, 1.15; *p* < 0.001) when walking with HHS. HHS did not have a statistically significant effect on peak flexion during stance phase and peak extension during stance phase and gait cycle (*p* ≥ 0.10) (see Additional file 5). For all relevant parameters, no clear asymmetries were identified in the funnel plots (see Additional file 6). In the sensitivity analyses, only the peak flexion angle during gait cycle showed a significant change in the overall result after removing the articles written by Snow and Williams [15] and Di Sipio et al. [57].

### Foot–ankle complex

Meta-analysis showed a statistically significant greater plantarflexion in peak plantarflexion during stance phase (SMD = 2.37; 95% CI 1.41, 3.33; *p* < 0.001) and gait cycle (SMD = 2.17; 95% CI 1.15, 3.18; *p* < 0.001), peak dorsiflexion during stance phase (SMD = 2.99; 95% CI 1.99, 4.00; p < 0.001) and gait cycle (SMD = 1.56; 95% CI 0.62, 2.50; p 0.001) and plantarflexion at foot strike (SMD = 2.64; 95% CI 1.47, 3.80; *p* < 0.001) and toe-off (SMD = 1.08; 95% CI 0.65, 1.51; *p* < 0.001) when walking with HHS. Conversely, ROM decreased statistically significantly during gait cycle (SMD = 1.71; 95% CI 1.06, 2.36; *p* < 0.001). HHS did not have a statistically significant effect on the rearfoot angle at foot strike and peak inversion during stance phase (p ≥ 0.10) (see Additional file 5). For all relevant parameters, no clear asymmetries were identified in the funnel plots (see Additional file 6). In the sensitivity analyses, only the rearfoot angle at foot strike showed a significant change in the overall result after removing the article written by Ebbeling et al. [16].

### Kinetics

#### Hip

Meta-analysis showed no statistically significant effect of HHS on peak flexion moment and peak extension moment during gait cycle (*p* ≥ 0.08) (see Additional file 5). For all relevant parameters, no clear asymmetries were identified in the funnel plots (see Additional file 6). In the sensitivity analyses, only the peak extension moment during gait cycle showed a significant change in the overall result after removing the article written by Esenyel et al. [13].

### Knee

Meta-analysis showed a statistically significant increase in peak flexion (SMD =  − 0.59; 95% CI − 0.93, − 0.26; *p* < 0.001) and extension (SMD =  − 0.40; 95% CI − 0.76, − 0.04; p 0.03) moments during gait cycle when walking with HHS. HHS did not have a statistically significant effect on peak adduction and abduction moments during gait cycle (*p* ≥ 0.32) (see Additional file 5). For all relevant parameters, no clear asymmetries were identified in the funnel plots (see Additional file 6). In the sensitivity analyses, only the peak extension moment during gait cycle showed a significant change in the overall result after removing the articles written by Esenyel et al. [13] and Lee et al. [58].

### Foot–ankle complex

Meta-analysis showed a statistically significant decrease in peak plantarflexion moment during gait cycle (SMD = 0.90; 95% CI 0.38, 1.42; *p* < 0.001) when walking with HHS. HHS did not have a statistically significant effect on peak dorsiflexion moment during gait cycle (p = 0.37) (see Additional file 5). For all relevant parameters, no clear asymmetries were identified in the funnel plots and the exclusion of individual studies did not trigger a significant change in the overall results in the sensitivity analyses (see Additional file 6).

### Ground reaction forces

Meta-analysis showed a statistically significant increase in the first (SMD =  − 0.58; 95% CI − 0.88, − 0.28; *p* < 0.001) and the second (SMD =  − 1.11; 95% CI − 1.63, − 0.59; *p* < 0.001) peak vertical ground reaction force (GRF) and % time to the second peak vertical GRF (SMD =  − 0.78; 95% CI − 1.14, − 0.43; *p* < 0.001) when walking with HHS. HHS did not have a statistically significant effect on % time to the first peak vertical GRF (*p* = 0.21) (see Additional file 5). For all relevant parameters, no clear asymmetries were identified in the funnel plots and the exclusion of individual studies did not trigger a significant change in the overall results in the sensitivity analyses (see Additional file 6).

### Plantar pressure

Meta-analysis showed a statistically significant increase in peak pressure under forefoot (hallux: SMD =  − 1.26; 95% CI − 1.54, − 0.98; *p* < 0.001; other toes: SMD =  − 1.52; 95% CI − 2.08, − 0.95; *p* < 0.001; the first metatarsals: SMD =  − 1.45; 95% CI − 1.90, − 1.00; *p* < 0.001; the second and third metatarsals: SMD =  − 1.18; 95% CI − 1.54, − 0.82; *p* < 0.001; the fourth and fifth metatarsals: SMD =  − 0.63; 95% CI − 1.09, − 0.17; *p* 0.007), but a decrease in peak pressure under the midfoot (total: SMD = 1.62; 95% CI 1.01, 2.23; *p* < 0.001; lateral: SMD = 1.67; 95% CI 1.08, 2.25; *p* < 0.001; medial: SMD = 0.61; 95% CI 0.18, 1.04; p 0.006) and heel (lateral: SMD = 0.81; 95% CI 0.39, 1.24; *p* < 0.001; medial: SMD = 1.42; 95% CI 0.66, 2.19; *p* < 0.001) when walking with HHS. HHS did not have a statistically significant effect on peak pressure under total heel (*p* = 0.07) (see Additional file 5). For all relevant parameters, no clear asymmetries were identified in the funnel plots (see Additional file 6). In the sensitivity analyses, the peak pressure under the medial midfoot showed a significant change in the overall result after removing the article written by Guo et al. [59] and the peak pressure under the total heel showed a significant change in the overall result after removing the article written by Penny et al. [60].

Meta-analysis showed a statistically significant increase in impact force (SMD =  − 1.25; 95% CI − 2.02, − 0.49; p 0.001), maximum force in the hallux (SMD =  − 1.71; 95% CI − 2.30, − 1.12; *p* < 0.001) and other toes (SMD =  − 1.84; 95% CI − 2.48, − 1.20; *p* < 0.001) when walking with HHS. Conversely, a statistically significant decrease in maximum force was observed in the heel (lateral: SMD = 0.70; 95% CI 0.45, 0.94; *p* < 0.001; medial: SMD = 1.11; 95% CI 0.40, 1.83; p 0.002) (see Additional file 5). For all relevant parameters, no clear asymmetries were identified in the funnel plots and the exclusion of individual studies did not trigger a significant change in the overall results in the sensitivity analyses (see Additional file 6).

Meta-analysis showed a statistically significant increase contact area in the hallux (SMD =  − 2.09; 95% CI − 2.70, − 1.47; *p* < 0.001) and other toes (SMD =  − 1.97; 95% CI − 2.64, − 1.30; *p* < 0.001), but a decrease in contact area in the heel (lateral: SMD = 0.26; 95% CI 0.02, 0.50; p 0.03; total: SMD = 0.28; 95% CI 0.10, 0.46; p 0.002) when walking with HHS. HHS did not have a statistically significant effect on contact area in the medial heel (*p* = 0.08) (see Additional file 5). For all relevant parameters, no clear asymmetries were identified in the funnel plots (see Additional file 6). In the sensitivity analyses, only the contact area in the lateral heel showed a significant change in the overall result after removing the article written by Shang et al. [61].

### Lower extremity muscle function

Nineteen studies investigated the effects of HHS on muscle function. However, the differences in the studied muscles and indexes were not sufficient for the meta-analysis.

## Balance

Meta-analysis showed a statistically significant increase in the time spent during the timed up and go test (SMD =  − 0.60; 95% CI − 0.81, − 0.40; *p* < 0.001) and mean anterior–posterior COP sway during standing (SMD =  − 0.88; 95% CI − 1.32, − 0.44; *p* < 0.001) when wearing HHS. Conversely, a statistically significant decrease occurred in functional reach test score (SMD = 0.55; 95% CI 0.23, 0.87; *p* < 0.001), single leg stance time (SMD = 2.52; 95% CI 0.95, 4.09; p 0.002) and limits of stability test scores (COG movement velocity: SMD = 0.61; 95% CI 0.24, 0.97; p 0.001; directional control: SMD = 0.39; 95% CI 0.19, 0.58; *p* < 0.001). HHS did not have a statistically significant effect on mean medial–lateral COP sway and mean medial–lateral and anterior–posterior COP sway velocity during standing, and medial–lateral and anterior–posterior COP sway during walking (*p* ≥ 0.23) (see Additional file 5). For all relevant parameters, no clear asymmetries were identified in the funnel plots (see Additional file 6). In the sensitivity analyses, only the single leg stance time showed a significant change in the overall result after removing the article written by Tomac et al. [62].

## Discussion

**Fig. 4:**
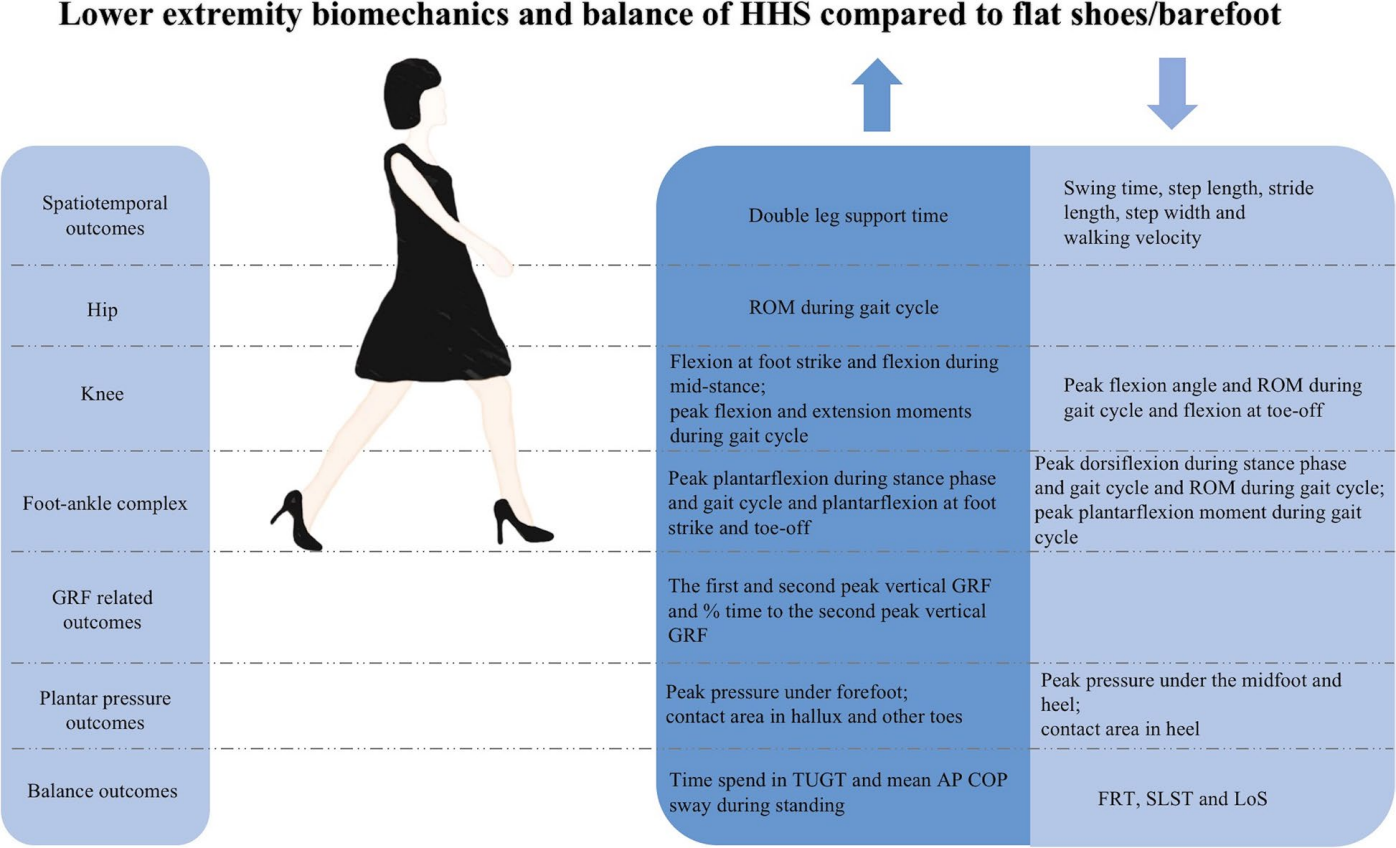
Summary of significant differences between HHS and flat shoes/barefoot biomechanics and balance found with meta-analyses. The up and down arrows represent greater and lower in HHS compared to flat shoes/barefoot, respectively. HHS high-heeled shoes; ROM range of motion; GRF ground reaction force; TUGT time up and go test; AP anterior–posterior; SLST single leg stance test; LoS limits of stability test

The purpose of this systematic review was to explore the current evidence for the effects of HHS on lower limb biomechanics and balance in females to provide guidance for future research. This study showed a full evidence map of walking gait and postural control with HHS. The findings from this systematic review suggest that HHS significantly affect lower extremity biomechanics and balance in females (Fig. 4).

Reduced area of support when walking in HHS requires the body to establish a safer and more stable gait pattern by changing gait characteristics compared with walking barefoot or in flat shoes [13, 18, 63]. Shorter step length, stride length and flight time and greater time spent with the feet in contact with the ground contribute to this requirement and attempt to counteract the instability of walking in HHS, which was depicted in our findings. Furthermore, HHS have poorer cushioning properties compared with flat shoes or trainer shoes, thus weakening the GRF absorption ability and potential kinetic energy that lower extremities already process, thereby enabling a shorter step length [26]. The instability caused by HHS ultimately leads to a significant reduction in walking speed [10]. The findings on step frequency are inconsistent, with no significant differences in step frequency in our study, possibly because the preferred walking speed of the participants had a large variation and part of the study was conducted on a treadmill at a fixed speed, whereas previous studies showed that walking at different speeds with HHS produces different walking characteristics [8, 64, 65]. In the funnel plot, the step length and step width showed a significant asymmetry, which indicates a large bias, therefore, the overall results need to be treated with caution.

The available evidence suggests that HHS have a slight effect on hip kinematics and kinetics during walking, with biomechanical changes and adaptations concentrated in the knee and ankle joints [19, 47]. As shown in our results, only ROM during gait cycle at the hip joint was significantly different between walking in HHS and flat shoes/barefoot. Compensatory changes in joint kinematics are generated to better respond to the load, as evidenced by increased flexion of the proximal joint [47], supported by the increased flexion of knee joint at foot strike and during mid-stance in this study. Surprisingly, no significant increase occurred in the peak flexion of knee when walking in HHS, perhaps because both flat shoes and barefoot were regarded as control group and involved a wide range of heel heights (3 cm to 12 cm) in our study. The knee joint was already in a more flexed position at foot strike when walking in HHS may contribute to the reduction in knee ROM during gait cycle and flexion at toe-off. Decreased ROM is indicated as a stiff joint, showing significant loss of movement, which alerts HHS wearers should pay more attention to knee joint protection to prevent musculoskeletal injuries [19, 47, 66]. Ankle maintains greater plantarflexion throughout gait cycle as a corollary to increased heel height. When HHS are worn, the resulting increased ankle plantarflexion brings the GRF vector closer to the centre of the ankle and increases the vertical impact loading in gait [16, 48]. The increased load can be reduced by adaptive changes in the kinematics of the proximal joint or through direct absorption by the soft tissues [67]. Furthermore, wearing HHS could lead to increased load on the ligaments and muscles surrounding the joints of lower limbs, which may trigger tendonitis of the muscle–tendon unit, inflammation of the bursa or progressive stretching of the ligaments around the joint [15]. Reduced ankle ROM in high-heeled gait possibly because continuous plantarflexion, which weakens the effect of the triceps surae on ankle joint and causes a reduction in the propulsion of ankle [68]. No differences were found in rearfoot movement when comparing HHS with flat shoes, potentially due to the limited available data and hence the lack of statistical power [15, 16, 69].

To the best of our knowledge, several mechanisms may contribute to the increased knee peak flexion and extension moments during high-heeled gait. One mechanism is that the increased knee flexion at foot strike places the centre of the knee relatively more forward, increasing the knee flexion torque from the vertical GRFs, hence the increased knee flexion moment may be a crucial compensatory mechanism to compensate for the relative instability caused by HHS, which also reflecting the crucial role of the knee in weight acceptance and shock absorbing [8, 13, 14, 70]. Moreover, the increase in peak knee flexion moment may partially compensate for the decrease in ankle moment, which is similar to the findings of our study [24]. The increased heel height is accompanied by an increase in the distance between the centre of the knee and ground, thus extending the lever arm of the tibia, which requires greater knee extension moment to resist GRFs [13, 19, 35]. An existing challenge is to explain the lack of significant differences in peak adduction and abduction moments in the current study. One possible explanation is the inclusion of HHS with different constructions and heel heights, making the results more variable. In addition, a threshold adaptation of HHS on knee joint may exist, where unnatural loading patterns are magnified when the heel height exceeds a certain value [15, 71, 72]. Although the peak ankle dorsiflexion moment did not differ between the two groups, the reduction in peak plantarflexion moment when walking in HHS implies the presence of eccentric activity of the muscles around the ankle playing a role in maintaining stability in the passive plantarflexion [47]. Specifically, the greater ankle plantarflexion obviously shortens fascicle length, Achilles tendon moment arm and forefoot lever arm, bringing GRFs closer to the centre of ankle and reducing the requirement for the plantarflexion moment [13, 24, 73]. The plantarflexed position of the ankle also puts the gastrocnemius and soleus muscles in a shortened position, which is detrimental to the work of the muscles [13]. Therefore, to propel the body forward, ankle joint will generate greater power [24, 74].

Body is exposed to greater impact forces earlier and the dynamic loading on the musculoskeletal system is increased in high-heeled gait [16]. The greater plantarflexion of ankle and the increased vertical GRFs exerted on the forefoot during stance as a result of the interior shift of the COG are the main contributors to these changes [15, 16]. In general, when the ankle is plantarflexed, the foot tends to supination and adduction [75]. Therefore, the increased plantarflexion of the ankle leads to reduced pronation during support with HHS, and part of the shock absorbing function of pronation may be lost, resulting in greater peak vertical GRF [15]. Peak pressure, maximum force and contact area are significantly higher in the forefoot than in the midfoot and rearfoot when walking in HHS compared with walking in flat shoes or barefoot, supporting previous studies [64, 76–78]. This finding suggests that HHS triggers a weight transfer mechanism that shifts plantar pressure to the forefoot, possibly due to the elevation of the heel causes a distinct anterior displacement of the COG of the body and reduces the cushioning effect of the arch [15, 64, 79]. The altered plantar pressure distribution may trigger arch deformation and Achilles tendon shortening, leading to discomfort and pain in the foot and the development of pathologies such as metatarsalgia and plantar fasciitis [55]. This finding also indicates that increased arch support and cushioning may improve walking ability and stability when walking in HHS, thereby providing a direction for the future design and development of HHS [80]. Specifically, the increased pressure exerted on the forefoot when walking in HHS may have a dramatic effect on the foot morphology, causing hallux valgus, varus deformity of the fifth toe and flattening feet [5, 6].

Although exploring the effects of HHS on muscle function was not possible through meta-analysis due to the lack of available data, to the authors’ knowledge, wearing HHS does alter muscle activation patterns [7, 15, 24, 80–85]. Wearing HHS is generally accepted to cause an imbalance in the intensity and timing of muscle activity around knee joint, as well as faster and greater synergistic contraction of the muscles around ankle [7, 85]. The generally increased muscle activity increases muscle energy expenditure, accompanied by increased muscle fatigue, which may induce a reduction in functional mobility during prolonged walking in HHS. As a result, the ability to control the stability of the foot and COG in response to postural perturbations is constrained, thereby increasing the risk of ankle sprains and/or falls [7, 16, 85].

Our study shows that wearing HHS increases the anterior–posterior COP sway and reduces the static and dynamic postural stability, which is consistent with previous research [7, 16, 86]. Changes in plantar somatosensory and proprioceptive afferents due to the greater plantarflexion position of the ankle are thought to be one of the main factors that contribute to changes in COP sway [87]. Furthermore, as the limits of the stability test are influenced by a combination of the neuromuscular system, the skeletomuscular system and cognition, a decrease in score indicates that a decrease in postural control is accompanied by an increased risk of falls [88]. As mentioned above, accelerated muscle fatigue is another factor that may affect postural control when walking in HHS [7, 16].

Several limitations should be considered when interpreting the findings of this review. Firstly, no subgroup analysis based on heel type and height was conducted in the meta-analysis due to the amount of available data, hence the current findings may underestimate the actual impact of high heels on biomechanics. Related to this situation, most meta-analyses were influenced by high levels of heterogeneity as well as unrobust overall results for several parameters, which indicates that caution should be taken when generalizing the effects of HHS on lower limb biomechanics and balance. In addition, three studies included older women, where age-related changes in body structure modified the biomechanical performance [23, 60, 65]. Older women generally have more experience wearing HHS, which contributed to the variability in outcomes. Long-term wearing of HHS also brings about adaptive changes in the biomechanics and control strategies of the human body [45, 89, 90]. Knowledge of the adaptive alterations caused by long-term wearing of HHS may provide better theoretical support for footwear design, offer guidance for novices in choosing HHS and effectively prevent high heel-related injuries [46]. Furthermore, various heel heights can affect everyone differently due to individual differences. Future studies should attempt to investigate more specific heel heights to determine the exact value/range of the threshold adaptation and to minimize the local damage caused by wearing HHS.

## Conclusion

Walking in HHS exerts significant effects on the kinematics and kinetics of the knee and foot–ankle complex, as evidenced by the gait profiles altered in this study. Elevated heels caused the body to be exposed to greater GRFs earlier, accompanied by an anterior shift in plantar pressure. Furthermore, wearing HHS reduced static and dynamic postural control significantly. This meta-analysis provides comprehensive biomechanical data that may inform future efforts to mitigate the negative effects of wearing HHS on women in clinical practice. Moreover, more studies involving different heel heights and heel areas and long-term follow-up design are needed to confirm the changes in walking and balance caused by wearing HHS, as well as long-term neuromuscular adaptations, to provide a theoretical basis for maximizing the protection of women’s foot health and preventing HHS-related injuries.

## Supplementary Information

**Additional file 1.** 

**Additional file 2.** 

**Additional file 3.** 

**Additional file 4.** [91–123].

**Additional file 5.** 

**Additional file 6.** 

## Data Availability

All data analysed during this study are included in its supplementary information files.

## Abbreviations

CI: Confidence interval
COG: Centre of gravity
COP: Centre of pressure
ES: Effect size
GRF: Ground reaction force
HHS: High-heeled shoes
QI: Quality Index
ROM: Range of motion
SMD: Standardised mean difference
