# Supplementary material for: Effects of high-heeled shoes on lower extremity biomechanics and balance in females: a systematic review and meta-analysis
Source: BMC Public Health. 2023 Apr 20;23:726. doi: 10.1186/s12889-023-15641-8 (PMC10120101; doi:10.1186/s12889-023-15641-8)
Supplement: Supplementary file 1 — Additional file 1. [file 12889_2023_15641_MOESM1_ESM.pdf]

Additional file 1

Search terms and strategies

Full search terms and strategies among PubMed Medline, Cochrane, EMBASE, CINAHL Complete and Web of Science electronic databases.

#1 (positive heel inclination OR high heel OR high-heel OR High heels OR high-heeled OR high heeled OR wedge heel OR platform heel OR platform shoe OR stiletto OR elevator shoe OR heel height)

#2 (Walk* OR 'Walking'[Mesh] OR 'Gait'[Mesh] OR ambulat* OR 'Locomotion'[Mesh])

#3 ('Kinetics'[Mesh] OR 'Biomechanical Phenomena'[Mesh] OR 'Lower Extremity'[Mesh] OR spatiotemporal OR temporospatial OR 'range of motion, Articular'[Mesh] OR 'Ankle'[Mesh] OR 'Knee'[Mesh] OR 'Hip'[Mesh] OR 'Electromyography'[Mesh] OR EMG OR IEMG OR 'Posture'[Mesh] OR 'Postural Balance'[Mesh])

#1 AND #2 AND #3

No restrictions were set on literature type or publication status.
