# Supplementary material for: Effects of high-heeled shoes on lower extremity biomechanics and balance in females: a systematic review and meta-analysis
Source: BMC Public Health. 2023 Apr 20;23:726. doi: 10.1186/s12889-023-15641-8 (PMC10120101; doi:10.1186/s12889-023-15641-8)
Supplement: Supplementary file 2 — Additional file 2. [file 12889_2023_15641_MOESM2_ESM.pdf]

Additional file 2

Specific inclusion and exclusion criteria

Inclusion criteria:

(1) female participants (<60 years old)

(2) healthy participants with no orthopaedic or neurological diseases

(3) experimental design with a control group (either walking in flat shoes or barefoot)

Exclusion criteria:

(1) included only elderly adults (older than 60 years old) or patients with hallux valgus

(2) did not use HHS (wide-heeled shoes and prefabricated heel lifts were excluded)

(3) did not involve level walking (or inclusion of studies where only balance was evaluated)

(4) studied functional testing or intervention exercises only

(5) reviews and conference papers were also excluded
