# Supplementary material for: Effects of high-heeled shoes on lower extremity biomechanics and balance in females: a systematic review and meta-analysis
Source: BMC Public Health. 2023 Apr 20;23:726. doi: 10.1186/s12889-023-15641-8 (PMC10120101; doi:10.1186/s12889-023-15641-8)
Supplement: Supplementary file 3 — Additional file 3. [file 12889_2023_15641_MOESM3_ESM.pdf]

Additional file 3

Modified Downs and Black's Quality Index

Category	Item	Question	Application to this review
Study reporting	1	1. Is the hypothesis/aim/objective of the study clearly described?	Score of "1" if hypothesis/aim/objective described;<br>"0" for no description.
	2	2. Are the main outcomes to be measured clearly described in the introduction or methods section?	Score of "1" if main outcome measures described in introduction or methods;<br>"0" for no description.
	3	3. Are the characteristics of the participants included in the study clearly described?	Score of "1" if clear inclusion and exclusion criteria described;<br>"0" for no description.
	4	4. Comparisons clearly described	Score of "1" if comparisons described;<br>"0" for no description.
	6	5. Are the main findings of the study clearly described?	Score of "1" if main findings clearly described;<br>Score "0" for no description.
	7	6. Does the study provide estimates of the random variability in the data for the main outcomes?	Score of "1" if any of these measures of variability given;<br>Score "0" for no description.
	10	7. Have actual probability values been reported (e.g. 0.002 rather than <0.05) for the main outcomes except where probability value is less than 0.001?	Score of "1" if actual probability values described;<br>Score "0" for no description.
External validity	11	8. Were the subjects asked to participate in the study representative of the entire population from which they were recruited?	Score of "1" if participants recruited were from the community.<br>Score "0" for no description or if the number of participants was less than 10.

	12	9. Were those subjects who were prepared to participate representative of the entire population from which they were recruited?	Score of "1" if participants who contacted were from the community;<br>Score "0" for no description.
Internal validity	16	10. If any of the results of the study were based on "data dredging", was this made clear?	Score of "1" for clearly mentioning the outcome measures planned;<br>Score "0" if data dredging was there.
	18	11. Were the statistical tests used to assess the main outcomes appropriate?	Score of "1" if appropriate statistical tests used;<br>Score "0" for no description.
	20	12. Were the main outcome measures used accurate (valid and reliable)?	Score of "1" if reference given for validity or reliability of the outcome measures used;<br>Score "0" for no description.
	22	14. Participants' recruited over the same period	Score of "1" if the time participants recruited described;<br>Score "0" for no description.
	23	15. Participants randomised to intervention/experimental conditions	Score of "1" if the time participants randomised to intervention/experimental conditions;<br>Score "0" for no description or participants completed the intervention/experimental conditions in the prescribed order.
Power	27	16. Were appropriate power calculations reported?	Score of "1" if a power or a sample size calculation was provided;<br>Score "0" for no description or no explanation whether the number of participants was appropriate.
