# Supplementary material for: Effects of high-heeled shoes on lower extremity biomechanics and balance in females: a systematic review and meta-analysis
Source: BMC Public Health. 2023 Apr 20;23:726. doi: 10.1186/s12889-023-15641-8 (PMC10120101; doi:10.1186/s12889-023-15641-8)
Supplement: Supplementary file 4 — Additional file 4. [91–123]. [file 12889_2023_15641_MOESM4_ESM.pdf]

Additional file 4

Characteristics of the reviewed studies

Table 1 Characteristics of the reviewed studies.

Studies	Sample size	Age (years)	Body height (cm)	Body mass (kg)	HHS wearing experience	HHS height	Comparisons	Walking speed (m/s)	Outcome
Adrian et al., 1966 [91]	11	College women	/	/	Occasionally	8.57 cm	BF vs. 8.57 cm	/	Spatiotemporal and kinematics.
Alkjær et al., 2012 [27]	11	27.5 ± 5.4	170 ± 4	58.1 ± 5.1	Experienced	9 cm	BF vs. 9 cm	1.11 m/s	Spatiotemporal, kinematics and muscle function.
Baekema et al., 2012 [8]	15	23.8 ± 4.4	165.5 ± 7.1	60.9 ± 8.7	2.1 ± 1.6 days/week, 5.1 ± 1.6 hours/episode, 7.6 ±1.5 cm, 7.6 ± 4.4 years	0.8 cm, 5.1 cm and 8.9 cm	0.8 cm vs. 5.1 cm vs. 8.9 cm	1.3 m/s	Spatiotemporal, kinematics and kinetics.
Blanchette et al., 2011 [92]	15	24.5 ± 2.5	161 ± 5	56.2 ± 10.0	Experienced	1.27 cm, 6.35 cm and 9.53 cm	1.27 cm vs. 6.35 cm vs. 9.53 cm	Low: 1.38 m/s; Medium: 1.31 m/s; High: 1.24 m/s	Spatiotemporal, kinematics and kinetics.
Cha, 2020 [89]	54<br>Group A: 18; Group B: 17; Group C: 19	Group A: 20.11 ± 1.59; Group B: 20.11 ± 1.59; Group C: 19.66 ± 1.14	Group A: 167.45 ± 3.13; Group B: 166.02 ± 2.96; Group C: 165.78 ± 3.87	Group A: 53.29 ± 4.06; Group B: 55.00 ± 3.35; Group C: 54.11 ± 5.09	Group A: > 6 cm, > 12 months, 4 times/week; Group B: > 4-5 cm, > 12 months, 4 times/week; Group C: < 3 cm, > 12 months, 4 times/week	7 cm	Experienced vs. inexperienced	/	Balance.
Chen et al., 2020 [31]	41<br>Experienced: 20<br>Inexperienced: 21	Experienced: 23.05 ± 2.24; Inexperienced: 25.05 ± 1.63	Experienced: 163 ± 5; Inexperienced: 163 ± 5 m	Experienced: 56.33 ± 6.94; Inexperienced: 57.51 ± 7.87	Experienced: > 4 cm, > 2 times/week, > 8 hours/day, > 1 years, 28.33 ± 10.13 hours/week; Inexperienced: < 1 time/ week, 2.19 ± 4.61 hours/week	3.9 cm, 7.0 cm and 10.1 cm	0.8 cm vs. 3.9 cm vs. 7.0 cm vs. 10.1 cm	/	Balance.

Chhoeum et al., 2020 [93]	7	20.7 ± 0.8	157.7 ± 4.4	50.4 ± 2.2	Non-regular	9 cm (three different types of shoes)	Flat (1 cm) vs. sneakers (3 cm) vs. chunky heel (9 cm) vs. classic pump heel (9 cm) vs. stiletto heel (9 cm)	Self-select speed: 0.67 ± 0.08 m/s; Fast speed: 1.42 ± 0.06 m/s	Kinematics.
Chien et al., 2013 [10]	15	24.4 ± 3.4	158.9 ± 5.7	49.2 ± 5.1	> 3 cm, > 3 times/week, 6 hours/day, 2 years	3.9 cm, 6.3 cm and 7.3 cm	BF vs. 3.9 cm vs. 6.3 cm vs.7.3 cm	BF: 1.15 ± 0.11 m/s; Low: 1.09 ± 0.09 m/s; Medium: 1.09 ± 0.09 m/s; High: 1.07 ± 0.09 m/s	Spatiotemporal and kinetics.
Chien et al., 2014 [45]	30 Experienced: 15; Inexperienced: 15	Experienced: 24.4 ± 3.4; Inexperienced: 24.9 ± 4.1	Experienced: 158.9 ± 5.7; Inexperienced: 161.0 ± 4.2	Experienced: 49.2 ± 5.1; Inexperienced: 50.3 ± 3.7	Experienced: > 3 cm, > 3 times/week, 6 hours/day, > 2 years; Inexperienced: < 2 times/ month	7.3 cm	Experienced vs. inexperienced	Experienced: 1.07 ± 0.09 m/s; Inexperienced: 1.08 ± 0.10 m/s	Spatiotemporal and kinetics.
Chien et al., 2013 [94]	15	24.4 ± 3.4	158.9 ± 5.7	49.2 ± 5.1	> 3 cm, > 3 times/week, 6 hours/day, 2 years	3.9 cm, 6.3 cm and 7.3 cm	BF vs. 3.9 cm vs. 6.3 cm vs.7.3 cm	Self-selected speed	Kinematics and kinetics.
Chien et al., 2014 [46]	30 Experienced: 15; Inexperienced: 15	Experienced: 24.4 ± 3.4; Inexperienced: 24.9 ± 4.1	Experienced: 158.9 ± 5.7; Inexperienced: 161.0 ± 4.2	Experienced: 49.2 ± 5.1; Inexperienced: 50.3 ± 3.7	Experienced: > 3 cm, > 3 times/week, 6 hours/day, > 2 years; Inexperienced: < 2 times/month	7.3 cm	Experienced vs. inexperienced	Experienced: 1.07 ± 0.09 m/s; Inexperienced: 1.08 ± 0.10 m/s	Spatiotemporal, kinematics and kinetics.
Choi et al., 2006 [95]	15	23.7 ± 1.1	158.7 ± 2.9	53.9 ± 4.9	/	3 cm, 6 cm and 9 cm	BF vs. flat (0 cm) vs. 3 cm vs. 6 cm vs. 9 cm	Self-selected speed	Spatiotemporal and kinematics.
Cong et al., 2011 [76]	10	26.4 ± 2.8	162 ± 4	51.7 ± 4.9	Experienced	3 cm, 5 cm and 7 cm	3 cm vs. 5 cm vs. 7 cm	110 steps/min	Kinetics.
Cronin et al., 2012 [18]	19 Experienced: 9;	Experienced: 25 ± 7;	Experienced: 168 ± 7;	Experienced: 65 ± 17;	Experienced: > 5 cm, > 40 hours/week, > 2 years;	11 cm ± 2 cm or 6 ± 1% of	Inexperienced BF vs. experienced BF vs. experienced	Inexperienced BF: 1.19 ± 0.05 m/s;	Spatiotemporal and muscle function.

	Inexperienced: 10	Inexperienced: 25 ± 4	Inexperienced: 166 ± 5	Inexperienced: 60 ± 7	Inexperienced: < 10 hours/week	stature	HHS	Experienced BF: 1.22 ± 0.04 m/s; Experienced HHS: 1.20 ± 0.03 m/s	
Delafontaine et al., 2019 [96]	13	Young adults	/	/	/	6 cm and 9 cm	Without HHS vs. 6 cm vs. 9 cm	Self-selected speed	Spatiotemporal and balance.
Di Sipio et al., 2018 [57]	21 Short shoe size group (SH): 11; Long shoe size group (LO): 10	25.95 ± 3.60	165.95 ± 6.45	59.14 ± 5.95	1-3 times/week, < 15 hours/week	12 cm	BF vs. 12 cm HHS from foot/shoe size vs. HHS based on foot/shoe size	SH group: BF: 1.18 ± 0.10 m/s; HHS: 1.14 ± 0.10 m/s; HHS_{12}: 1.06 ± 0.12 m/s LO group: BF: 1.16 ± 0.05 m/s; HHS: 1.09 ± 0.09 m/s; HHS_{12}: 1.10 ± 0.09 m/s	Spatiotemporal, kinematics and kinetics.
Ebbeling et al., 1994 [16]	15 Experienced: 7; Inexperienced: 8	23.3 ± 2.9	165 ± 4	60.1 ± 6.2	Experienced: > 3 times/week, > 8 hours/day; Inexperienced: < 2 times/month	1.25 cm, 3.81 cm, 5.08 cm and 7.62 cm	1.25 cm vs. 3.81 cm vs. 5.08 cm vs. 7.62 cm	1.17 m/s ± 5%	Kinematics and kinetics.
Eisenhardt et al., 1996 [12]	30	18-30 years	/	/	/	1.75 cm, 3.12 cm, 5.72 cm and 8.74 cm	BF vs. 1.75 cm vs. 3.12 cm vs. 5.72 cm vs. 8.74 cm	100 ± 10 steps/min	Spatiotemporal and kinetics.
Esenyel et al., 2003 [13]	15	23-42	172 ± 6.8	66 ± 13	/	6 cm	Low-heeled sports shoe (1 cm) vs. HHS (6 cm)	LHS: 1.294 ± 0.154 m/s; HHS: 1.222 ± 0.121 m/s	Spatiotemporal, kinematics and kinetics.

Foster et al., 2012 [81]	18	25.3 ± 4.0	160 ± 10	58.3 ± 8.9	5.3 ± 5.1 times/month, 5.1 (range: 1-9, 0 = no experience, 10 = highly experienced)	1.3 cm and 9.5 cm	1.3 cm vs. 9.5 cm	1.2 m/s and 1.4 m/s	Spatiotemporal, kinematics, kinetics and muscle function.
Gastwirth et al., 1991 [97]	5	26.1	162.56	58.86	4.85 days/month, 11.14 years	4.2 cm	BF vs. athletic shoes (0.2 cm) vs. HHS (4.2 cm)	/	Spatiotemporal, kinematics and kinetics.
Gehlsen et al., 1986 [17]	19	24.47 ± 3.85	166.74 ± 6.04	60.69 ± 5.85	/	6.0-10.7 cm	BF vs. running shoes (1.2-1.5 cm) vs. HHS (6.0-10.7 cm)	1.12 m/s	Spatiotemporal and kinematics.
Gollnick et al., 1964 [50]	5	/	/	/	/	3-7 cm and 7-11 cm	BF vs. 3-7 cm vs. 7-11 cm	0.69 m/s and 120 steps/min	Kinematics.
Gu et al., 2011 [98]	12	22 (20-25)	161 (158-165)	49 (45-53)	> 2 years	4.5 cm and 8.5 cm	Flat vs. 4.5 cm vs. 8.5 cm	/	Kinetics.
Guo et al., 2012 [59]	13	22 ± 0.8	/	/	/	7.8 cm	Casual shoes vs. 7.8 cm (narrow based heels) vs. 7.8 cm (wide based heels)	1.0 m/s and 1.5 m/s	Kinetics.
Hapsari & Xiong, 2015 [49]	30 Experienced: 10; Inexperienced: 20	Experienced: 20.60 ± 1.62; Inexperienced: 20.05 ± 1.00	Experienced: 159.87 ± 4.98; Inexperienced: 163.86 ± 5.06	Experienced: 55.17 ± 6.03; Inexperienced: 56.74 ± 11.04	Experienced: 3.80 ± 1.87 times/week; Inexperienced: 0.25 ± 0.41 times/week	4 cm, 7 cm and 10 cm	Flat (1 cm) vs. 4 cm vs. 7 cm vs. 10 cm	/	Muscle function and balance.
Ho et al., 2012 [99]	11	25.0 ± 3.1	161.6 ± 5.4	55.5 ± 7.1	5 ± 2.3 times/month	1.27 cm, 6.35 cm and 9.53 cm	1.27 cm vs. 6.35 cm vs. 9.53 cm	Low: 1.38 ± 0.175 m/s; Medium: 1.38 ± 0.178 m/s; High: 1.39 ± 0.173 m/s	Kinematics and kinetics.
Hong et al., 2005 [77]	20	25.4 ± 3.8	157.8 ± 5.0	50.5 ± 4.2	/	1.0 cm, 5.1 cm and 7.6 cm	1.0 cm vs. 5.1 cm vs. 7.6 cm	1.3 m/s	Kinetics.

Hong et al., 2013 [82]	15	24.5 (20-30)	159.3 (155-168)	49.6 (44-57)	< 2 times/month	1.0 cm, 5.1 cm and 7.6 cm	1.0 cm vs. 5.1 cm vs. 7.6 cm	1.3 m/s	Kinematics and muscle function.
Hyun & Ryew, 2018 [100]	9	27.88 ± 2.71	165.94 ± 2.78	56.56 ± 6.42	/	6 cm	BF vs. 6 cm	/	Kinetics.
Jandova et al., 2019 [64]	30	21.8 ± 2.09	166 ± 3	55.7 ± 4.05	Occasionally	7 cm	Flat vs. 7 cm	0.97 m/s (slow speed) and 0.56 m/s (very slow speed)	Spatiotemporal and kinetics.
Joseph, 1968 [52]	6	18-23	150-175	50-67	/	1-2.5 cm and 5.5-8 cm	Low (1-2.5 cm) vs. high (5.5-8 cm)	Self-selected speed, 54-58 steps/minute	Muscle function.
Kermani et al., 2018 [53]	40<br>High: 20; Low: 20	High: 29.63 ± 6.25; Low: 25.90 ± 6	High: 162 ± 6.167; Low: 164 ± 5.96	High: 59.52 ± 6.04; Low: 57.10 ± 7.24	5 days/week, > 2 years, 8 hours/day, averagely 5 hours in standing or walking situations and 3 hours in sitting situation in each day	1-3 cm and 5-8 cm	HHS women wearing HHS vs. HHS women BF vs. LHS women wearing LHS vs. LHS women BF	Self-selected speed	Muscle function.
Kerrigan et al., 1998 [19]	20	34.6 ± 9.3	167 ± 6	61.76 ± 7.6	Experienced	6.0 (1.0) cm	BF vs HHS (6.0 (1.0) cm)	Self-selected speed	Kinematics and kinetics.
Kilby & Newell, 2012 [101]	20<br>Ballet dancer: 10; Exercising students: 10	Dancer: 21.5 ± 3.1; Student: 21 ± 1.8	Dancer: 165 ± 3.8; Student: 162.6 ± 5.1	Dancer: 58.7 ± 6.3; Student: 58.9 ± 8.4	Occasionally	7.5 cm	BF vs. 7.5 cm (HHS) vs. 7.5 cm (toes standing)	/	Balance.
Kim & Lee, 2016 [102]	12	23.1 ± 2.0	162.4 ± 4.9	54.4 ± 8.5	Inexperienced	7 cm	BF vs. 7 cm	BF: 0.64 ± 0.09 m/s; HHS: 0.59 ± 0.09 m/s	Muscle function.
Lee, 2014 [71]	14	21.2 ± 0.8	163.2 ± 3.1	57.7 ± 6.0	/	3 cm, 5 cm and 7 cm	3 cm vs. 5 cm vs. 7 cm	Self-selected speed	Kinematics.

Lee et al., 2001 [63]	5	/	/	/	/	4.5 cm and 8 cm	0 cm vs. 4.5 cm vs. 8 cm	1.11 m/s	Kinetics and muscle function.
Lee et al., 2016 [58]	15	24.67 ± 3.54	162.20 ± 3.91	54.96 ± 6.67	> 3 times/week, 3 years	9 cm	Flat (1.1 cm) vs. 9 cm	Self-selected speed	Kinetics.
Lee et al., 2020 [47]	30 Experienced: 15; Inexperienced: 15	Experienced: 24.7 ± 3.53; Inexperienced: 22.7 ± 3.15	Experienced: 162.2 ± 3.91; Inexperienced: 163.2 ± 5.88	Experienced: 54.9 ± 6.66; Inexperienced: 56.8 ± 8.45	Experienced: > 3 years, > 3 times/week, Inexperienced: never worn HHS or have been wearing them < 1 time/month.	9 cm	Flat (1.1 cm) vs. 9 cm	Self-selected speed	Spatiotemporal, kinematics, kinetics and balance.
Lee & Hong, 2005 [21]	10	23 (20-28)	160 (156-162)	50 (47-53)	4 subjects had worn HHS 2-5 times/week for at least 1 year. The other 6 had relatively limited experience.	5.1 cm and 7.6 cm	Flat (1.0 cm) vs. 5.1 cm vs. 7.6 cm	1.3 m/s	Kinetics.
Lee & Li, 2014 [103]	15	24.67 ± 3.54	162.2 ± 3.91	54.96 ± 6.67	> 3 times/week, 3 years	3 cm and 9 cm	Flat (1.1 cm) vs. 3 cm vs. 9 cm	Flat: 125 ± 14.1 steps/min; 3 cm: 118.8 ± 17.7 steps/min; 9 cm: 116.5 ± 19.7 steps/min	Spatiotemporal and kinematics.
Mandato & Nester, 1999 [104]	35	25.5 (23-32)	/	60.10 (44.45-95.25)	Experienced	5.08 cm and 7.62 cm	Sneaker vs. 5.08 cm vs. 7.62 cm	/	Kinetics.
Mapelli et al., 2012 [54]	11	24.4	/	/	/	＞8 cm	Flat (< 1 cm) vs. HHS (> 8 cm)	Self-selected speed	Kinetics.
Massoud, 2021 [105]	14	22.57 ± 0.97	161 ± 4	53.1 ± 6.6	/	3 cm, 5 cm, 7 cm, 9 cm and 12 cm	BF vs. 3 cm vs. 5 cm vs. 7 cm vs. 9 cm vs. 12 cm	Self-selected speed	Spatiotemporal.

McBride et al., 1991 [55]	11	26.6 ± 6.7	162.8 ± 5.2	64.1 ± 9.1	/	6.8 (0.8) cm	BF vs. HHS (6.8 (0.8) cm)	BF: 1.34 ± 0.18 m/s; HHS: 1.31 ± 0.18 m/s	Spatiotemporal, kinematics and kinetics.
Melvin et al., 2019 [106]	16	27.4 ± 7.73	163.16 ± 5.47	57.6 ± 5.25	/	3.5 cm, 4.5 cm, 5.5 cm, 6.5 cm and 7.5 cm	3.5 cm vs. 4.5 cm vs. 5.5 cm vs. 6.5 cm vs. 7.5 cm	1.2 m/s (± 5%)	Kinetics.
Merrifield, 1971 [51]	12	18-20	152.4-167.64	54.43-65.77 kg	/	6.4-7.6 cm	Flat (0.5-1.5 cm) vs. HHS (6.4-7.6 cm)	Self-selected speed	Spatiotemporal and kinematics.
Mika et al., 2012 [23]	46 Young: 31; Middle-aged: 15	Young: 20-25; Middle-aged: 45-55	Young: 167.6 ± 5.8; Middle-aged: 161.3 ± 4.2	Young: 60.35 ± 6.49; Middle-aged: 65.8 ± 6.2	< 1 time/month	4 cm and 10 cm	BF vs. 4 cm vs. 10 cm	Young: BF: 1.34 ± 0.6 m/s; 4 cm: 1.3 ± 0.6 m/s; 10 cm: 1.26 ± 0.7 m/s; Middle-aged: BF: 1.33 ± 0.5 m/s; 4 cm: 1.24 ± 0.6 m/s; 10 cm: 1.21 ± 0.6 m/s	Spatiotemporal, kinematics and muscle function.
Mika et al., 2012 [107]	46 Young: 31; Middle-aged: 15	Young: 20-25; Middle-aged: 45-55	Young: 167.6 ± 5.8; Middle-aged: 161.3 ± 4.2	Young: 60.35 ± 6.4); Middle-aged: 65.8 ± 6.2	< 1 time/month	4 cm and 10 cm	BF vs. 4 cm vs. 10 cm	Young: BF: 1.34 ± 0.6 m/s; 4 cm: 1.3 ± 0.6 m/s; 10 cm: 1.26 ± 0.7 m/s; Middle-aged: BF: 1.33 ± 0.5 m/s; 4 cm: 1.24 ± 0.6 m/s; 10 cm: 1.21 ±	Kinematics and muscle function.

								0.6 m/s	
Mika et al., 2016 [30]	31	22-27	168.6 ± 5.1	57.1 ± 11.8	Occasionally; < 1 time/month	4 cm and 10 cm	BF vs. 4 cm vs. 10 cm	/	Balance.
Moraes et al., 2012 [108]	15	23.4 ± 3.6	/	/	/	2-4 cm, 5-7 cm and 9-11 cm	BF vs. slipper vs. tennis shoe vs. small heeled shoe vs. HHS	110 steps/minute	Muscle function.
Nyska et al., 1996 [56]	10	33.6 (21-60)	165	56.1 (54-67)	/	1.7 (1-2.5) cm and 6 (4.5-8) cm	Low (1.7 (1-2.5) cm) vs. high (6 (4.5-8) cm)	1.11 m/s	Spatiotemporal and kinetics.
Owen et al., 2017 [109]	10	25 ± 3	166.0 ± 7.0	65 ± 10	Experienced	3.7 (1) cm and 7.1 (1.7) cm	BF vs. Mid-heel (3.7 cm) vs. HHS (7.1 cm)	BF: 1.40 ± 0.22 m/s; Mid-heel: 1.41 ± 0.15 m/s; HHS: 1.37 ± 0.18 m/s	Spatiotemporal and kinematics.
Park et al., 2010 [83]	17	22.06 ± 1.2	161.06 ± 4.51	52.88 ± 4.41	Experienced	3 cm and 7 cm	BF vs. 3 cm vs. 7 cm	0.56 m/s	Muscle function.
Park et al., 2019 [110]	10	24 ± 2.72	159.3 ± 3.02	50.5 ± 4.25	6.4 ± 1.65 cm, 7.0 ± 4.15 hours/day, 3.2 ± 1.84 days/week, 4.8 ± 1.14 years	9 cm	Narrow (9 cm) vs. moderate (9 cm) vs. wide (9 cm) vs. wedge (9 cm)	1 m/s and 1.25 m/s	Spatiotemporal and kinetics.
Penny et al., 2018 [60]	22	40 (21-61)	167 (159-175)	62 (50-86)	Experienced	7.8 (0.5) cm and 8 (2) cm.	Orthotic stiletto (10 cm) vs. standard stiletto with no insoles	1.11 m/s	Kinetics.

							(8 cm) vs. training shoe.		
Polome et al., 2020 [111]	6	/	/	/	/	6 cm	BF vs. city shoes (flat sole ballerin) vs. classic shoes (6 cm)	0.83 m/s and 1.39 m/s	Balance.
Pratihast et al., 2018 [112]	15	24.2 ± 1.5	/	/	/	4 cm, 6 cm and 10 cm	4 cm vs. 6 cm vs. 10 cm	Self-selected speed	Muscle function.
Rao et al., 2013 [113]	31	28 ± 6	163 ± 5	57.1 ± 6.1	Occasionally, not daily users	1.91 cm and 7.62 cm	1.91 cm vs. 7.62 cm	1.91 cm: 1.03 ± 0.14 m/s; 7.62 cm: 0.99 ± 0.12 m/s	Spatiotemporal, kinematics and kinetics.
Rezgui et al., 2015 [114]	5	27 ± 3	/	/	Experienced	7.2 cm, 9 cm, 12.2 cm and 12.5 cm	BF vs. 7.2 cm thin heel vs. 12.2 cm thin heel vs. 9 cm wedged shoe vs. 12.5 cm wedged shoe	Self-selected speed	Kinetics.
Shang et al., 2020 [61]	20	20.89 ± 3.04	161.0 ± 0.05	53.80 ± 5.75	Inexperienced	3 cm, 6 cm and 8.2 cm	Flat vs. 3 cm (thick and thin) vs. 6 cm (thick and thin) vs. 8.2 cm (thick and thin)	1 m/s	Spatiotemporal and kinetics.
Simonsen et al., 2012 [24]	14	27 (21-38)	169 (158-183)	63 (48-85)	Experienced: 3.8 (1.5-7.0) times/week; Inexperienced: 0.5 (0-1)/week	9 cm	BF vs. 9 cm	1.11 m/s (± 10%)	Spatiotemporal, kinematics, kinetics and muscle function.
Sinclair et al., 2019 [48]	24 Experienced: 12; Inexperienced: 12	Experienced: 30.54 ± 5.55; Inexperienced: 29.24 ± 4.78	Experienced: 165 ± 8; Inexperienced: 166 ± 11	Experienced: 63.42 ± 6.73; Inexperienced: 65.27 ± 5.98	Experienced: > 5 cm, > 5 times/week, > 2 years	4 cm, 7 cm and 10 cm	Trainer shoes vs. 4 cm vs. 7 cm vs. 10 cm	1.5 m/s (± 5%)	Spatiotemporal, kinetics and muscle function.
Snow & Williams, 1994 [15]	11	/	/	/	> 4 hours/day, 3 days/week, > 1 year	1.91 cm, 3.81 cm and 7.62 cm	1.91 cm vs. 3.81 cm vs. 7.62 cm	1.4 m/s	Spatiotemporal, kinematics and kinetics.

Snow et al., 1992 [115]	45	37.9 ± 7.8	162.0 ± 6.3	62.1 ± 7.9	14 of them normally wore to work shoes of medium heel height (3.81-5.08 cm) and 31 normally wore HH (> 5.33 cm); 9.1 hours/day, 4.6 days/week, 14.2 years	1.91 cm, 5.08 cm and 8.26 cm	BF vs. 1.91 cm vs. 5.08 cm vs. 8.26 cm	1.4 m/s (± 5%)	Spatiotemporal, kinematics and kinetics.
Speksnijder et al., 2005 [22]	10	22 (21-24)	167 (158-175)	60 (52-73)	Experienced	1.95 (1.06) cm and 5.91 (1.03) cm	1.95 (1.06) cm vs. 5.91 (1.03) cm	Self-selected speed	Kinetics.
Stefanyshyn et al., 2000 [116]	13	40.6 ± 8.3	164.1 ± 5.6	67.7 ± 12.3	Regularly	3.7 cm, 5.4 cm and 8.5 cm	Flat (1.4 cm) vs. 3.7 cm vs. 5.4 cm vs. 8.5 cm	1.4 ± 0.2 m/s	Kinematics, kinetics, and muscle function.
Steiner & Boyer, 2021 [70]	13	26 ± 7.8	159 ± 2	55.4 ± 33.8	2 frequently, 8 occasionally and 3 seldom	8.5 cm	Flat vs. 8.5 cm	Self-selected speed: 1.31 ± 0.11 m/s; Fast speed: 1.61 ± 0.12 m/s	Kinematics and kinetics.
Sun & Gu, 2014 [117]	18	23.13 ± 1.55	165.8 ± 3.64	52.21 ± 2.93	> 2 years	6.6 cm	Flat (0.8 cm) vs. 6.6 cm	1 m/s	Kinetics.
Titchenal et al., 2015 [65]	14	20-51	168 ± 6	57.4 ± 4.6	/	3.8 cm and 8.3 cm	Athletic shoe vs. 3.8 cm vs. 8.3 cm	Athletic shoe: 1.41 ± 0.14 m/s; 3.8 cm: 1.28 ± 0.10 m/s; 8.3 cm: 1.17 ± 0.13 m/s	Spatiotemporal, kinematics and Kinetics.
Tomac et al., 2020 [62]	33	20.7 ± 2.5	/	/	Experienced: > 5 cm, > 40 hours/week, > 2 years; Inexperienced: < 10 hours/week	4 cm and 10 cm	BF vs. 4 cm vs. 10 cm	/	Balance.
Truszcynska et al., 2016 [118]	71	21.09 ± 2.91	167.0 ± 4.68	57.2 ± 8.32	Regularly; < 1 time/week	4 cm and 10 cm	BF vs. 4 cm vs. 10 cm	/	Balance.

Velazquez et al., 2021 [119]	3	26 ± 2.5	160 ± 20	75 ± 1.3	Inexperienced	2 cm, 6 cm and 10 cm	BF vs. 2 cm vs. 6 cm vs. 10 cm	BF: 1.01 ± 0.01 m/s; 2 cm: 1 ± 0.01 m/s; 6 cm: 0.99 ± 0.01 m/s; 10 cm: 0.94 ± 0.01 m/s	Spatiotemporal and kinematics.
Wan et al., 2018 [9]	24 Experienced: 12; Inexperienced: 12	Experienced: 24.6 ± 2.1; Inexperienced: 23.2 ± 2.3	Experienced：159.0 ± 3.8; Inexperienced: 160.5 ± 4.7	Experienced：52.53 ± 6.94; Inexperienced: 49.15 ± 4.15	Experienced: > 5cm, > 3 times/week, > 18 hours/week, > 2 years; Inexperienced: < 1 time/week	5 cm, 8 cm and 10 cm	1 cm vs. 5 cm vs. 8 cm vs. 10 cm	/	Balance.
Wang et al., 2001 [120]	10	21-23	168.37 ± 4.23	59.21 ± 5.62	/	7.5 cm	Flat (1.3 cm) vs. running shoes (2.5 cm) vs. HHS (7.5 cm)	HHS: 109.75 ± 7.36 steps/min; Flat shoes: 104.88 ± 6.40 steps/min; Running shoes: 102.75 ± 7.17 steps/min	Spatiotemporal and kinetics.
Wang et al., 2016 [121]	12	32.5 ± 4.2	/	/	> 6 hours/day, > 3 years	4 cm and 10 cm	BF vs. 4 cm vs. 10 cm	Approximately 1 m/s	Kinematics.
Wang et al., 2018 [122]	15	23 ± 2.5	165 ± 30	51 ± 3.6	Inexperienced	5 cm	BF vs. 5 cm	A normal speed	Kinematics.
Wang et al., 2018 [123]	15	22.51 ± 2.24	163.25 ± 2.22	52.10 ± 3.58	/	5 cm	BF vs. 5cm	0.69 m/s and 1.39 m/s	Kinematics.
Yick et al., 2018 [80]	5	21.80 ± 4.09	158 ± 53	50.20 ± 4.15	5.20 ± 4.09 years, > 6 hours/day, > 2 days/week	2 cm, 5 cm and 8 cm	2 cm vs. 5 cm vs. 8 cm	Self-selected speed	Muscle function.

Yu et al., 2021 [84]	12	20.3 ± 0.08	176 ± 3	58.2 ± 3.3	Experienced	3 cm, 6 cm, 13 cm and 18 cm	Flat (0 cm) vs. 3 cm vs. 6 cm vs. 13 cm vs. 18 cm	Flat: 1.48 ± 0.08 m/s; 3 cm: 1.38 ± 0.08 m/s; 6 cm: 1.30 ± 0.03 m/s; 13 cm: 1.25 ± 0.05 m/s; 18 cm: 1.12 ± 0.03 m/s	Spatiotemporal, kinematics, kinetics and muscle function.

HHS High-heeled shoes, *LHS* low-heeled shoes, *BF* barefoot.
