# Supplementary material for: Effects of high-heeled shoes on lower extremity biomechanics and balance in females: a systematic review and meta-analysis
Source: BMC Public Health. 2023 Apr 20;23:726. doi: 10.1186/s12889-023-15641-8 (PMC10120101; doi:10.1186/s12889-023-15641-8)
Supplement: Supplementary file 5 — Additional file 5. [file 12889_2023_15641_MOESM5_ESM.pdf]

Additional file 5

The meta-analyses of spatiotemporal, kinematics, kinetics, plantar pressure and balance outcomes

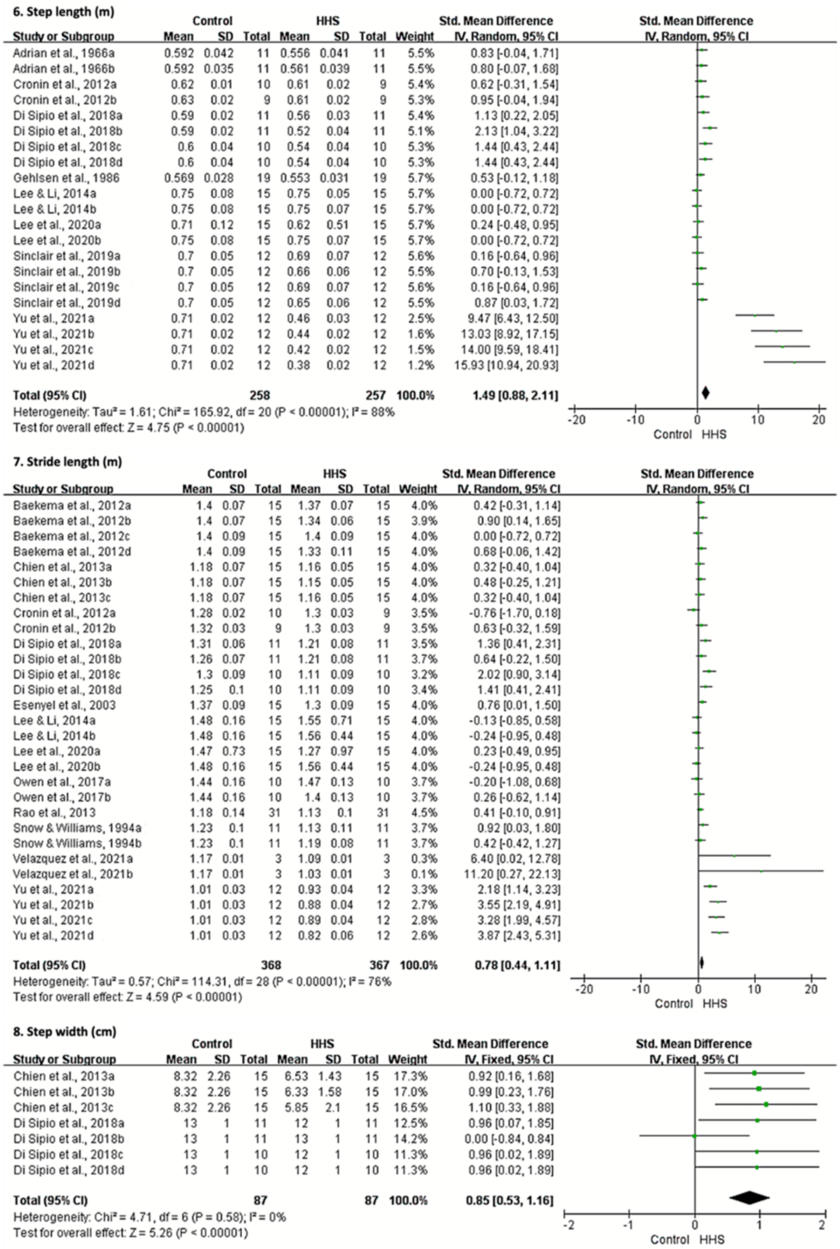

6. Step length (m)

Study or Subgroup	Control Mean	Control SD	Control Total	HHS Mean	HHS SD	HHS Total	Weight	Std. Mean Difference IV, Random, 95% CI
Adrian et al., 1966a	0.592	0.042	11	0.556	0.041	11	5.5%	0.83 [-0.04, 1.71]
Adrian et al., 1966b	0.592	0.035	11	0.561	0.039	11	5.5%	0.80 [-0.07, 1.68]
Cronin et al., 2012a	0.62	0.01	10	0.61	0.02	9	5.4%	0.62 [-0.31, 1.54]
Cronin et al., 2012b	0.63	0.02	9	0.61	0.02	9	5.3%	0.95 [-0.04, 1.94]
Di Sipio et al., 2018a	0.59	0.02	11	0.56	0.03	11	5.4%	1.13 [0.22, 2.05]
Di Sipio et al., 2018b	0.59	0.02	11	0.52	0.04	11	5.1%	2.13 [1.04, 3.22]
Di Sipio et al., 2018c	0.6	0.04	10	0.54	0.04	10	5.3%	1.44 [0.43, 2.44]
Di Sipio et al., 2018d	0.6	0.04	10	0.54	0.04	10	5.3%	1.44 [0.43, 2.44]
Gehlsen et al., 1986	0.569	0.028	19	0.553	0.031	19	5.7%	0.53 [-0.12, 1.18]
Lee & Li, 2014a	0.75	0.08	15	0.75	0.05	15	5.7%	0.00 [-0.72, 0.72]
Lee & Li, 2014b	0.75	0.08	15	0.75	0.07	15	5.7%	0.00 [-0.72, 0.72]
Lee et al., 2020a	0.71	0.12	15	0.62	0.51	15	5.7%	0.24 [-0.48, 0.95]
Lee et al., 2020b	0.75	0.08	15	0.75	0.07	15	5.7%	0.00 [-0.72, 0.72]
Sinclair et al., 2019a	0.7	0.05	12	0.69	0.07	12	5.6%	0.16 [-0.64, 0.96]
Sinclair et al., 2019b	0.7	0.05	12	0.66	0.06	12	5.5%	0.70 [-0.13, 1.53]
Sinclair et al., 2019c	0.7	0.05	12	0.69	0.07	12	5.6%	0.16 [-0.64, 0.96]
Sinclair et al., 2019d	0.7	0.05	12	0.65	0.06	12	5.5%	0.87 [0.03, 1.72]
Yu et al., 2021a	0.71	0.02	12	0.46	0.03	12	2.5%	9.47 [6.43, 12.50]
Yu et al., 2021b	0.71	0.02	12	0.44	0.02	12	1.6%	13.03 [8.92, 17.15]
Yu et al., 2021c	0.71	0.02	12	0.42	0.02	12	1.5%	14.00 [9.59, 18.41]
Yu et al., 2021d	0.71	0.02	12	0.38	0.02	12	1.2%	15.93 [10.94, 20.93]
**Total (95% CI)**			**258**			**257**	**100.0%**	**1.49 [0.88, 2.11]**

Heterogeneity: $Tau^2 = 1.61$; $Chi^2 = 165.92$, df = 20 ($P < 0.00001$); $I^2 = 88\%$
Test for overall effect: $Z = 4.75$ ($P < 0.00001$)

7. Stride length (m)

Study or Subgroup	Control Mean	Control SD	Control Total	HHS Mean	HHS SD	HHS Total	Weight	Std. Mean Difference IV, Random, 95% CI
Baekema et al., 2012a	1.4	0.07	15	1.37	0.07	15	4.0%	0.42 [-0.31, 1.14]
Baekema et al., 2012b	1.4	0.07	15	1.34	0.06	15	3.9%	0.90 [0.14, 1.65]
Baekema et al., 2012c	1.4	0.09	15	1.4	0.09	15	4.0%	0.00 [-0.72, 0.72]
Baekema et al., 2012d	1.4	0.09	15	1.33	0.11	15	4.0%	0.68 [-0.06, 1.42]
Chien et al., 2013a	1.18	0.07	15	1.16	0.05	15	4.0%	0.32 [-0.40, 1.04]
Chien et al., 2013b	1.18	0.07	15	1.15	0.05	15	4.0%	0.48 [-0.25, 1.21]
Chien et al., 2013c	1.18	0.07	15	1.16	0.05	15	4.0%	0.32 [-0.40, 1.04]
Cronin et al., 2012a	1.28	0.02	10	1.3	0.03	9	3.5%	-0.76 [-1.70, 0.18]
Cronin et al., 2012b	1.32	0.03	9	1.3	0.03	9	3.5%	0.63 [-0.32, 1.59]
Di Sipio et al., 2018a	1.31	0.06	11	1.21	0.08	11	3.5%	1.36 [0.41, 2.31]
Di Sipio et al., 2018b	1.26	0.07	11	1.21	0.08	11	3.7%	0.64 [-0.22, 1.50]
Di Sipio et al., 2018c	1.3	0.09	10	1.11	0.09	10	3.2%	2.02 [0.90, 3.14]
Di Sipio et al., 2018d	1.25	0.1	10	1.11	0.09	10	3.4%	1.41 [0.41, 2.41]
Esenyel et al., 2003	1.37	0.09	15	1.3	0.09	15	4.0%	0.76 [0.01, 1.50]
Lee & Li, 2014a	1.48	0.16	15	1.55	0.71	15	4.0%	-0.13 [-0.85, 0.58]
Lee & Li, 2014b	1.48	0.16	15	1.56	0.44	15	4.0%	-0.24 [-0.95, 0.48]
Lee et al., 2020a	1.47	0.73	15	1.27	0.97	15	4.0%	0.23 [-0.49, 0.95]
Lee et al., 2020b	1.48	0.16	15	1.56	0.44	15	4.0%	-0.24 [-0.95, 0.48]
Owen et al., 2017a	1.44	0.16	10	1.47	0.13	10	3.7%	-0.20 [-1.08, 0.68]
Owen et al., 2017b	1.44	0.16	10	1.4	0.13	10	3.7%	0.26 [-0.62, 1.14]
Rao et al., 2013	1.18	0.14	31	1.13	0.1	31	4.5%	0.41 [-0.10, 0.91]
Snow & Williams, 1994a	1.23	0.1	11	1.13	0.11	11	3.7%	0.92 [0.03, 1.80]
Snow & Williams, 1994b	1.23	0.1	11	1.19	0.08	11	3.7%	0.42 [-0.42, 1.27]
Velazquez et al., 2021a	1.17	0.01	3	1.09	0.01	3	0.3%	6.40 [0.02, 12.78]
Velazquez et al., 2021b	1.17	0.01	3	1.03	0.01	3	0.1%	11.20 [0.27, 22.13]
Yu et al., 2021a	1.01	0.03	12	0.93	0.04	12	3.3%	2.18 [1.14, 3.23]
Yu et al., 2021b	1.01	0.03	12	0.88	0.04	12	2.7%	3.55 [2.19, 4.91]
Yu et al., 2021c	1.01	0.03	12	0.89	0.04	12	2.8%	3.28 [1.99, 4.57]
Yu et al., 2021d	1.01	0.03	12	0.82	0.06	12	2.6%	3.87 [2.43, 5.31]
**Total (95% CI)**			**368**			**367**	**100.0%**	**0.78 [0.44, 1.11]**

Heterogeneity: $Tau^2 = 0.57$; $Chi^2 = 114.31$, df = 28 ($P < 0.00001$); $I^2 = 76\%$
Test for overall effect: $Z = 4.59$ ($P < 0.00001$)

8. Step width (cm)

Study or Subgroup	Control Mean	Control SD	Control Total	HHS Mean	HHS SD	HHS Total	Weight	Std. Mean Difference IV, Fixed, 95% CI
Chien et al., 2013a	8.32	2.26	15	6.53	1.43	15	17.3%	0.92 [0.16, 1.68]
Chien et al., 2013b	8.32	2.26	15	6.33	1.58	15	17.0%	0.99 [0.23, 1.76]
Chien et al., 2013c	8.32	2.26	15	5.85	2.1	15	16.5%	1.10 [0.33, 1.88]
Di Sipio et al., 2018a	13	1	11	12	1	11	12.5%	0.96 [0.07, 1.85]
Di Sipio et al., 2018b	13	1	11	13	1	11	14.2%	0.00 [-0.84, 0.84]
Di Sipio et al., 2018c	13	1	10	12	1	10	11.3%	0.96 [0.02, 1.89]
Di Sipio et al., 2018d	13	1	10	12	1	10	11.3%	0.96 [0.02, 1.89]
**Total (95% CI)**			**87**			**87**	**100.0%**	**0.85 [0.53, 1.16]**

Heterogeneity: $Chi^2 = 4.71$, df = 6 ($P = 0.58$); $I^2 = 0\%$
Test for overall effect: $Z = 5.26$ ($P < 0.00001$)

Online supplemental appendix 3 Figure 1 Meta-analysis of spatial characteristics during walking in high-heeled shoes compared with flat shoes or barefoot. *IV* inverse variance, *CI* confidence interval, *HHS* high-heeled shoes.

9. Step frequency (steps/min)

Study or Subgroup	Control Mean	Control SD	Control Total	HHS Mean	HHS SD	HHS Total	Weight	Std. Mean Difference IV, Random, 95% CI
Adrian et al., 1966	105.9	6.25	11	103.6	4.77	11	5.1%	0.40 [-0.45, 1.24]
Chien et al., 2013a	117.36	7.38	15	112.63	7.91	15	5.8%	0.60 [-0.13, 1.34]
Chien et al., 2013b	117.36	7.38	15	114.09	7.39	15	5.8%	0.43 [-0.29, 1.16]
Chien et al., 2013c	117.36	7.38	15	111.4	7.81	15	5.7%	0.76 [0.02, 1.51]
Di Sipio et al., 2018a	100.44	29.9	11	110.09	6.05	11	5.1%	-0.43 [-1.28, 0.42]
Di Sipio et al., 2018b	100.44	29.9	11	106.56	7.06	11	5.2%	-0.27 [-1.11, 0.57]
Di Sipio et al., 2018c	108.38	4.62	10	106.56	6.23	10	4.9%	0.32 [-0.57, 1.20]
Di Sipio et al., 2018d	108.38	4.62	10	107.64	6.32	10	5.0%	0.13 [-0.75, 1.01]
Esenyel et al., 2003	113.4	10.6	15	112	10.2	15	5.9%	0.13 [-0.59, 0.85]
Jandova et al., 2019a	92.468	8.392	30	99.004	6.597	30	7.0%	-0.85 [-1.38, -0.32]
Jandova et al., 2019b	66.303	6.547	30	70.878	7.352	30	7.1%	-0.65 [-1.17, -0.13]
Lee & Li, 2014a	125	14.1	15	118.8	17.7	15	5.8%	0.38 [-0.35, 1.10]
Lee & Li, 2014b	125	14.1	15	116.5	19.7	15	5.8%	0.48 [-0.24, 1.21]
Wang et al., 2001	104.88	6.4	10	109.75	7.36	10	4.8%	-0.68 [-1.58, 0.23]
Yu et al., 2021a	100.8	3.6	12	102	0.6	12	5.3%	-0.45 [-1.26, 0.36]
Yu et al., 2021b	100.8	3.6	12	100.2	4.2	12	5.4%	0.15 [-0.65, 0.95]
Yu et al., 2021c	100.8	3.6	12	99	3	12	5.3%	0.52 [-0.29, 1.34]
Yu et al., 2021d	100.8	3.6	12	95.4	3.6	12	4.8%	1.45 [0.53, 2.37]
**Total (95% CI)**			**261**			**261**	**100.0%**	**0.11 [-0.18, 0.40]**

Heterogeneity: Tau² = 0.23; Chi² = 44.06, df = 17 (P = 0.0003); I² = 61%
Test for overall effect: Z = 0.75 (P = 0.45)

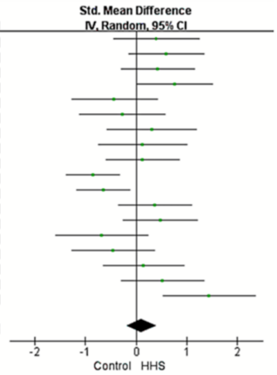

10. Walking velocity (m/s)

Study or Subgroup	Control Mean	Control SD	Control Total	HHS Mean	HHS SD	HHS Total	Weight	Std. Mean Difference IV, Random, 95% CI
Baekema et al., 2012a	1.31	0.01	15	1.3	0.01	15	3.7%	0.97 [0.21, 1.74]
Baekema et al., 2012b	1.31	0.01	15	1.3	0.01	15	3.7%	0.97 [0.21, 1.74]
Baekema et al., 2012c	1.35	0.1	15	1.35	0.15	15	3.9%	0.00 [-0.72, 0.72]
Baekema et al., 2012d	1.35	0.1	15	1.28	0.15	15	3.8%	0.53 [-0.20, 1.26]
Chien et al., 2013a	1.15	0.11	15	1.09	0.09	15	3.8%	0.58 [-0.15, 1.31]
Chien et al., 2013b	1.15	0.11	15	1.09	0.09	15	3.8%	0.58 [-0.15, 1.31]
Chien et al., 2013c	1.15	0.11	15	1.07	0.09	15	3.8%	0.77 [0.03, 1.52]
Cronin et al., 2012a	1.19	0.05	10	1.2	0.03	9	3.3%	-0.23 [-1.13, 0.68]
Cronin et al., 2012b	1.22	0.04	9	1.2	0.03	9	3.2%	0.54 [-0.41, 1.48]
Di Sipio et al., 2018a	1.18	0.1	11	1.14	0.1	11	3.5%	0.38 [-0.46, 1.23]
Di Sipio et al., 2018b	1.18	0.1	11	1.06	0.12	11	3.3%	1.05 [0.14, 1.95]
Di Sipio et al., 2018c	1.16	0.05	10	1.09	0.09	10	3.3%	0.92 [-0.01, 1.85]
Di Sipio et al., 2018d	1.16	0.05	10	1.1	0.09	10	3.3%	0.79 [-0.13, 1.71]
Esenyel et al., 2003	1.29	0.15	15	1.22	0.12	15	3.8%	0.50 [-0.23, 1.23]
McBride et al., 1991	1.34	0.18	11	1.31	0.18	11	3.5%	0.16 [-0.68, 1.00]
Mika et al., 2012a	1.36	0.6	31	1.3	0.6	31	4.5%	0.10 [-0.40, 0.60]
Mika et al., 2012b	1.36	0.6	31	1.26	0.7	31	4.5%	0.15 [-0.35, 0.65]
Mika et al., 2012c	1.33	0.5	15	1.24	0.6	15	3.9%	0.16 [-0.56, 0.88]
Mika et al., 2012d	1.33	0.5	15	1.21	0.6	15	3.9%	0.21 [-0.51, 0.93]
Owen et al., 2017a	1.4	0.22	10	1.41	0.15	10	3.4%	-0.05 [-0.93, 0.83]
Owen et al., 2017b	1.4	0.22	10	1.37	0.18	10	3.4%	0.14 [-0.73, 1.02]
Rao et al., 2013	1.03	0.14	31	0.99	0.12	31	4.4%	0.30 [-0.20, 0.80]
Titchenal et al., 2015a	1.41	0.14	14	1.28	0.1	14	3.6%	1.04 [0.24, 1.83]
Titchenal et al., 2015b	1.41	0.14	14	1.17	0.13	14	3.4%	1.72 [0.84, 2.61]
Velazquez et al., 2021a	1.01	0.01	3	0.99	0.01	3	1.2%	1.60 [-0.62, 3.82]
Velazquez et al., 2021b	1.01	0.01	3	0.94	0.01	3	0.2%	5.60 [-0.04, 11.24]
Yu et al., 2021a	1.48	0.08	12	1.38	0.08	12	3.4%	1.21 [0.32, 2.09]
Yu et al., 2021b	1.48	0.08	12	1.3	0.03	12	2.6%	2.88 [1.68, 4.07]
Yu et al., 2021c	1.48	0.08	12	1.25	0.05	12	2.4%	3.33 [2.02, 4.63]
Yu et al., 2021d	1.48	0.08	12	1.12	0.03	12	1.4%	5.75 [3.80, 7.71]
**Total (95% CI)**			**417**			**416**	**100.0%**	**0.75 [0.48, 1.03]**

Heterogeneity: Tau² = 0.38; Chi² = 95.39, df = 29 (P < 0.00001); I² = 70%
Test for overall effect: Z = 5.37 (P < 0.00001)

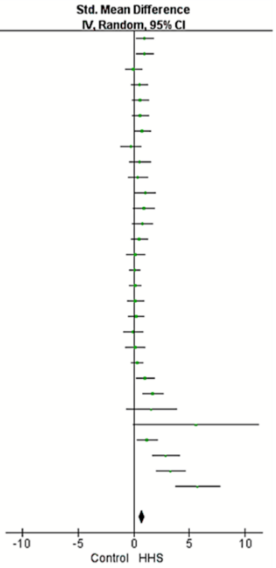

Online supplemental appendix 3 Figure 2 Meta-analysis of spatiotemporal characteristics during walking in high-heeled shoes compared with flat shoes or barefoot. *IV* inverse variance, *CI* confidence interval, *HHS* high-heeled shoes.

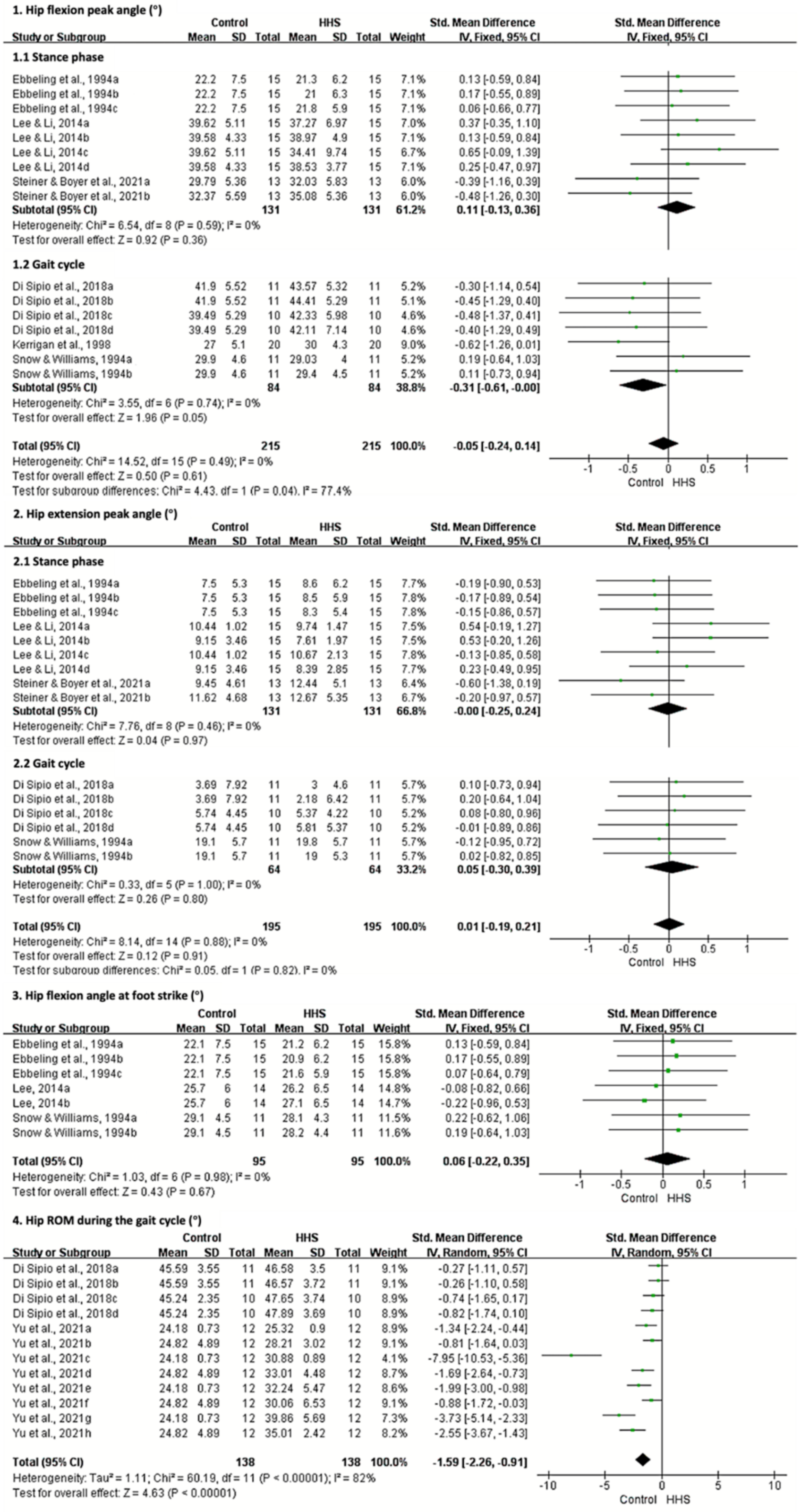

1. Hip flexion peak angle (°)

Study or Subgroup	Control Mean	Control SD	Control Total	HHS Mean	HHS SD	HHS Total	Weight	Std. Mean Difference IV, Fixed, 95% CI
**1.1 Stance phase**								
Ebbeling et al., 1994a	22.2	7.5	15	21.3	6.2	15	7.1%	0.13 [-0.59, 0.84]
Ebbeling et al., 1994b	22.2	7.5	15	21	6.3	15	7.1%	0.17 [-0.55, 0.89]
Ebbeling et al., 1994c	22.2	7.5	15	21.8	5.9	15	7.1%	0.06 [-0.66, 0.77]
Lee & Li, 2014a	39.62	5.11	15	37.27	6.97	15	7.0%	0.37 [-0.35, 1.10]
Lee & Li, 2014b	39.58	4.33	15	38.97	4.9	15	7.1%	0.13 [-0.59, 0.84]
Lee & Li, 2014c	39.62	5.11	15	34.41	9.74	15	6.7%	0.65 [-0.09, 1.39]
Lee & Li, 2014d	39.58	4.33	15	38.53	3.77	15	7.1%	0.25 [-0.47, 0.97]
Steiner & Boyer et al., 2021a	29.79	5.36	13	32.03	5.83	13	6.0%	-0.39 [-1.16, 0.39]
Steiner & Boyer et al., 2021b	32.37	5.59	13	35.08	5.36	13	6.0%	-0.48 [-1.26, 0.30]
**Subtotal (95% CI)**			**131**			**131**	**61.2%**	**0.11 [-0.13, 0.36]**
Heterogeneity: Chi² = 6.54, df = 8 (P = 0.59); I² = 0%								
Test for overall effect: Z = 0.92 (P = 0.36)								
**1.2 Gait cycle**								
Di Sipio et al., 2018a	41.9	5.52	11	43.57	5.32	11	5.2%	-0.30 [-1.14, 0.54]
Di Sipio et al., 2018b	41.9	5.52	11	44.41	5.29	11	5.1%	-0.45 [-1.29, 0.40]
Di Sipio et al., 2018c	39.49	5.29	10	42.33	5.98	10	4.6%	-0.48 [-1.37, 0.41]
Di Sipio et al., 2018d	39.49	5.29	10	42.11	7.14	10	4.6%	-0.40 [-1.29, 0.49]
Kerrigan et al., 1998	27	5.1	20	30	4.3	20	9.0%	-0.62 [-1.26, 0.01]
Snow & Williams, 1994a	29.9	4.6	11	29.03	4	11	5.2%	0.19 [-0.64, 1.03]
Snow & Williams, 1994b	29.9	4.6	11	29.4	4.5	11	5.2%	0.11 [-0.73, 0.94]
**Subtotal (95% CI)**			**84**			**84**	**38.8%**	**-0.31 [-0.61, -0.00]**
Heterogeneity: Chi² = 3.55, df = 6 (P = 0.74); I² = 0%								
Test for overall effect: Z = 1.96 (P = 0.05)								
**Total (95% CI)**			**215**			**215**	**100.0%**	**-0.05 [-0.24, 0.14]**
Heterogeneity: Chi² = 14.52, df = 15 (P = 0.49); I² = 0%								
Test for overall effect: Z = 0.50 (P = 0.61)								
Test for subgroup differences: Chi² = 4.43, df = 1 (P = 0.04), I² = 77.4%								

2. Hip extension peak angle (°)

Study or Subgroup	Control Mean	Control SD	Control Total	HHS Mean	HHS SD	HHS Total	Weight	Std. Mean Difference IV, Fixed, 95% CI
**2.1 Stance phase**								
Ebbeling et al., 1994a	7.5	5.3	15	8.6	6.2	15	7.7%	-0.19 [-0.90, 0.53]
Ebbeling et al., 1994b	7.5	5.3	15	8.5	5.9	15	7.8%	-0.17 [-0.89, 0.54]
Ebbeling et al., 1994c	7.5	5.3	15	8.3	5.4	15	7.8%	-0.15 [-0.86, 0.57]
Lee & Li, 2014a	10.44	1.02	15	9.74	1.47	15	7.5%	0.54 [-0.19, 1.27]
Lee & Li, 2014b	9.15	3.46	15	7.61	1.97	15	7.5%	0.53 [-0.20, 1.26]
Lee & Li, 2014c	10.44	1.02	15	10.67	2.13	15	7.8%	-0.13 [-0.85, 0.58]
Lee & Li, 2014d	9.15	3.46	15	8.39	2.85	15	7.7%	0.23 [-0.49, 0.95]
Steiner & Boyer et al., 2021a	9.45	4.61	13	12.44	5.1	13	6.4%	-0.60 [-1.38, 0.19]
Steiner & Boyer et al., 2021b	11.62	4.68	13	12.67	5.35	13	6.7%	-0.20 [-0.97, 0.57]
**Subtotal (95% CI)**			**131**			**131**	**66.8%**	**-0.00 [-0.25, 0.24]**
Heterogeneity: Chi² = 7.76, df = 8 (P = 0.46); I² = 0%								
Test for overall effect: Z = 0.04 (P = 0.97)								
**2.2 Gait cycle**								
Di Sipio et al., 2018a	3.69	7.92	11	3	4.6	11	5.7%	0.10 [-0.73, 0.94]
Di Sipio et al., 2018b	3.69	7.92	11	2.18	6.42	11	5.7%	0.20 [-0.64, 1.04]
Di Sipio et al., 2018c	5.74	4.45	10	5.37	4.22	10	5.2%	0.08 [-0.80, 0.96]
Di Sipio et al., 2018d	5.74	4.45	10	5.81	5.37	10	5.2%	-0.01 [-0.89, 0.86]
Snow & Williams, 1994a	19.1	5.7	11	19.8	5.7	11	5.7%	-0.12 [-0.95, 0.72]
Snow & Williams, 1994b	19.1	5.7	11	19	5.3	11	5.7%	0.02 [-0.82, 0.85]
**Subtotal (95% CI)**			**64**			**64**	**33.2%**	**0.05 [-0.30, 0.39]**
Heterogeneity: Chi² = 0.33, df = 5 (P = 1.00); I² = 0%								
Test for overall effect: Z = 0.26 (P = 0.80)								
**Total (95% CI)**			**195**			**195**	**100.0%**	**0.01 [-0.19, 0.21]**
Heterogeneity: Chi² = 8.14, df = 14 (P = 0.88); I² = 0%								
Test for overall effect: Z = 0.12 (P = 0.91)								
Test for subgroup differences: Chi² = 0.05, df = 1 (P = 0.82), I² = 0%								

3. Hip flexion angle at foot strike (°)

Study or Subgroup	Control Mean	Control SD	Control Total	HHS Mean	HHS SD	HHS Total	Weight	Std. Mean Difference IV, Fixed, 95% CI
Ebbeling et al., 1994a	22.1	7.5	15	21.2	6.2	15	15.8%	0.13 [-0.59, 0.84]
Ebbeling et al., 1994b	22.1	7.5	15	20.9	6.2	15	15.8%	0.17 [-0.55, 0.89]
Ebbeling et al., 1994c	22.1	7.5	15	21.6	5.9	15	15.8%	0.07 [-0.64, 0.79]
Lee, 2014a	25.7	6	14	26.2	6.5	14	14.8%	-0.08 [-0.82, 0.66]
Lee, 2014b	25.7	6	14	27.1	6.5	14	14.7%	-0.22 [-0.96, 0.53]
Snow & Williams, 1994a	29.1	4.5	11	28.1	4.3	11	11.5%	0.22 [-0.62, 1.06]
Snow & Williams, 1994b	29.1	4.5	11	28.2	4.4	11	11.6%	0.19 [-0.64, 1.03]
**Total (95% CI)**			**95**			**95**	**100.0%**	**0.06 [-0.22, 0.35]**
Heterogeneity: Chi² = 1.03, df = 6 (P = 0.98); I² = 0%								
Test for overall effect: Z = 0.43 (P = 0.67)								

4. Hip ROM during the gait cycle (°)

Study or Subgroup	Control Mean	Control SD	Control Total	HHS Mean	HHS SD	HHS Total	Weight	Std. Mean Difference IV, Random, 95% CI
Di Sipio et al., 2018a	45.59	3.55	11	46.58	3.5	11	9.1%	-0.27 [-1.11, 0.57]
Di Sipio et al., 2018b	45.59	3.55	11	46.57	3.72	11	9.1%	-0.26 [-1.10, 0.58]
Di Sipio et al., 2018c	45.24	2.35	10	47.65	3.74	10	8.9%	-0.74 [-1.65, 0.17]
Di Sipio et al., 2018d	45.24	2.35	10	47.89	3.69	10	8.9%	-0.82 [-1.74, 0.10]
Yu et al., 2021a	24.18	0.73	12	25.32	0.9	12	8.9%	-1.34 [-2.24, -0.44]
Yu et al., 2021b	24.82	4.89	12	28.21	3.02	12	9.1%	-0.81 [-1.64, 0.03]
Yu et al., 2021c	24.18	0.73	12	30.88	0.89	12	4.1%	-7.95 [-10.53, -5.36]
Yu et al., 2021d	24.82	4.89	12	33.01	4.48	12	8.7%	-1.69 [-2.64, -0.73]
Yu et al., 2021e	24.18	0.73	12	32.24	5.47	12	8.6%	-1.99 [-3.00, -0.98]
Yu et al., 2021f	24.82	4.89	12	30.06	6.53	12	9.1%	-0.88 [-1.72, -0.03]
Yu et al., 2021g	24.18	0.73	12	39.86	5.69	12	7.3%	-3.73 [-5.14, -2.33]
Yu et al., 2021h	24.82	4.89	12	35.01	2.42	12	8.2%	-2.55 [-3.67, -1.43]
**Total (95% CI)**			**138**			**138**	**100.0%**	**-1.59 [-2.26, -0.91]**
Heterogeneity: Tau² = 1.11; Chi² = 60.19, df = 11 (P < 0.00001); I² = 82%								
Test for overall effect: Z = 4.63 (P < 0.00001)								

Online supplemental appendix 3 Figure 3 Meta-analysis of hip kinematics during walking in high-heeled shoes compared with flat shoes or barefoot. *IV* inverse variance, *CI* confidence interval, *HHS* high-heeled shoes, *ROM* range of motion.

1. Knee flexion peak angle (°)

Study or Subgroup	Control Mean	Control SD	Control Total	HHS Mean	HHS SD	HHS Total	Weight	Std. Mean Difference IV, Random, 95% CI
**1.1 Stance phase**								
Ebbeling et al., 1994a	21.1	6	15	22.6	6.9	15	6.3%	-0.23 [-0.94, 0.49]
Ebbeling et al., 1994b	21.1	6	15	23.2	7.4	15	6.3%	-0.30 [-1.02, 0.42]
Ebbeling et al., 1994c	21.1	6	15	24.9	7.1	15	6.2%	-0.56 [-1.29, 0.17]
Kerrigan et al., 1998	22.6	4.2	20	19.7	4.6	20	6.6%	0.65 [0.01, 1.28]
Snow & Williams, 1994a	26.6	6	11	24.3	6.8	11	5.8%	0.35 [-0.50, 1.19]
Snow & Williams, 1994b	26.6	6	11	23.1	5.9	11	5.7%	0.57 [-0.29, 1.42]
**Subtotal (95% CI)**			**87**			**87**	**36.9%**	**0.07 [-0.35, 0.49]**
Heterogeneity: Tau² = 0.13; Chi² = 9.38, df = 5 (P = 0.09); I² = 47%								
Test for overall effect: Z = 0.33 (P = 0.74)								
**1.2 Gait cycle**								
Di Sipio et al., 2018a	66.31	4.88	11	59.59	6.23	11	5.5%	1.16 [0.24, 2.07]
Di Sipio et al., 2018b	66.31	4.88	11	57.02	6.14	11	5.2%	1.61 [0.62, 2.60]
Di Sipio et al., 2018c	68	3.61	10	60.97	4.64	10	5.0%	1.62 [0.58, 2.66]
Di Sipio et al., 2018d	68	3.61	10	59.38	3.51	10	4.5%	2.32 [1.13, 3.51]
Kerrigan et al., 1998	60.5	4.3	20	63.5	4.5	20	6.6%	-0.67 [-1.31, -0.03]
Lee & Li, 2014a	64.17	9.33	15	60.73	8.66	15	6.3%	0.37 [-0.35, 1.09]
Lee & Li, 2014b	62.42	10.22	15	63.18	9.79	15	6.3%	-0.07 [-0.79, 0.64]
Lee & Li, 2014c	64.17	9.33	15	63.94	8.15	15	6.3%	0.03 [-0.69, 0.74]
Lee & Li, 2014d	62.42	10.22	15	69.28	7.86	15	6.2%	-0.73 [-1.47, 0.01]
Snow & Williams, 1994a	65.1	5.1	11	62	5.1	11	5.7%	0.58 [-0.27, 1.44]
Snow & Williams, 1994b	65.1	5.1	11	58	5.8	11	5.4%	1.25 [0.32, 2.18]
**Subtotal (95% CI)**			**144**			**144**	**63.1%**	**0.61 [0.05, 1.16]**
Heterogeneity: Tau² = 0.70; Chi² = 49.43, df = 10 (P < 0.00001); I² = 80%								
Test for overall effect: Z = 2.13 (P = 0.03)								
**Total (95% CI)**			**231**			**231**	**100.0%**	**0.39 [0.02, 0.77]**
Heterogeneity: Tau² = 0.46; Chi² = 61.06, df = 16 (P < 0.00001); I² = 74%								
Test for overall effect: Z = 2.04 (P = 0.04)								
Test for subgroup differences: Chi² = 2.29, df = 1 (P = 0.13), I² = 56.3%								

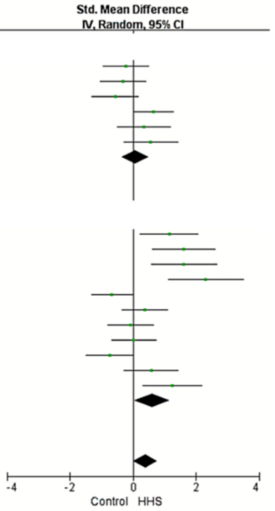

2. Knee extension peak angle (°)

Study or Subgroup	Control Mean	Control SD	Control Total	HHS Mean	HHS SD	HHS Total	Weight	Std. Mean Difference IV, Random, 95% CI
**2.1 Stance phase**								
Ebbeling et al., 1994a	8.8	4.3	15	10.3	4.5	15	8.1%	-0.33 [-1.05, 0.39]
Ebbeling et al., 1994b	8.8	4.3	15	10.1	4.2	15	8.1%	-0.30 [-1.02, 0.42]
Ebbeling et al., 1994c	8.8	4.3	15	10.2	4.1	15	8.1%	-0.32 [-1.05, 0.40]
Lee & Li, 2014a	3.87	2.46	15	2.76	2.01	15	8.1%	0.48 [-0.25, 1.21]
Lee & Li, 2014b	4.29	1.97	15	3.33	0.09	15	8.1%	0.67 [-0.07, 1.41]
Lee & Li, 2014c	3.87	2.46	15	-0.67	1.03	15	7.2%	2.34 [1.39, 3.30]
Lee & Li, 2014d	4.29	1.97	15	0.32	0.09	15	6.8%	2.77 [1.73, 3.81]
**Subtotal (95% CI)**			**105**			**105**	**54.5%**	**0.71 [-0.13, 1.55]**
Heterogeneity: Tau² = 1.11; Chi² = 47.60, df = 6 (P < 0.00001); I² = 87%								
Test for overall effect: Z = 1.66 (P = 0.10)								
**2.2 Gait cycle**								
Di Sipio et al., 2018a	4.2	4.91	11	4.89	4.7	11	7.7%	-0.14 [-0.98, 0.70]
Di Sipio et al., 2018b	4.2	4.91	11	7.49	3.86	11	7.5%	-0.72 [-1.58, 0.15]
Di Sipio et al., 2018c	6.71	3.57	10	6.28	4.28	10	7.5%	0.10 [-0.77, 0.98]
Di Sipio et al., 2018d	6.71	3.57	10	6.23	4.17	10	7.5%	0.12 [-0.76, 1.00]
Snow & Williams, 1994a	-2.1	5.1	11	-2.9	5.6	11	7.7%	0.14 [-0.69, 0.98]
Snow & Williams, 1994b	-2.1	5.1	11	-2.7	5.3	11	7.7%	0.11 [-0.73, 0.95]
**Subtotal (95% CI)**			**64**			**64**	**45.5%**	**-0.06 [-0.41, 0.29]**
Heterogeneity: Tau² = 0.00; Chi² = 2.92, df = 5 (P = 0.71); I² = 0%								
Test for overall effect: Z = 0.34 (P = 0.73)								
**Total (95% CI)**			**169**			**169**	**100.0%**	**0.34 [-0.14, 0.83]**
Heterogeneity: Tau² = 0.63; Chi² = 55.72, df = 12 (P < 0.00001); I² = 78%								
Test for overall effect: Z = 1.38 (P = 0.17)								
Test for subgroup differences: Chi² = 2.77, df = 1 (P = 0.10), I² = 63.9%								

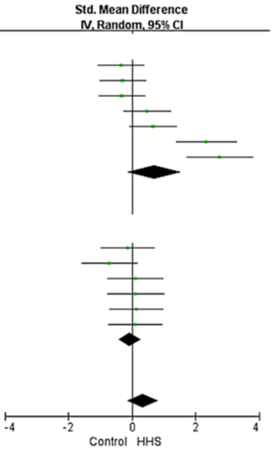

3. Knee flexion angle at foot strike (°)

Study or Subgroup	Control Mean	Control SD	Control Total	HHS Mean	HHS SD	HHS Total	Weight	Std. Mean Difference IV, Fixed, 95% CI
Chhoeum et al., 2020a	8.5	1.8	7	12.3	6.8	7	3.5%	-0.72 [-1.81, 0.38]
Chhoeum et al., 2020b	8.5	1.8	7	15.3	6.2	7	2.9%	-1.39 [-2.61, -0.18]
Chhoeum et al., 2020c	8.5	1.8	7	13.2	6.3	7	3.3%	-0.95 [-2.08, 0.18]
Chhoeum et al., 2020d	15.6	8	7	22.3	7.3	7	3.4%	-0.82 [-1.93, 0.29]
Chhoeum et al., 2020e	15.6	8	7	26.1	7.1	7	3.0%	-1.30 [-2.49, -0.11]
Chhoeum et al., 2020f	15.6	8	7	25.4	6.3	7	3.0%	-1.27 [-2.46, -0.09]
Ebbeling et al., 1994a	9.5	4.7	15	11.4	5	15	8.0%	-0.38 [-1.10, 0.34]
Ebbeling et al., 1994b	9.5	4.7	15	11.14	4.7	15	8.1%	-0.34 [-1.06, 0.38]
Ebbeling et al., 1994c	9.5	4.7	15	13.1	4.7	15	7.6%	-0.75 [-1.49, -0.00]
Gollnick et al., 1964	170	7.8	5	169	9.8	5	2.7%	0.10 [-1.14, 1.34]
Lee, 2014a	-14.3	6.8	14	-14.7	7.8	14	7.7%	0.05 [-0.69, 0.79]
Lee, 2014b	-14.3	6.8	14	-15.6	6.1	14	7.6%	0.20 [-0.55, 0.94]
Snow & Williams, 1994a	20.2	7.8	11	20.4	6.2	11	6.0%	-0.03 [-0.86, 0.81]
Snow & Williams, 1994b	20.2	7.8	11	18.7	5.1	11	6.0%	0.22 [-0.62, 1.06]
Steiner & Boyer et al., 2021a	-0.03	3.2	13	-0.34	5.9	13	7.1%	0.06 [-0.71, 0.83]
Steiner & Boyer et al., 2021b	2.3	3.36	13	2.16	6.09	13	7.1%	0.03 [-0.74, 0.80]
Titchenal et al., 2015a	-3.2	3.15	14	-0.15	2.61	14	6.6%	-1.02 [-1.82, -0.23]
Titchenal et al., 2015b	-3.2	3.15	14	1.41	4.19	14	6.3%	-1.21 [-2.02, -0.39]
**Total (95% CI)**			**196**			**196**	**100.0%**	**-0.42 [-0.63, -0.22]**
Heterogeneity: Chi² = 25.56, df = 17 (P = 0.08); I² = 33%								
Test for overall effect: Z = 4.02 (P < 0.0001)								

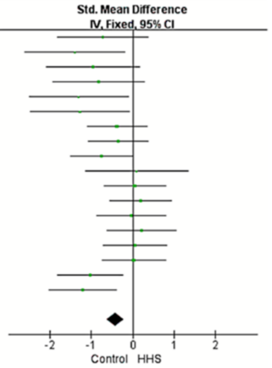

4. Knee flexion angle at toe-off (°)

Study or Subgroup	Control Mean	Control SD	Control Total	HHS Mean	HHS SD	HHS Total	Weight	Std. Mean Difference IV, Fixed, 95% CI
Chhoeum et al., 2020a	61	8.4	7	52.9	9.7	7	7.7%	0.84 [-0.27, 1.95]
Chhoeum et al., 2020b	61	8.4	7	54.9	10.3	7	8.1%	0.61 [-0.47, 1.69]
Chhoeum et al., 2020c	61	8.4	7	52.9	9.4	7	7.7%	0.85 [-0.26, 1.96]
Chhoeum et al., 2020d	70.9	3.7	7	65	6.1	7	7.2%	1.09 [-0.06, 2.25]
Chhoeum et al., 2020e	70.9	3.7	7	64.8	3.9	7	6.2%	1.50 [0.27, 2.74]
Chhoeum et al., 2020f	70.9	3.7	7	64.1	2.6	7	5.1%	1.99 [0.63, 3.35]
Lee, 2014a	58.9	7.2	14	53.8	7.3	14	16.2%	0.68 [-0.08, 1.45]
Lee, 2014b	58.9	7.2	14	54.6	6.7	14	16.5%	0.60 [-0.16, 1.36]
Snow & Williams, 1994a	19.2	9.6	11	13.3	6.5	11	12.7%	0.69 [-0.17, 1.56]
Snow & Williams, 1994b	19.2	9.6	11	12.5	8.3	11	12.6%	0.72 [-0.15, 1.59]
**Total (95% CI)**			**92**			**92**	**100.0%**	**0.84 [0.53, 1.15]**
Heterogeneity: Chi² = 4.94, df = 9 (P = 0.84); I² = 0%								
Test for overall effect: Z = 5.35 (P < 0.00001)								

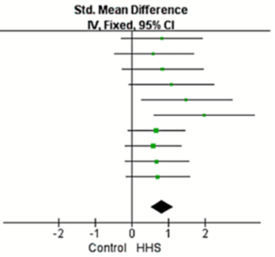

Online supplemental appendix 3 Figure 4 Meta-analysis of knee kinematics during walking in high-heeled shoes compared with flat shoes or barefoot. *IV* inverse variance, *CI* confidence interval, *HHS* high-heeled shoes, *ROM* range of motion.

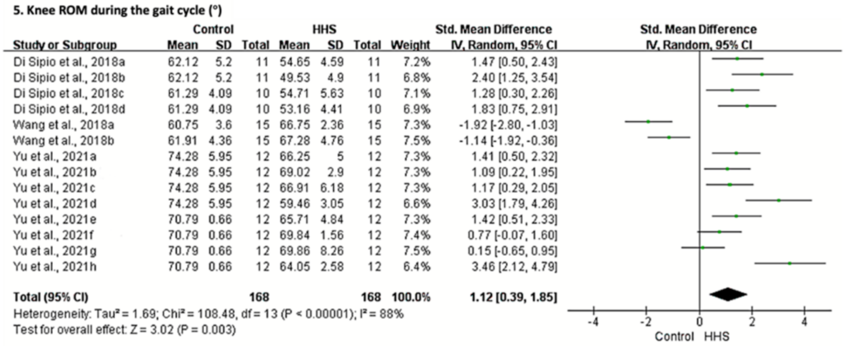

5. Knee ROM during the gait cycle (°)

Study or Subgroup	Control Mean	Control SD	Control Total	HHS Mean	HHS SD	HHS Total	Weight	Std. Mean Difference IV, Random, 95% CI
Di Sipio et al., 2018a	62.12	5.2	11	54.65	4.59	11	7.2%	1.47 [0.50, 2.43]
Di Sipio et al., 2018b	62.12	5.2	11	49.53	4.9	11	6.8%	2.40 [1.25, 3.54]
Di Sipio et al., 2018c	61.29	4.09	10	54.71	5.63	10	7.1%	1.28 [0.30, 2.26]
Di Sipio et al., 2018d	61.29	4.09	10	53.16	4.41	10	6.9%	1.83 [0.75, 2.91]
Wang et al., 2018a	60.75	3.6	15	66.75	2.36	15	7.3%	-1.92 [-2.80, -1.03]
Wang et al., 2018b	61.91	4.36	15	67.28	4.76	15	7.5%	-1.14 [-1.92, -0.36]
Yu et al., 2021a	74.28	5.95	12	66.25	5	12	7.3%	1.41 [0.50, 2.32]
Yu et al., 2021b	74.28	5.95	12	69.02	2.9	12	7.3%	1.09 [0.22, 1.95]
Yu et al., 2021c	74.28	5.95	12	66.91	6.18	12	7.3%	1.17 [0.29, 2.05]
Yu et al., 2021d	74.28	5.95	12	59.46	3.05	12	6.6%	3.03 [1.79, 4.26]
Yu et al., 2021e	70.79	0.66	12	65.71	4.84	12	7.3%	1.42 [0.51, 2.33]
Yu et al., 2021f	70.79	0.66	12	69.84	1.56	12	7.4%	0.77 [-0.07, 1.60]
Yu et al., 2021g	70.79	0.66	12	69.86	8.26	12	7.5%	0.15 [-0.65, 0.95]
Yu et al., 2021h	70.79	0.66	12	64.05	2.58	12	6.4%	3.46 [2.12, 4.79]
**Total (95% CI)**			**168**			**168**	**100.0%**	**1.12 [0.39, 1.85]**

Heterogeneity: $Tau^2 = 1.69$; $Chi^2 = 108.48$, df = 13 (P < 0.00001); $I^2 = 88\%$
Test for overall effect: Z = 3.02 (P = 0.003)

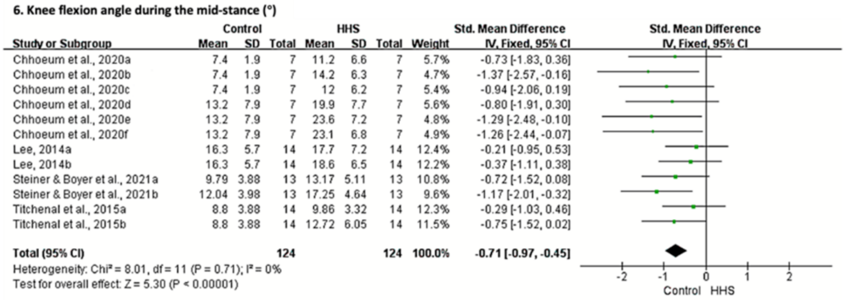

6. Knee flexion angle during the mid-stance (°)

Study or Subgroup	Control Mean	Control SD	Control Total	HHS Mean	HHS SD	HHS Total	Weight	Std. Mean Difference IV, Fixed, 95% CI
Chhoeum et al., 2020a	7.4	1.9	7	11.2	6.6	7	5.7%	-0.73 [-1.83, 0.36]
Chhoeum et al., 2020b	7.4	1.9	7	14.2	6.3	7	4.7%	-1.37 [-2.57, -0.16]
Chhoeum et al., 2020c	7.4	1.9	7	12	6.2	7	5.4%	-0.94 [-2.06, 0.19]
Chhoeum et al., 2020d	13.2	7.9	7	19.9	7.7	7	5.6%	-0.80 [-1.91, 0.30]
Chhoeum et al., 2020e	13.2	7.9	7	23.6	7.2	7	4.8%	-1.29 [-2.48, -0.10]
Chhoeum et al., 2020f	13.2	7.9	7	23.1	6.8	7	4.9%	-1.26 [-2.44, -0.07]
Lee, 2014a	16.3	5.7	14	17.7	7.2	14	12.4%	-0.21 [-0.95, 0.53]
Lee, 2014b	16.3	5.7	14	18.6	6.5	14	12.2%	-0.37 [-1.11, 0.38]
Steiner & Boyer et al., 2021a	9.79	3.88	13	13.17	5.11	13	10.8%	-0.72 [-1.52, 0.08]
Steiner & Boyer et al., 2021b	12.04	3.98	13	17.25	4.64	13	9.6%	-1.17 [-2.01, -0.32]
Titchenal et al., 2015a	8.8	3.88	14	9.86	3.32	14	12.3%	-0.29 [-1.03, 0.46]
Titchenal et al., 2015b	8.8	3.88	14	12.72	6.05	14	11.5%	-0.75 [-1.52, 0.02]
**Total (95% CI)**			**124**			**124**	**100.0%**	**-0.71 [-0.97, -0.45]**

Heterogeneity: $Chi^2 = 8.01$, df = 11 (P = 0.71); $I^2 = 0\%$
Test for overall effect: Z = 5.30 (P < 0.00001)

Online supplemental appendix 3 Figure 5 Meta-analysis of knee kinematics during walking in high-heeled shoes compared with flat shoes or barefoot. *IV* inverse variance, *CI* confidence interval, *HHS* high-heeled shoes, *ROM* range of motion.

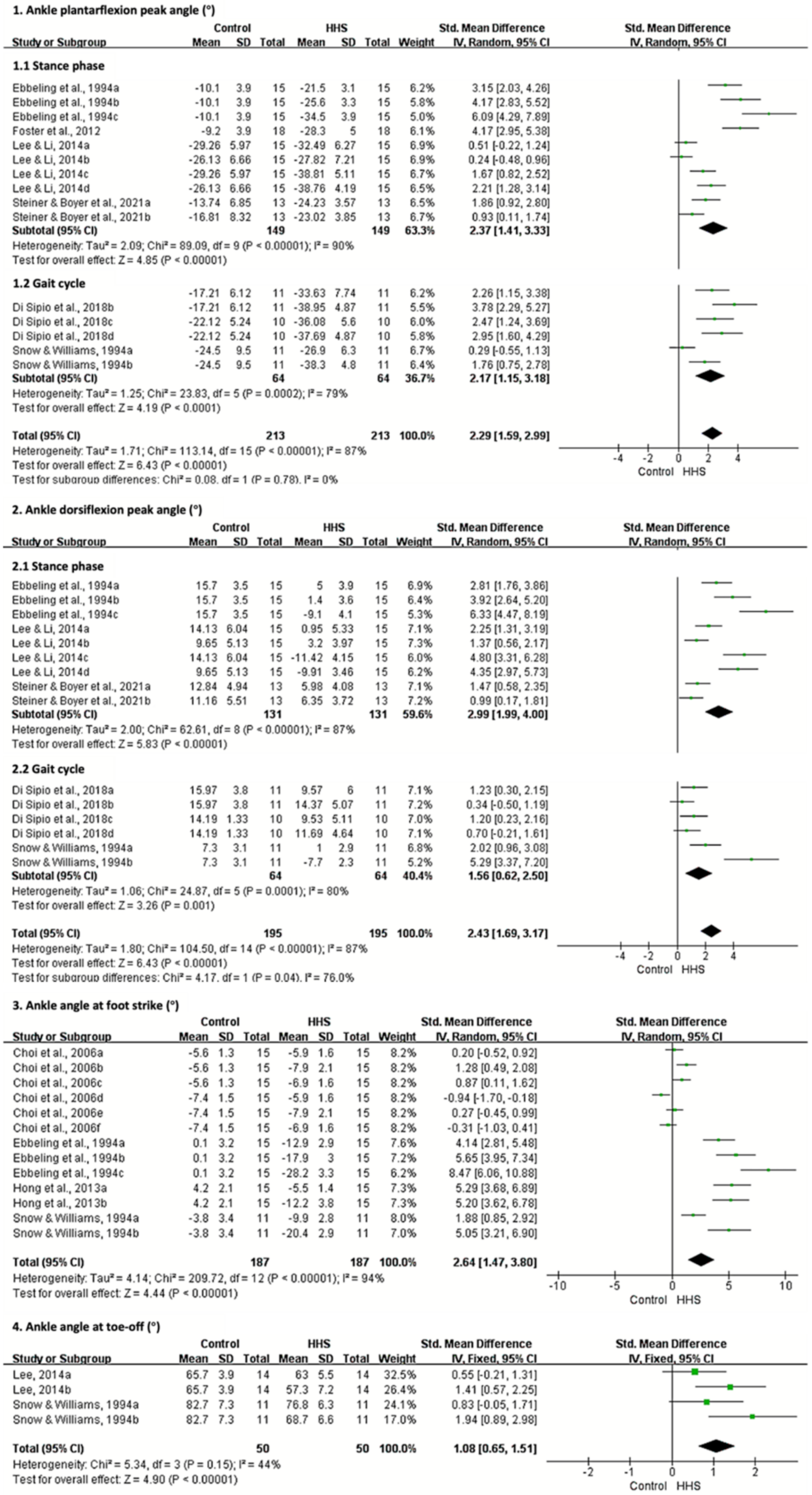

1. Ankle plantarflexion peak angle (°)

	Control			HHS				Std. Mean Difference
Study or Subgroup	Mean	SD	Total	Mean	SD	Total	Weight	IV, Random, 95% CI
**1.1 Stance phase**								
Ebbeling et al., 1994a	-10.1	3.9	15	-21.5	3.1	15	6.2%	3.15 [2.03, 4.26]
Ebbeling et al., 1994b	-10.1	3.9	15	-25.6	3.3	15	5.8%	4.17 [2.83, 5.52]
Ebbeling et al., 1994c	-10.1	3.9	15	-34.5	3.9	15	5.0%	6.09 [4.29, 7.89]
Foster et al., 2012	-9.2	3.9	18	-28.3	5	18	6.1%	4.17 [2.95, 5.38]
Lee & Li, 2014a	-29.26	5.97	15	-32.49	6.27	15	6.9%	0.51 [-0.22, 1.24]
Lee & Li, 2014b	-26.13	6.66	15	-27.82	7.21	15	6.9%	0.24 [-0.48, 0.96]
Lee & Li, 2014c	-29.26	5.97	15	-38.81	5.11	15	6.7%	1.67 [0.82, 2.52]
Lee & Li, 2014d	-26.13	6.66	15	-38.76	4.19	15	6.5%	2.21 [1.28, 3.14]
Steiner & Boyer et al., 2021a	-13.74	6.85	13	-24.23	3.57	13	6.5%	1.86 [0.92, 2.80]
Steiner & Boyer et al., 2021b	-16.81	8.32	13	-23.02	3.85	13	6.7%	0.93 [0.11, 1.74]
**Subtotal (95% CI)**			**149**			**149**	**63.3%**	**2.37 [1.41, 3.33]**
Heterogeneity: Tau² = 2.09; Chi² = 89.09, df = 9 (P < 0.00001); I² = 90%								
Test for overall effect: Z = 4.85 (P < 0.00001)								
**1.2 Gait cycle**								
	-17.21	6.12	11	-33.63	7.74	11	6.2%	2.26 [1.15, 3.38]
Di Sipio et al., 2018b	-17.21	6.12	11	-38.95	4.87	11	5.5%	3.78 [2.29, 5.27]
Di Sipio et al., 2018c	-22.12	5.24	10	-36.08	5.6	10	6.0%	2.47 [1.24, 3.69]
Di Sipio et al., 2018d	-22.12	5.24	10	-37.69	4.87	10	5.8%	2.95 [1.60, 4.29]
Snow & Williams, 1994a	-24.5	9.5	11	-26.9	6.3	11	6.7%	0.29 [-0.55, 1.13]
Snow & Williams, 1994b	-24.5	9.5	11	-38.3	4.8	11	6.4%	1.76 [0.75, 2.78]
**Subtotal (95% CI)**			**64**			**64**	**36.7%**	**2.17 [1.15, 3.18]**
Heterogeneity: Tau² = 1.25; Chi² = 23.83, df = 5 (P = 0.0002); I² = 79%								
Test for overall effect: Z = 4.19 (P < 0.0001)								
**Total (95% CI)**			**213**			**213**	**100.0%**	**2.29 [1.59, 2.99]**
Heterogeneity: Tau² = 1.71; Chi² = 113.14, df = 15 (P < 0.00001); I² = 87%								
Test for overall effect: Z = 6.43 (P < 0.00001)								
Test for subgroup differences: Chi² = 0.08, df = 1 (P = 0.78), I² = 0%								

2. Ankle dorsiflexion peak angle (°)

	Control			HHS				Std. Mean Difference
Study or Subgroup	Mean	SD	Total	Mean	SD	Total	Weight	IV, Random, 95% CI
**2.1 Stance phase**								
Ebbeling et al., 1994a	15.7	3.5	15	5	3.9	15	6.9%	2.81 [1.76, 3.86]
Ebbeling et al., 1994b	15.7	3.5	15	1.4	3.6	15	6.4%	3.92 [2.64, 5.20]
Ebbeling et al., 1994c	15.7	3.5	15	-9.1	4.1	15	5.3%	6.33 [4.47, 8.19]
Lee & Li, 2014a	14.13	6.04	15	0.95	5.33	15	7.1%	2.25 [1.31, 3.19]
Lee & Li, 2014b	9.65	5.13	15	3.2	3.97	15	7.3%	1.37 [0.56, 2.17]
Lee & Li, 2014c	14.13	6.04	15	-11.42	4.15	15	6.0%	4.80 [3.31, 6.28]
Lee & Li, 2014d	9.65	5.13	15	-9.91	3.46	15	6.2%	4.35 [2.97, 5.73]
Steiner & Boyer et al., 2021a	12.84	4.94	13	5.98	4.08	13	7.1%	1.47 [0.58, 2.35]
Steiner & Boyer et al., 2021b	11.16	5.51	13	6.35	3.72	13	7.2%	0.99 [0.17, 1.81]
**Subtotal (95% CI)**			**131**			**131**	**59.6%**	**2.99 [1.99, 4.00]**
Heterogeneity: Tau² = 2.00; Chi² = 62.61, df = 8 (P < 0.00001); I² = 87%								
Test for overall effect: Z = 5.83 (P < 0.00001)								
**2.2 Gait cycle**								
Di Sipio et al., 2018a	15.97	3.8	11	9.57	6	11	7.1%	1.23 [0.30, 2.15]
Di Sipio et al., 2018b	15.97	3.8	11	14.37	5.07	11	7.2%	0.34 [-0.50, 1.19]
Di Sipio et al., 2018c	14.19	1.33	10	9.53	5.11	10	7.0%	1.20 [0.23, 2.16]
Di Sipio et al., 2018d	14.19	1.33	10	11.69	4.64	10	7.1%	0.70 [-0.21, 1.61]
Snow & Williams, 1994a	7.3	3.1	11	1	2.9	11	6.8%	2.02 [0.96, 3.08]
Snow & Williams, 1994b	7.3	3.1	11	-7.7	2.3	11	5.2%	5.29 [3.37, 7.20]
**Subtotal (95% CI)**			**64**			**64**	**40.4%**	**1.56 [0.62, 2.50]**
Heterogeneity: Tau² = 1.06; Chi² = 24.87, df = 5 (P = 0.0001); I² = 80%								
Test for overall effect: Z = 3.26 (P = 0.001)								
**Total (95% CI)**			**195**			**195**	**100.0%**	**2.43 [1.69, 3.17]**
Heterogeneity: Tau² = 1.80; Chi² = 104.50, df = 14 (P < 0.00001); I² = 87%								
Test for overall effect: Z = 6.43 (P < 0.00001)								
Test for subgroup differences: Chi² = 4.17, df = 1 (P = 0.04), I² = 76.0%								

3. Ankle angle at foot strike (°)

	Control			HHS				Std. Mean Difference
Study or Subgroup	Mean	SD	Total	Mean	SD	Total	Weight	IV, Random, 95% CI
Choi et al., 2006a	-5.6	1.3	15	-5.9	1.6	15	8.2%	0.20 [-0.52, 0.92]
Choi et al., 2006b	-5.6	1.3	15	-7.9	2.1	15	8.2%	1.28 [0.49, 2.08]
Choi et al., 2006c	-5.6	1.3	15	-6.9	1.6	15	8.2%	0.87 [0.11, 1.62]
Choi et al., 2006d	-7.4	1.5	15	-5.9	1.6	15	8.2%	-0.94 [-1.70, -0.18]
Choi et al., 2006e	-7.4	1.5	15	-7.9	2.1	15	8.2%	0.27 [-0.45, 0.99]
Choi et al., 2006f	-7.4	1.5	15	-6.9	1.6	15	8.2%	-0.31 [-1.03, 0.41]
Ebbeling et al., 1994a	0.1	3.2	15	-12.9	2.9	15	7.6%	4.14 [2.81, 5.48]
Ebbeling et al., 1994b	0.1	3.2	15	-17.9	3	15	7.2%	5.65 [3.95, 7.34]
Ebbeling et al., 1994c	0.1	3.2	15	-28.2	3.3	15	6.2%	8.47 [6.06, 10.88]
Hong et al., 2013a	4.2	2.1	15	-5.5	1.4	15	7.3%	5.29 [3.68, 6.89]
Hong et al., 2013b	4.2	2.1	15	-12.2	3.8	15	7.3%	5.20 [3.62, 6.78]
Snow & Williams, 1994a	-3.8	3.4	11	-9.9	2.8	11	8.0%	1.88 [0.85, 2.92]
Snow & Williams, 1994b	-3.8	3.4	11	-20.4	2.9	11	7.0%	5.05 [3.21, 6.90]
**Total (95% CI)**			**187**			**187**	**100.0%**	**2.64 [1.47, 3.80]**
Heterogeneity: Tau² = 4.14; Chi² = 209.72, df = 12 (P < 0.00001); I² = 94%								
Test for overall effect: Z = 4.44 (P < 0.00001)								

4. Ankle angle at toe-off (°)

	Control			HHS				Std. Mean Difference
Study or Subgroup	Mean	SD	Total	Mean	SD	Total	Weight	IV, Fixed, 95% CI
Lee, 2014a	65.7	3.9	14	63	5.5	14	32.5%	0.55 [-0.21, 1.31]
Lee, 2014b	65.7	3.9	14	57.3	7.2	14	26.4%	1.41 [0.57, 2.25]
Snow & Williams, 1994a	82.7	7.3	11	76.8	6.3	11	24.1%	0.83 [-0.05, 1.71]
Snow & Williams, 1994b	82.7	7.3	11	68.7	6.6	11	17.0%	1.94 [0.89, 2.98]
**Total (95% CI)**			**50**			**50**	**100.0%**	**1.08 [0.65, 1.51]**
Heterogeneity: Chi² = 5.34, df = 3 (P = 0.15); I² = 44%								
Test for overall effect: Z = 4.90 (P < 0.00001)								

Online supplemental appendix 3 Figure 6 Meta-analysis of foot-ankle complex kinematics during walking in high-heeled shoes compared with flat shoes or barefoot. *IV* inverse variance, *CI* confidence interval, *HHS* high-heeled shoes, *ROM* range of motion.

5. Ankle ROM during the gait cycle (°)

Study or Subgroup	Control Mean	Control SD	Control Total	HHS Mean	HHS SD	HHS Total	Weight	Std. Mean Difference IV, Random, 95% CI
Di Sipio et al., 2018a	33.19	4.17	11	25.23	3.25	11	8.6%	2.05 [0.98, 3.12]
Di Sipio et al., 2018b	33.19	4.17	11	24.55	2.97	11	8.4%	2.30 [1.17, 3.42]
Di Sipio et al., 2018c	36.3	4.78	10	26.55	3.84	10	8.3%	2.15 [1.00, 3.30]
Di Sipio et al., 2018d	36.3	4.78	10	25.99	3.99	10	8.2%	2.24 [1.07, 3.41]
Yu et al., 2021a	47.47	0.79	12	47.31	0.65	12	9.6%	0.21 [-0.59, 1.02]
Yu et al., 2021b	47.47	0.79	12	33.42	1.08	12	1.8%	14.34 [9.83, 18.85]
Yu et al., 2021c	47.47	0.79	12	39.84	5.96	12	9.0%	1.73 [0.77, 2.70]
Yu et al., 2021d	47.47	0.79	12	40.31	5.88	12	9.0%	1.65 [0.70, 2.60]
Yu et al., 2021e	47.59	5.4	12	44.32	6.02	12	9.5%	0.55 [-0.27, 1.37]
Yu et al., 2021f	47.59	5.4	12	39.97	5.59	12	9.2%	1.34 [0.44, 2.24]
Yu et al., 2021g	47.59	5.4	12	39.69	6.39	12	9.2%	1.29 [0.40, 2.18]
Yu et al., 2021h	47.59	5.4	12	41.13	4.96	12	9.3%	1.20 [0.32, 2.09]
**Total (95% CI)**			**138**			**138**	**100.0%**	**1.71 [1.06, 2.36]**

Heterogeneity: Tau² = 0.98; Chi² = 52.64, df = 11 (P < 0.00001); I² = 79%
Test for overall effect: Z = 5.17 (P < 0.00001)

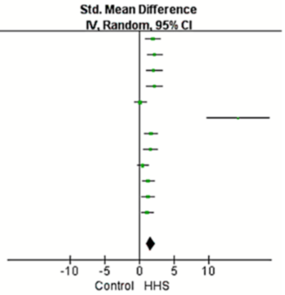

6. Rearfoot angle at foot strike (°)

Study or Subgroup	Control Mean	Control SD	Control Total	HHS Mean	HHS SD	HHS Total	Weight	Std. Mean Difference IV, Fixed, 95% CI
Ebbeling et al., 1994a	-0.4	3.8	15	0.5	2.8	15	23.0%	-0.26 [-0.98, 0.46]
Ebbeling et al., 1994b	-0.4	3.8	15	-1.7	3.4	15	22.8%	0.35 [-0.37, 1.07]
Ebbeling et al., 1994c	-0.4	3.8	15	1.4	5.5	15	22.7%	-0.37 [-1.09, 0.35]
Snow & Williams, 1994a	2.3	3.7	11	4.4	3.3	11	16.2%	-0.58 [-1.43, 0.28]
Snow & Williams, 1994b	2.3	3.7	11	5.5	3.5	11	15.3%	-0.85 [-1.74, 0.03]
**Total (95% CI)**			**67**			**67**	**100.0%**	**-0.29 [-0.63, 0.06]**

Heterogeneity: Chi² = 5.09, df = 4 (P = 0.28); I² = 21%
Test for overall effect: Z = 1.64 (P = 0.10)

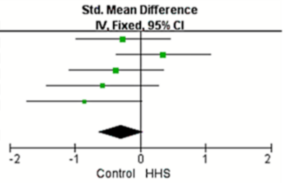

7. Rearfoot inversion peak angle during the stance phase (°)

Study or Subgroup	Control Mean	Control SD	Control Total	HHS Mean	HHS SD	HHS Total	Weight	Std. Mean Difference IV, Random, 95% CI
Foster et al., 2012	3.1	4.2	18	9.1	6.2	18	20.4%	-1.11 [-1.82, -0.40]
Lee & Li, 2014a	15.07	5.1	15	12.29	3.45	15	20.3%	0.62 [-0.11, 1.36]
Lee & Li, 2014b	9.37	2.64	15	1.77	3.21	15	19.1%	2.52 [1.53, 3.51]
Lee & Li, 2014c	15.07	5.1	15	17.24	4.16	15	20.3%	-0.45 [-1.18, 0.27]
Lee & Li, 2014d	9.37	2.64	15	6.22	1.46	15	19.9%	1.44 [0.62, 2.25]
**Total (95% CI)**			**78**			**78**	**100.0%**	**0.57 [-0.62, 1.77]**

Heterogeneity: Tau² = 1.70; Chi² = 46.79, df = 4 (P < 0.00001); I² = 91%
Test for overall effect: Z = 0.94 (P = 0.35)

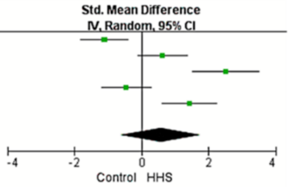

Online supplemental appendix 3 Figure 7 Meta-analysis of foot-ankle complex kinematics during walking in high-heeled shoes compared with flat shoes or barefoot. *IV* inverse variance, *CI* confidence interval, *HHS* high-heeled shoes, *ROM* range of motion.

1. Hip flexion peak moment during the gait cycle (N·m/kg)

Study or Subgroup	Control Mean	Control SD	Control Total	HHS Mean	HHS SD	HHS Total	Weight	Std. Mean Difference IV, Fixed, 95% CI
Di Sipio et al., 2018a	0.98	0.25	11	1.05	0.28	11	12.6%	-0.25 [-1.09, 0.59]
Di Sipio et al., 2018b	0.98	0.25	11	1.17	0.41	11	12.2%	-0.54 [-1.39, 0.32]
Di Sipio et al., 2018c	1.02	0.31	10	0.98	0.16	10	11.6%	0.16 [-0.72, 1.03]
Di Sipio et al., 2018d	1.02	0.31	10	0.98	0.16	10	11.6%	0.16 [-0.72, 1.03]
Esenyel et al., 2003	1.07	0.26	15	1.11	0.29	15	17.4%	-0.14 [-0.86, 0.58]
Lee et al., 2016a	0.921	1.33	15	0.785	1.22	15	17.4%	0.10 [-0.61, 0.82]
Lee et al., 2016b	0.886	0.89	15	1.118	1.33	15	17.3%	-0.20 [-0.92, 0.52]
**Total (95% CI)**			**87**			**87**	**100.0%**	**-0.10 [-0.40, 0.20]**

Heterogeneity: Chi² = 2.19, df = 6 (P = 0.90); I² = 0%
Test for overall effect: Z = 0.68 (P = 0.50)

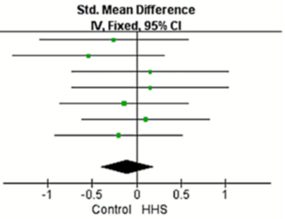

2. Hip extension peak moment during the gait cycle (N·m/kg)

Study or Subgroup	Control Mean	Control SD	Control Total	HHS Mean	HHS SD	HHS Total	Weight	Std. Mean Difference IV, Random, 95% CI
Esenyel et al., 2003	0.52	0.14	15	0.52	0.14	15	34.5%	0.00 [-0.72, 0.72]
Lee et al., 2016a	0.452	0.36	15	1.006	0.21	15	33.7%	-1.83 [-2.70, -0.96]
Lee et al., 2016b	0.471	0.15	15	0.943	0.11	15	31.8%	-3.49 [-4.68, -2.30]
**Total (95% CI)**			**45**			**45**	**100.0%**	**-1.73 [-3.65, 0.20]**

Heterogeneity: Tau² = 2.66; Chi² = 27.09, df = 2 (P < 0.00001); I² = 93%
Test for overall effect: Z = 1.76 (P = 0.08)

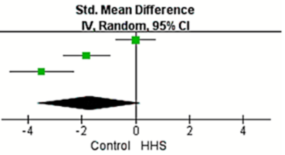

Online supplemental appendix 3 Figure 8 Meta-analysis of hip moment during walking in high-heeled shoes compared with flat shoes or barefoot. *IV* inverse variance, *CI* confidence interval, *HHS* high-heeled shoes.

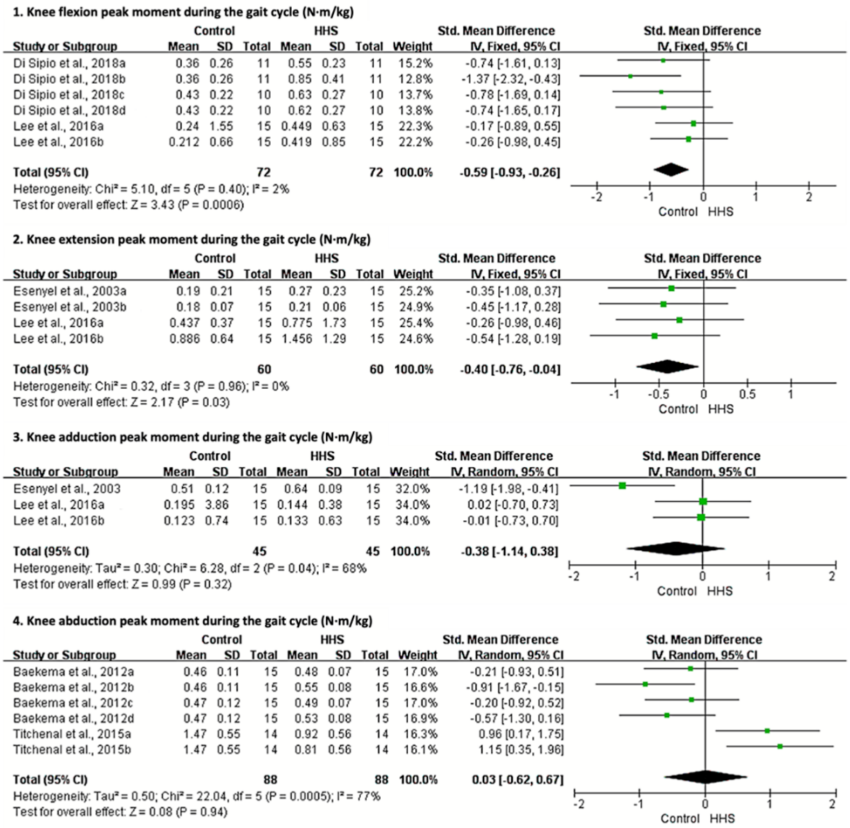

1. Knee flexion peak moment during the gait cycle (N·m/kg)

Study or Subgroup	Control Mean	Control SD	Control Total	HHS Mean	HHS SD	HHS Total	Weight	Std. Mean Difference IV, Fixed, 95% CI
Di Sipio et al., 2018a	0.36	0.26	11	0.55	0.23	11	15.2%	-0.74 [-1.61, 0.13]
Di Sipio et al., 2018b	0.36	0.26	11	0.85	0.41	11	12.8%	-1.37 [-2.32, -0.43]
Di Sipio et al., 2018c	0.43	0.22	10	0.63	0.27	10	13.7%	-0.78 [-1.69, 0.14]
Di Sipio et al., 2018d	0.43	0.22	10	0.62	0.27	10	13.8%	-0.74 [-1.65, 0.17]
Lee et al., 2016a	0.24	1.55	15	0.449	0.63	15	22.3%	-0.17 [-0.89, 0.55]
Lee et al., 2016b	0.212	0.66	15	0.419	0.85	15	22.2%	-0.26 [-0.98, 0.45]
**Total (95% CI)**			**72**			**72**	**100.0%**	**-0.59 [-0.93, -0.26]**

Heterogeneity: Chi² = 5.10, df = 5 (P = 0.40); I² = 2%
Test for overall effect: Z = 3.43 (P = 0.0006)

2. Knee extension peak moment during the gait cycle (N·m/kg)

Study or Subgroup	Control Mean	Control SD	Control Total	HHS Mean	HHS SD	HHS Total	Weight	Std. Mean Difference IV, Fixed, 95% CI
Esenyel et al., 2003a	0.19	0.21	15	0.27	0.23	15	25.2%	-0.35 [-1.08, 0.37]
Esenyel et al., 2003b	0.18	0.07	15	0.21	0.06	15	24.9%	-0.45 [-1.17, 0.28]
Lee et al., 2016a	0.437	0.37	15	0.775	1.73	15	25.4%	-0.26 [-0.98, 0.46]
Lee et al., 2016b	0.886	0.64	15	1.456	1.29	15	24.6%	-0.54 [-1.28, 0.19]
**Total (95% CI)**			**60**			**60**	**100.0%**	**-0.40 [-0.76, -0.04]**

Heterogeneity: Chi² = 0.32, df = 3 (P = 0.96); I² = 0%
Test for overall effect: Z = 2.17 (P = 0.03)

3. Knee adduction peak moment during the gait cycle (N·m/kg)

Study or Subgroup	Control Mean	Control SD	Control Total	HHS Mean	HHS SD	HHS Total	Weight	Std. Mean Difference IV, Random, 95% CI
Esenyel et al., 2003	0.51	0.12	15	0.64	0.09	15	32.0%	-1.19 [-1.98, -0.41]
Lee et al., 2016a	0.195	3.86	15	0.144	0.38	15	34.0%	0.02 [-0.70, 0.73]
Lee et al., 2016b	0.123	0.74	15	0.133	0.63	15	34.0%	-0.01 [-0.73, 0.70]
**Total (95% CI)**			**45**			**45**	**100.0%**	**-0.38 [-1.14, 0.38]**

Heterogeneity: Tau² = 0.30; Chi² = 6.28, df = 2 (P = 0.04); I² = 68%
Test for overall effect: Z = 0.99 (P = 0.32)

4. Knee abduction peak moment during the gait cycle (N·m/kg)

Study or Subgroup	Control Mean	Control SD	Control Total	HHS Mean	HHS SD	HHS Total	Weight	Std. Mean Difference IV, Random, 95% CI
Baekema et al., 2012a	0.46	0.11	15	0.48	0.07	15	17.0%	-0.21 [-0.93, 0.51]
Baekema et al., 2012b	0.46	0.11	15	0.55	0.08	15	16.6%	-0.91 [-1.67, -0.15]
Baekema et al., 2012c	0.47	0.12	15	0.49	0.07	15	17.0%	-0.20 [-0.92, 0.52]
Baekema et al., 2012d	0.47	0.12	15	0.53	0.08	15	16.9%	-0.57 [-1.30, 0.16]
Titchenal et al., 2015a	1.47	0.55	14	0.92	0.56	14	16.3%	0.96 [0.17, 1.75]
Titchenal et al., 2015b	1.47	0.55	14	0.81	0.56	14	16.1%	1.15 [0.35, 1.96]
**Total (95% CI)**			**88**			**88**	**100.0%**	**0.03 [-0.62, 0.67]**

Heterogeneity: Tau² = 0.50; Chi² = 22.04, df = 5 (P = 0.0005); I² = 77%
Test for overall effect: Z = 0.08 (P = 0.94)

Online supplemental appendix 3 Figure 9 Meta-analysis of knee moment during walking in high-heeled shoes compared with flat shoes or barefoot. *IV* inverse variance, *CI* confidence interval, *HHS* high-heeled shoes.

1. Ankle plantarflexion peak moment during the gait cycle (N·m/kg)

Study or Subgroup	Control Mean	Control SD	Control Total	HHS Mean	HHS SD	HHS Total	Weight	Std. Mean Difference IV, Random, 95% CI
Di Sipio et al., 2018a	1.36	0.25	11	1.17	0.19	11	14.2%	0.82 [-0.05, 1.70]
Di Sipio et al., 2018b	1.36	0.25	11	1.2	0.43	11	14.6%	0.44 [-0.41, 1.29]
Di Sipio et al., 2018c	1.54	0.18	10	1.17	0.18	10	11.4%	1.97 [0.86, 3.08]
Di Sipio et al., 2018d	1.54	0.18	10	1.11	0.24	10	11.5%	1.94 [0.84, 3.05]
Esenyel et al., 2003	1.56	0.13	15	1.34	0.2	15	15.4%	1.27 [0.47, 2.06]
Lee et al., 2016a	1.515	2.83	15	1.075	0.59	15	16.4%	0.21 [-0.51, 0.93]
Lee et al., 2016b	1.472	0.97	15	1.124	1.72	15	16.4%	0.24 [-0.48, 0.96]
**Total (95% CI)**			**87**			**87**	**100.0%**	**0.90 [0.38, 1.42]**

Heterogeneity: $Tau^2 = 0.29$; $Chi^2 = 15.32$, df = 6 (P = 0.02); $I^2 = 61\%$
Test for overall effect: Z = 3.39 (P = 0.0007)

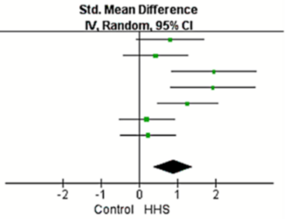

2. Ankle dorsiflexion peak moment during the gait cycle (N·m/kg)

Study or Subgroup	Control Mean	Control SD	Control Total	HHS Mean	HHS SD	HHS Total	Weight	Std. Mean Difference IV, Fixed, 95% CI
Esenyel et al., 2003	0.11	0.07	15	0.16	0.1	15	32.3%	-0.56 [-1.30, 0.17]
Lee et al., 2016a	0.166	1.2	15	0.185	0.94	15	33.8%	-0.02 [-0.73, 0.70]
Lee et al., 2016b	0.241	1.97	15	0.244	1.37	15	33.8%	-0.00 [-0.72, 0.71]
**Total (95% CI)**			**45**			**45**	**100.0%**	**-0.19 [-0.60, 0.23]**

Heterogeneity: $Chi^2 = 1.49$, df = 2 (P = 0.47); $I^2 = 0\%$
Test for overall effect: Z = 0.89 (P = 0.37)

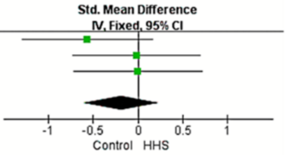

Online supplemental appendix 3 Figure 10 Meta-analysis of ankle moment during walking in high-heeled shoes compared with flat shoes or barefoot. *IV* inverse variance, *CI* confidence interval, *HHS* high-heeled shoes.

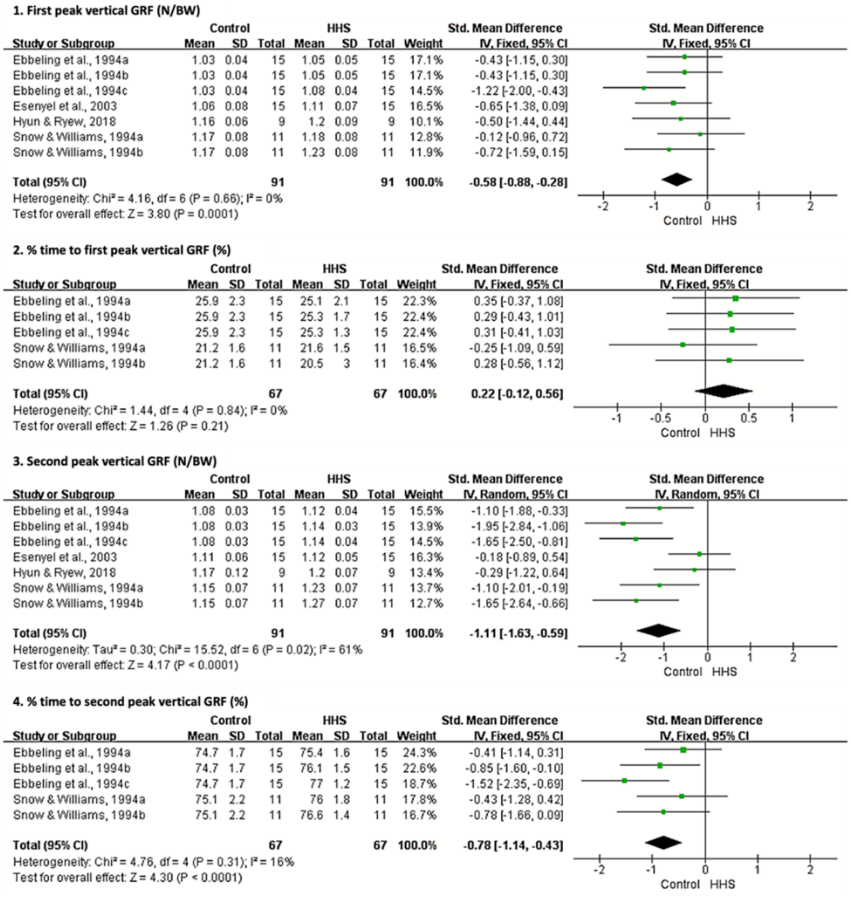

1. First peak vertical GRF (N/BW)

	Control			HHS				Std. Mean Difference
Study or Subgroup	Mean	SD	Total	Mean	SD	Total	Weight	IV, Fixed, 95% CI
Ebbeling et al., 1994a	1.03	0.04	15	1.05	0.05	15	17.1%	-0.43 [-1.15, 0.30]
Ebbeling et al., 1994b	1.03	0.04	15	1.05	0.05	15	17.1%	-0.43 [-1.15, 0.30]
Ebbeling et al., 1994c	1.03	0.04	15	1.08	0.04	15	14.5%	-1.22 [-2.00, -0.43]
Esenyel et al., 2003	1.06	0.08	15	1.11	0.07	15	16.5%	-0.65 [-1.38, 0.09]
Hyun & Ryew, 2018	1.16	0.06	9	1.2	0.09	9	10.1%	-0.50 [-1.44, 0.44]
Snow & Williams, 1994a	1.17	0.08	11	1.18	0.08	11	12.8%	-0.12 [-0.96, 0.72]
Snow & Williams, 1994b	1.17	0.08	11	1.23	0.08	11	11.9%	-0.72 [-1.59, 0.15]
**Total (95% CI)**			**91**			**91**	**100.0%**	**-0.58 [-0.88, -0.28]**

Heterogeneity: $Chi^2 = 4.16$, df = 6 (P = 0.66); $I^2 = 0\%$
Test for overall effect: Z = 3.80 (P = 0.0001)

2. % time to first peak vertical GRF (%)

	Control			HHS				Std. Mean Difference
Study or Subgroup	Mean	SD	Total	Mean	SD	Total	Weight	IV, Fixed, 95% CI
Ebbeling et al., 1994a	25.9	2.3	15	25.1	2.1	15	22.3%	0.35 [-0.37, 1.08]
Ebbeling et al., 1994b	25.9	2.3	15	25.3	1.7	15	22.4%	0.29 [-0.43, 1.01]
Ebbeling et al., 1994c	25.9	2.3	15	25.3	1.3	15	22.4%	0.31 [-0.41, 1.03]
Snow & Williams, 1994a	21.2	1.6	11	21.6	1.5	11	16.5%	-0.25 [-1.09, 0.59]
Snow & Williams, 1994b	21.2	1.6	11	20.5	3	11	16.4%	0.28 [-0.56, 1.12]
**Total (95% CI)**			**67**			**67**	**100.0%**	**0.22 [-0.12, 0.56]**

Heterogeneity: $Chi^2 = 1.44$, df = 4 (P = 0.84); $I^2 = 0\%$
Test for overall effect: Z = 1.26 (P = 0.21)

3. Second peak vertical GRF (N/BW)

	Control			HHS				Std. Mean Difference
Study or Subgroup	Mean	SD	Total	Mean	SD	Total	Weight	IV, Random, 95% CI
Ebbeling et al., 1994a	1.08	0.03	15	1.12	0.04	15	15.5%	-1.10 [-1.88, -0.33]
Ebbeling et al., 1994b	1.08	0.03	15	1.14	0.03	15	13.9%	-1.95 [-2.84, -1.06]
Ebbeling et al., 1994c	1.08	0.03	15	1.14	0.04	15	14.5%	-1.65 [-2.50, -0.81]
Esenyel et al., 2003	1.11	0.06	15	1.12	0.05	15	16.3%	-0.18 [-0.89, 0.54]
Hyun & Ryew, 2018	1.17	0.12	9	1.2	0.07	9	13.4%	-0.29 [-1.22, 0.64]
Snow & Williams, 1994a	1.15	0.07	11	1.23	0.07	11	13.7%	-1.10 [-2.01, -0.19]
Snow & Williams, 1994b	1.15	0.07	11	1.27	0.07	11	12.7%	-1.65 [-2.64, -0.66]
**Total (95% CI)**			**91**			**91**	**100.0%**	**-1.11 [-1.63, -0.59]**

Heterogeneity: $Tau^2 = 0.30$; $Chi^2 = 15.52$, df = 6 (P = 0.02); $I^2 = 61\%$
Test for overall effect: Z = 4.17 (P < 0.0001)

4. % time to second peak vertical GRF (%)

	Control			HHS				Std. Mean Difference
Study or Subgroup	Mean	SD	Total	Mean	SD	Total	Weight	IV, Fixed, 95% CI
Ebbeling et al., 1994a	74.7	1.7	15	75.4	1.6	15	24.3%	-0.41 [-1.14, 0.31]
Ebbeling et al., 1994b	74.7	1.7	15	76.1	1.5	15	22.6%	-0.85 [-1.60, -0.10]
Ebbeling et al., 1994c	74.7	1.7	15	77	1.2	15	18.7%	-1.52 [-2.35, -0.69]
Snow & Williams, 1994a	75.1	2.2	11	76	1.8	11	17.8%	-0.43 [-1.28, 0.42]
Snow & Williams, 1994b	75.1	2.2	11	76.6	1.4	11	16.7%	-0.78 [-1.66, 0.09]
**Total (95% CI)**			**67**			**67**	**100.0%**	**-0.78 [-1.14, -0.43]**

Heterogeneity: $Chi^2 = 4.76$, df = 4 (P = 0.31); $I^2 = 16\%$
Test for overall effect: Z = 4.30 (P < 0.0001)

Online supplemental appendix 3 Figure 11 Meta-analysis of ground reaction forces during walking in high-heeled shoes compared with flat shoes or barefoot. *IV* inverse variance, *CI* confidence interval, *HHS* high-heeled shoes, *GRF* ground reaction force.

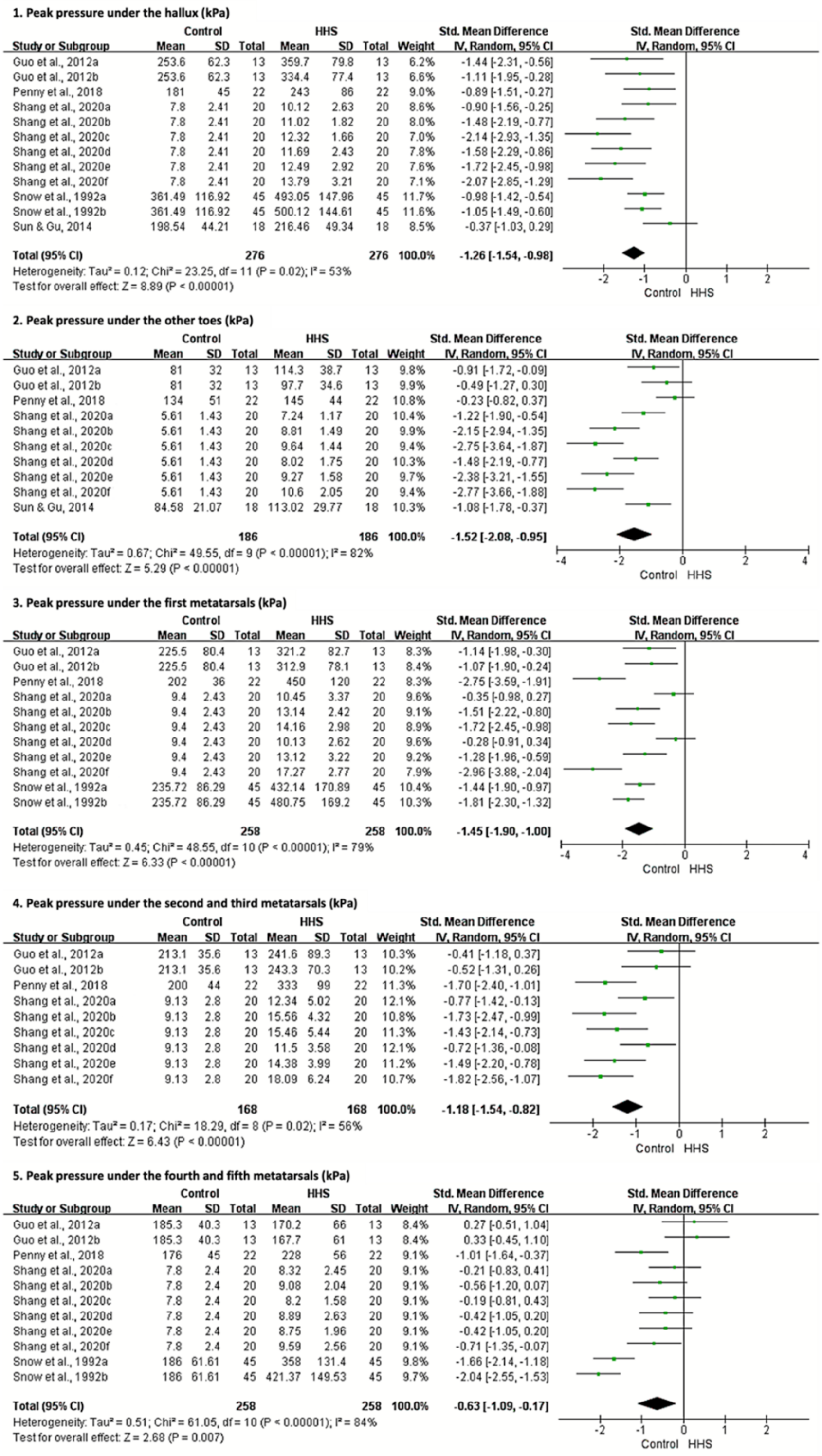

1. Peak pressure under the hallux (kPa)

Study or Subgroup	Control Mean	Control SD	Control Total	HHS Mean	HHS SD	HHS Total	Weight	Std. Mean Difference IV, Random, 95% CI
Guo et al., 2012a	253.6	62.3	13	359.7	79.8	13	6.2%	-1.44 [-2.31, -0.56]
Guo et al., 2012b	253.6	62.3	13	334.4	77.4	13	6.6%	-1.11 [-1.95, -0.28]
Penny et al., 2018	181	45	22	243	86	22	9.0%	-0.89 [-1.51, -0.27]
Shang et al., 2020a	7.8	2.41	20	10.12	2.63	20	8.6%	-0.90 [-1.56, -0.25]
Shang et al., 2020b	7.8	2.41	20	11.02	1.82	20	8.0%	-1.48 [-2.19, -0.77]
Shang et al., 2020c	7.8	2.41	20	12.32	1.66	20	7.0%	-2.14 [-2.93, -1.35]
Shang et al., 2020d	7.8	2.41	20	11.69	2.43	20	7.8%	-1.58 [-2.29, -0.86]
Shang et al., 2020e	7.8	2.41	20	12.49	2.92	20	7.6%	-1.72 [-2.45, -0.98]
Shang et al., 2020f	7.8	2.41	20	13.79	3.21	20	7.1%	-2.07 [-2.85, -1.29]
Snow et al., 1992a	361.49	116.92	45	493.05	147.96	45	11.7%	-0.98 [-1.42, -0.54]
Snow et al., 1992b	361.49	116.92	45	500.12	144.61	45	11.6%	-1.05 [-1.49, -0.60]
Sun & Gu, 2014	198.54	44.21	18	216.46	49.34	18	8.5%	-0.37 [-1.03, 0.29]
**Total (95% CI)**			**276**			**276**	**100.0%**	**-1.26 [-1.54, -0.98]**

Heterogeneity: $Tau^2 = 0.12$; $Chi^2 = 23.25$, df = 11 (P = 0.02); $I^2 = 53\%$
Test for overall effect: Z = 8.89 (P < 0.00001)

2. Peak pressure under the other toes (kPa)

Study or Subgroup	Control Mean	Control SD	Control Total	HHS Mean	HHS SD	HHS Total	Weight	Std. Mean Difference IV, Random, 95% CI
Guo et al., 2012a	81	32	13	114.3	38.7	13	9.8%	-0.91 [-1.72, -0.09]
Guo et al., 2012b	81	32	13	97.7	34.6	13	9.9%	-0.49 [-1.27, 0.30]
Penny et al., 2018	134	51	22	145	44	22	10.8%	-0.23 [-0.82, 0.37]
Shang et al., 2020a	5.61	1.43	20	7.24	1.17	20	10.4%	-1.22 [-1.90, -0.54]
Shang et al., 2020b	5.61	1.43	20	8.81	1.49	20	9.9%	-2.15 [-2.94, -1.35]
Shang et al., 2020c	5.61	1.43	20	9.64	1.44	20	9.4%	-2.75 [-3.64, -1.87]
Shang et al., 2020d	5.61	1.43	20	8.02	1.75	20	10.3%	-1.48 [-2.19, -0.77]
Shang et al., 2020e	5.61	1.43	20	9.27	1.58	20	9.7%	-2.38 [-3.21, -1.55]
Shang et al., 2020f	5.61	1.43	20	10.6	2.05	20	9.4%	-2.77 [-3.66, -1.88]
Sun & Gu, 2014	84.58	21.07	18	113.02	29.77	18	10.3%	-1.08 [-1.78, -0.37]
**Total (95% CI)**			**186**			**186**	**100.0%**	**-1.52 [-2.08, -0.95]**

Heterogeneity: $Tau^2 = 0.67$; $Chi^2 = 49.55$, df = 9 (P < 0.00001); $I^2 = 82\%$
Test for overall effect: Z = 5.29 (P < 0.00001)

3. Peak pressure under the first metatarsals (kPa)

Study or Subgroup	Control Mean	Control SD	Control Total	HHS Mean	HHS SD	HHS Total	Weight	Std. Mean Difference IV, Random, 95% CI
Guo et al., 2012a	225.5	80.4	13	321.2	82.7	13	8.3%	-1.14 [-1.98, -0.30]
Guo et al., 2012b	225.5	80.4	13	312.9	78.1	13	8.4%	-1.07 [-1.90, -0.24]
Penny et al., 2018	202	36	22	450	120	22	8.3%	-2.75 [-3.59, -1.91]
Shang et al., 2020a	9.4	2.43	20	10.45	3.37	20	9.6%	-0.35 [-0.98, 0.27]
Shang et al., 2020b	9.4	2.43	20	13.14	2.42	20	9.1%	-1.51 [-2.22, -0.80]
Shang et al., 2020c	9.4	2.43	20	14.16	2.98	20	8.9%	-1.72 [-2.45, -0.98]
Shang et al., 2020d	9.4	2.43	20	10.13	2.62	20	9.6%	-0.28 [-0.91, 0.34]
Shang et al., 2020e	9.4	2.43	20	13.12	3.22	20	9.2%	-1.28 [-1.96, -0.59]
Shang et al., 2020f	9.4	2.43	20	17.27	2.77	20	7.9%	-2.96 [-3.88, -2.04]
Snow et al., 1992a	235.72	86.29	45	432.14	170.89	45	10.4%	-1.44 [-1.90, -0.97]
Snow et al., 1992b	235.72	86.29	45	480.75	169.2	45	10.3%	-1.81 [-2.30, -1.32]
**Total (95% CI)**			**258**			**258**	**100.0%**	**-1.45 [-1.90, -1.00]**

Heterogeneity: $Tau^2 = 0.45$; $Chi^2 = 48.55$, df = 10 (P < 0.00001); $I^2 = 79\%$
Test for overall effect: Z = 6.33 (P < 0.00001)

4. Peak pressure under the second and third metatarsals (kPa)

Study or Subgroup	Control Mean	Control SD	Control Total	HHS Mean	HHS SD	HHS Total	Weight	Std. Mean Difference IV, Random, 95% CI
Guo et al., 2012a	213.1	35.6	13	241.6	89.3	13	10.3%	-0.41 [-1.18, 0.37]
Guo et al., 2012b	213.1	35.6	13	243.3	70.3	13	10.2%	-0.52 [-1.31, 0.26]
Penny et al., 2018	200	44	22	333	99	22	11.3%	-1.70 [-2.40, -1.01]
Shang et al., 2020a	9.13	2.8	20	12.34	5.02	20	12.1%	-0.77 [-1.42, -0.13]
Shang et al., 2020b	9.13	2.8	20	15.56	4.32	20	10.8%	-1.73 [-2.47, -0.99]
Shang et al., 2020c	9.13	2.8	20	15.46	5.44	20	11.3%	-1.43 [-2.14, -0.73]
Shang et al., 2020d	9.13	2.8	20	11.5	3.58	20	12.1%	-0.72 [-1.36, -0.08]
Shang et al., 2020e	9.13	2.8	20	14.38	3.99	20	11.2%	-1.49 [-2.20, -0.78]
Shang et al., 2020f	9.13	2.8	20	18.09	6.24	20	10.7%	-1.82 [-2.56, -1.07]
**Total (95% CI)**			**168**			**168**	**100.0%**	**-1.18 [-1.54, -0.82]**

Heterogeneity: $Tau^2 = 0.17$; $Chi^2 = 18.29$, df = 8 (P = 0.02); $I^2 = 56\%$
Test for overall effect: Z = 6.43 (P < 0.00001)

5. Peak pressure under the fourth and fifth metatarsals (kPa)

Study or Subgroup	Control Mean	Control SD	Control Total	HHS Mean	HHS SD	HHS Total	Weight	Std. Mean Difference IV, Random, 95% CI
Guo et al., 2012a	185.3	40.3	13	170.2	66	13	8.4%	0.27 [-0.51, 1.04]
Guo et al., 2012b	185.3	40.3	13	167.7	61	13	8.4%	0.33 [-0.45, 1.10]
Penny et al., 2018	176	45	22	228	56	22	9.1%	-1.01 [-1.64, -0.37]
Shang et al., 2020a	7.8	2.4	20	8.32	2.45	20	9.1%	-0.21 [-0.83, 0.41]
Shang et al., 2020b	7.8	2.4	20	9.08	2.04	20	9.1%	-0.56 [-1.20, 0.07]
Shang et al., 2020c	7.8	2.4	20	8.2	1.58	20	9.1%	-0.19 [-0.81, 0.43]
Shang et al., 2020d	7.8	2.4	20	8.89	2.63	20	9.1%	-0.42 [-1.05, 0.20]
Shang et al., 2020e	7.8	2.4	20	8.75	1.96	20	9.1%	-0.42 [-1.05, 0.20]
Shang et al., 2020f	7.8	2.4	20	9.59	2.56	20	9.1%	-0.71 [-1.35, -0.07]
Snow et al., 1992a	186	61.61	45	358	131.4	45	9.8%	-1.66 [-2.14, -1.18]
Snow et al., 1992b	186	61.61	45	421.37	149.53	45	9.7%	-2.04 [-2.55, -1.53]
**Total (95% CI)**			**258**			**258**	**100.0%**	**-0.63 [-1.09, -0.17]**

Heterogeneity: $Tau^2 = 0.51$; $Chi^2 = 61.05$, df = 10 (P < 0.00001); $I^2 = 84\%$
Test for overall effect: Z = 2.68 (P = 0.007)

Online supplemental appendix 3 Figure 12 Meta-analysis of peak pressure during walking in high-heeled shoes compared with flat shoes or barefoot. *IV* inverse variance, *CI* confidence interval, *HHS* high-heeled shoes.

6. Peak pressure under the midfoot (kPa)

Study or Subgroup	Control Mean	Control SD	Control Total	HHS Mean	HHS SD	HHS Total	Weight	Std. Mean Difference IV, Random, 95% CI
**6.1 Total**								
Penny et al., 2018	133	26	22	87	15	22	7.8%	2.13 [1.38, 2.88]
Shang et al., 2020a	9.16	1.68	20	8.02	1.24	20	8.3%	0.76 [0.11, 1.40]
Shang et al., 2020b	9.16	1.68	20	6.64	1.58	20	8.0%	1.51 [0.80, 2.23]
Shang et al., 2020c	9.16	1.68	20	5.68	0.99	20	7.3%	2.47 [1.63, 3.32]
Shang et al., 2020d	9.16	1.68	20	8.35	2.13	20	8.4%	0.41 [-0.21, 1.04]
Shang et al., 2020e	9.16	1.68	20	6.19	1.19	20	7.7%	2.00 [1.23, 2.77]
Shang et al., 2020f	9.16	1.68	20	6.09	0.86	20	7.5%	2.25 [1.45, 3.06]
**Subtotal (95% CI)**			**142**			**142**	**55.1%**	**1.62 [1.01, 2.23]**
Heterogeneity: Tau^2 = 0.54; Chi^2 = 29.39, df = 6 (P < 0.0001); I^2 = 80%								
Test for overall effect: Z = 5.20 (P < 0.00001)								
**6.2 Lateral**								
Guo et al., 2012a	128.2	41.7	13	77	20.6	13	7.1%	1.51 [0.62, 2.40]
Guo et al., 2012b	128.2	41.7	13	86.4	19.3	13	7.3%	1.25 [0.39, 2.10]
Sun & Gu, 2014	47.54	15.82	18	18.5	8.51	18	7.3%	2.24 [1.38, 3.09]
**Subtotal (95% CI)**			**44**			**44**	**21.6%**	**1.67 [1.08, 2.25]**
Heterogeneity: Tau^2 = 0.07; Chi^2 = 2.77, df = 2 (P = 0.25); I^2 = 28%								
Test for overall effect: Z = 5.56 (P < 0.00001)								
**6.3 Medial**								
Guo et al., 2012a	94.2	48.6	13	54.8	44	13	7.5%	0.82 [0.02, 1.63]
Guo et al., 2012b	94.2	48.6	13	54.3	41.3	13	7.5%	0.86 [0.05, 1.67]
Sun & Gu, 2014	3.34	3.52	18	2.24	3.52	18	8.3%	0.31 [-0.35, 0.96]
**Subtotal (95% CI)**			**44**			**44**	**23.3%**	**0.61 [0.18, 1.04]**
Heterogeneity: Tau^2 = 0.00; Chi^2 = 1.45, df = 2 (P = 0.48); I^2 = 0%								
Test for overall effect: Z = 2.77 (P = 0.006)								
**Total (95% CI)**			**230**			**230**	**100.0%**	**1.40 [0.98, 1.82]**
Heterogeneity: Tau^2 = 0.44; Chi^2 = 47.47, df = 12 (P < 0.00001); I^2 = 75%								
Test for overall effect: Z = 6.53 (P < 0.00001)								
Test for subgroup differences: Chi^2 = 11.25, df = 2 (P = 0.004), I^2 = 82.2%								

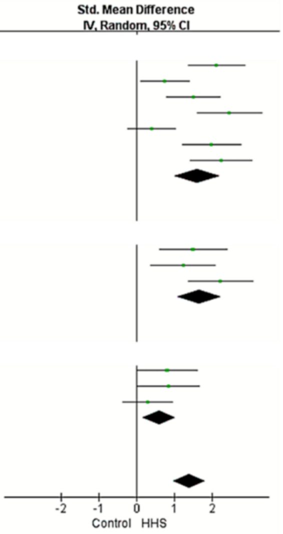

7. Peak pressure under the heel (kPa)

Study or Subgroup	Control Mean	Control SD	Control Total	HHS Mean	HHS SD	HHS Total	Weight	Std. Mean Difference IV, Random, 95% CI
**7.1 Total**								
Guo et al., 2012a	184.5	31.7	13	154.8	32.8	13	5.5%	0.89 [0.08, 1.70]
Guo et al., 2012b	184.5	31.7	13	165.3	32.4	13	5.6%	0.58 [-0.21, 1.37]
Penny et al., 2018	178	49	22	175	32	22	6.3%	0.07 [-0.52, 0.66]
**Subtotal (95% CI)**			**48**			**48**	**17.4%**	**0.45 [-0.04, 0.94]**
Heterogeneity: Tau^2 = 0.05; Chi^2 = 2.79, df = 2 (P = 0.25); I^2 = 28%								
Test for overall effect: Z = 1.78 (P = 0.07)								
**7.2 Lateral**								
Shang et al., 2020a	13.6	4.46	20	11.69	2.98	20	6.1%	0.49 [-0.14, 1.12]
Shang et al., 2020b	13.6	4.46	20	9.47	3.05	20	6.0%	1.06 [0.39, 1.73]
Shang et al., 2020c	13.6	4.46	20	8.76	1.68	20	5.9%	1.41 [0.71, 2.11]
Shang et al., 2020d	13.6	4.46	20	11.78	3.49	20	6.2%	0.45 [-0.18, 1.07]
Shang et al., 2020e	13.6	4.46	20	9.58	1.94	20	6.0%	1.15 [0.47, 1.82]
Shang et al., 2020f	13.6	4.46	20	8.79	1.97	20	5.9%	1.37 [0.67, 2.06]
Sun & Gu, 2014	185.87	31.39	18	191.25	37.69	18	6.1%	-0.15 [-0.81, 0.50]
**Subtotal (95% CI)**			**138**			**138**	**42.2%**	**0.81 [0.39, 1.24]**
Heterogeneity: Tau^2 = 0.22; Chi^2 = 17.30, df = 6 (P = 0.008); I^2 = 65%								
Test for overall effect: Z = 3.74 (P = 0.0002)								
**7.3 Medial**								
Shang et al., 2020a	18.48	3.84	20	17.27	4.3	20	6.2%	0.29 [-0.33, 0.91]
Shang et al., 2020b	18.48	3.84	20	13.39	2.85	20	5.9%	1.48 [0.77, 2.18]
Shang et al., 2020c	18.48	3.84	20	10.43	1.72	20	5.3%	2.65 [1.78, 3.52]
Shang et al., 2020d	18.48	3.84	20	18.59	4.4	20	6.2%	-0.03 [-0.65, 0.59]
Shang et al., 2020e	18.48	3.84	20	12.27	2.6	20	5.7%	1.86 [1.10, 2.61]
Shang et al., 2020f	18.48	3.84	20	10.42	2.3	20	5.4%	2.50 [1.65, 3.34]
Sun & Gu, 2014	222.7	38.16	18	165.2	39.94	18	5.8%	1.44 [0.70, 2.18]
**Subtotal (95% CI)**			**138**			**138**	**40.4%**	**1.42 [0.66, 2.19]**
Heterogeneity: Tau^2 = 0.93; Chi^2 = 46.75, df = 6 (P < 0.00001); I^2 = 87%								
Test for overall effect: Z = 3.64 (P = 0.0003)								
**Total (95% CI)**			**324**			**324**	**100.0%**	**1.00 [0.62, 1.37]**
Heterogeneity: Tau^2 = 0.49; Chi^2 = 78.40, df = 16 (P < 0.00001); I^2 = 80%								
Test for overall effect: Z = 5.23 (P < 0.00001)								
Test for subgroup differences: Chi^2 = 4.51, df = 2 (P = 0.10), I^2 = 55.7%								

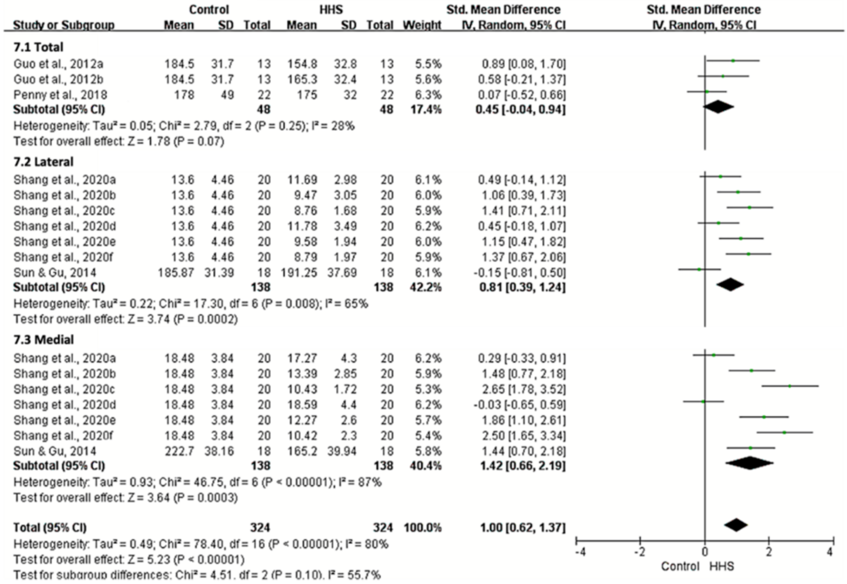

Online supplemental appendix 3 Figure 13 Meta-analysis of peak pressure during walking in high-heeled shoes compared with flat shoes or barefoot. *IV* inverse variance, *CI* confidence interval, *HHS* high-heeled shoes.

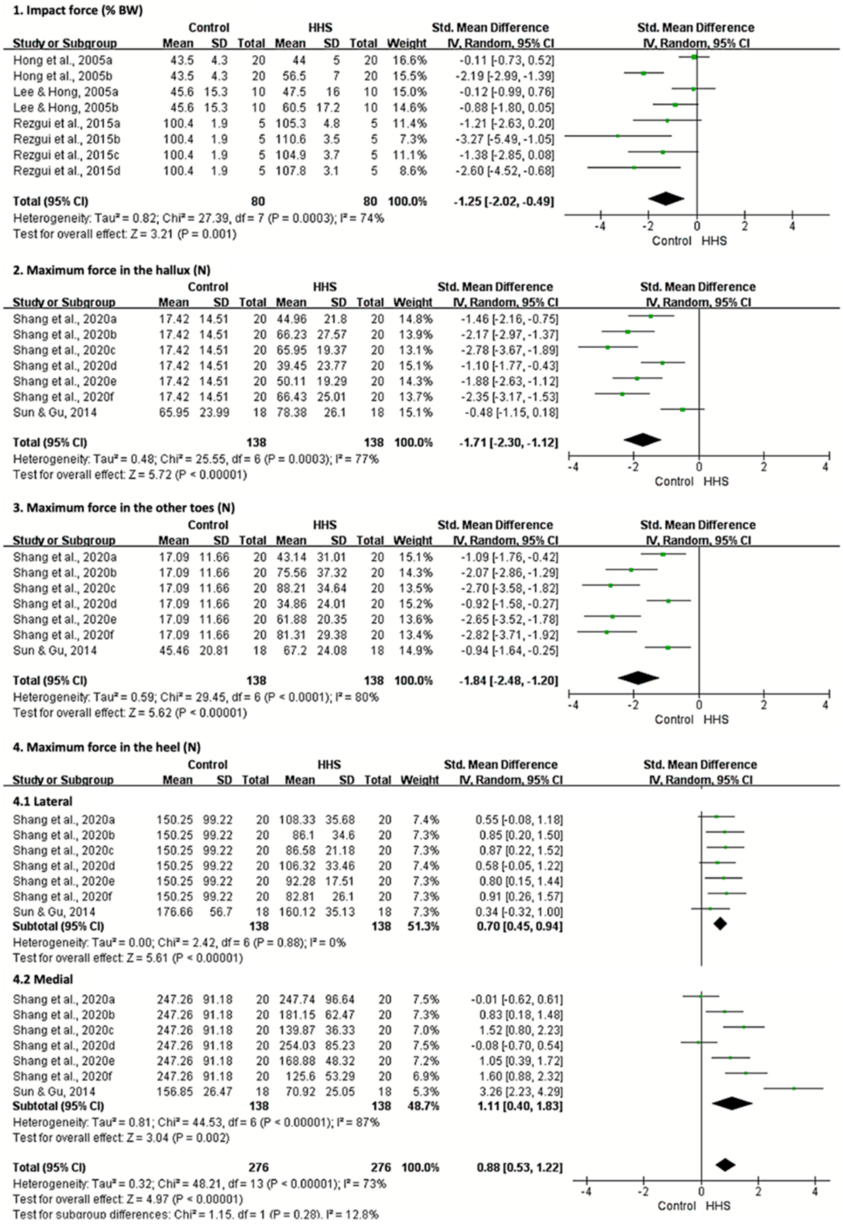

Online supplemental appendix 3 Figure 14 Meta-analysis of impact force during walking in high-heeled shoes compared with flat shoes or barefoot. *IV* inverse variance, *CI* confidence interval, *HHS* high-heeled shoes.

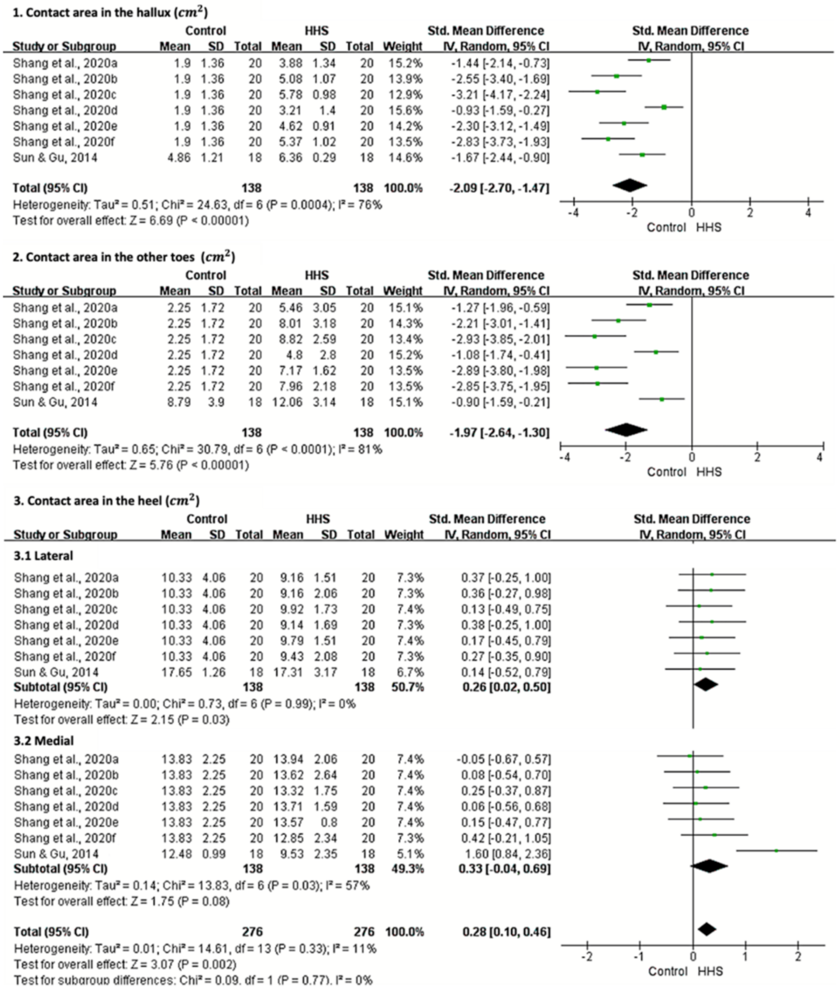

1. Contact area in the hallux (cm^2)

Study or Subgroup	Control Mean	Control SD	Control Total	HHS Mean	HHS SD	HHS Total	Weight	Std. Mean Difference IV, Random, 95% CI
Shang et al., 2020a	1.9	1.36	20	3.88	1.34	20	15.2%	-1.44 [-2.14, -0.73]
Shang et al., 2020b	1.9	1.36	20	5.08	1.07	20	13.9%	-2.55 [-3.40, -1.69]
Shang et al., 2020c	1.9	1.36	20	5.78	0.98	20	12.9%	-3.21 [-4.17, -2.24]
Shang et al., 2020d	1.9	1.36	20	3.21	1.4	20	15.6%	-0.93 [-1.59, -0.27]
Shang et al., 2020e	1.9	1.36	20	4.62	0.91	20	14.2%	-2.30 [-3.12, -1.49]
Shang et al., 2020f	1.9	1.36	20	5.37	1.02	20	13.5%	-2.83 [-3.73, -1.93]
Sun & Gu, 2014	4.86	1.21	18	6.36	0.29	18	14.6%	-1.67 [-2.44, -0.90]
**Total (95% CI)**			**138**			**138**	**100.0%**	**-2.09 [-2.70, -1.47]**

Heterogeneity: Tau² = 0.51; Chi² = 24.63, df = 6 (P = 0.0004); I² = 76%
Test for overall effect: Z = 6.69 (P < 0.00001)

2. Contact area in the other toes (cm^2)

Study or Subgroup	Control Mean	Control SD	Control Total	HHS Mean	HHS SD	HHS Total	Weight	Std. Mean Difference IV, Random, 95% CI
Shang et al., 2020a	2.25	1.72	20	5.46	3.05	20	15.1%	-1.27 [-1.96, -0.59]
Shang et al., 2020b	2.25	1.72	20	8.01	3.18	20	14.3%	-2.21 [-3.01, -1.41]
Shang et al., 2020c	2.25	1.72	20	8.82	2.59	20	13.4%	-2.93 [-3.85, -2.01]
Shang et al., 2020d	2.25	1.72	20	4.8	2.8	20	15.2%	-1.08 [-1.74, -0.41]
Shang et al., 2020e	2.25	1.72	20	7.17	1.62	20	13.5%	-2.89 [-3.80, -1.98]
Shang et al., 2020f	2.25	1.72	20	7.96	2.18	20	13.5%	-2.85 [-3.75, -1.95]
Sun & Gu, 2014	8.79	3.9	18	12.06	3.14	18	15.1%	-0.90 [-1.59, -0.21]
**Total (95% CI)**			**138**			**138**	**100.0%**	**-1.97 [-2.64, -1.30]**

Heterogeneity: Tau² = 0.65; Chi² = 30.79, df = 6 (P < 0.0001); I² = 81%
Test for overall effect: Z = 5.76 (P < 0.00001)

3. Contact area in the heel (cm^2)

Study or Subgroup	Control Mean	Control SD	Control Total	HHS Mean	HHS SD	HHS Total	Weight	Std. Mean Difference IV, Random, 95% CI
**3.1 Lateral**								
Shang et al., 2020a	10.33	4.06	20	9.16	1.51	20	7.3%	0.37 [-0.25, 1.00]
Shang et al., 2020b	10.33	4.06	20	9.16	2.06	20	7.3%	0.36 [-0.27, 0.98]
Shang et al., 2020c	10.33	4.06	20	9.92	1.73	20	7.4%	0.13 [-0.49, 0.75]
Shang et al., 2020d	10.33	4.06	20	9.14	1.69	20	7.3%	0.38 [-0.25, 1.00]
Shang et al., 2020e	10.33	4.06	20	9.79	1.51	20	7.4%	0.17 [-0.45, 0.79]
Shang et al., 2020f	10.33	4.06	20	9.43	2.08	20	7.3%	0.27 [-0.35, 0.90]
Sun & Gu, 2014	17.65	1.26	18	17.31	3.17	18	6.7%	0.14 [-0.52, 0.79]
**Subtotal (95% CI)**			**138**			**138**	**50.7%**	**0.26 [0.02, 0.50]**
Heterogeneity: Tau² = 0.00; Chi² = 0.73, df = 6 (P = 0.99); I² = 0%								
Test for overall effect: Z = 2.15 (P = 0.03)								
**3.2 Medial**								
Shang et al., 2020a	13.83	2.25	20	13.94	2.06	20	7.4%	-0.05 [-0.67, 0.57]
Shang et al., 2020b	13.83	2.25	20	13.62	2.64	20	7.4%	0.08 [-0.54, 0.70]
Shang et al., 2020c	13.83	2.25	20	13.32	1.75	20	7.4%	0.25 [-0.37, 0.87]
Shang et al., 2020d	13.83	2.25	20	13.71	1.59	20	7.4%	0.06 [-0.56, 0.68]
Shang et al., 2020e	13.83	2.25	20	13.57	0.8	20	7.4%	0.15 [-0.47, 0.77]
Shang et al., 2020f	13.83	2.25	20	12.85	2.34	20	7.3%	0.42 [-0.21, 1.05]
Sun & Gu, 2014	12.48	0.99	18	9.53	2.35	18	5.1%	1.60 [0.84, 2.36]
**Subtotal (95% CI)**			**138**			**138**	**49.3%**	**0.33 [-0.04, 0.69]**
Heterogeneity: Tau² = 0.14; Chi² = 13.83, df = 6 (P = 0.03); I² = 57%								
Test for overall effect: Z = 1.75 (P = 0.08)								
**Total (95% CI)**			**276**			**276**	**100.0%**	**0.28 [0.10, 0.46]**

Heterogeneity: Tau² = 0.01; Chi² = 14.61, df = 13 (P = 0.33); I² = 11%
Test for overall effect: Z = 3.07 (P = 0.002)
Test for subgroup differences: Chi² = 0.09, df = 1 (P = 0.77), I² = 0%

Online supplemental appendix 3 Figure 15 Meta-analysis of contact area during walking in high-heeled shoes compared with flat shoes or barefoot. *IV* inverse variance, *CI* confidence interval, *HHS* high-heeled shoes.

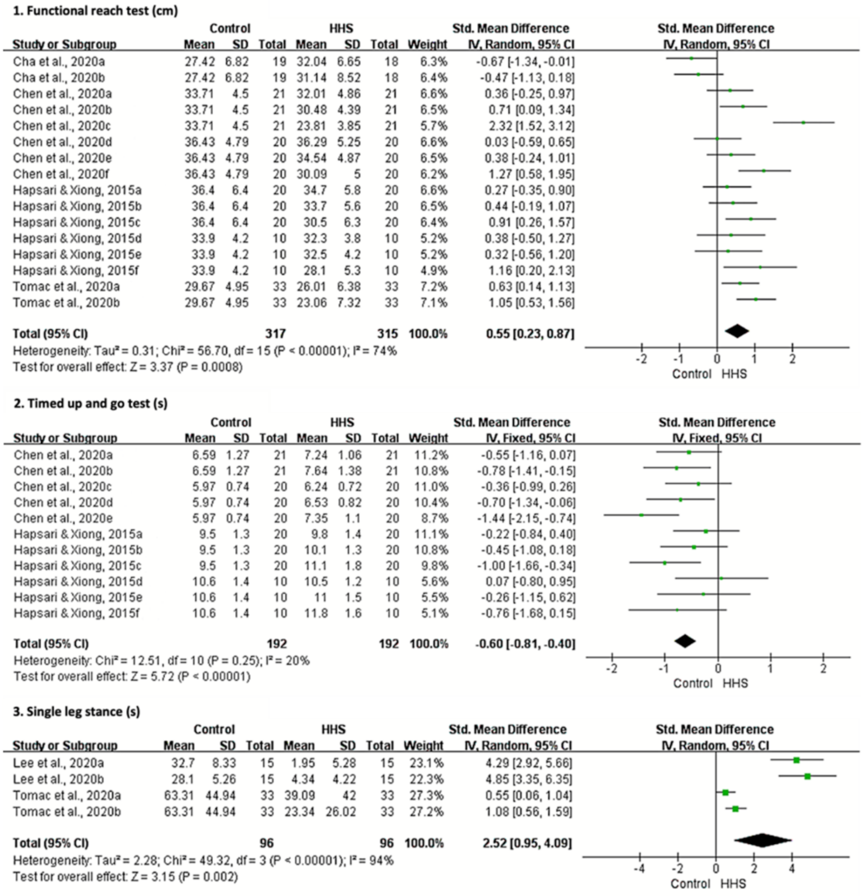

1. Functional reach test (cm)

Study or Subgroup	Control Mean	Control SD	Control Total	HHS Mean	HHS SD	HHS Total	Weight	Std. Mean Difference IV, Random, 95% CI
Cha et al., 2020a	27.42	6.82	19	32.04	6.65	18	6.3%	-0.67 [-1.34, -0.01]
Cha et al., 2020b	27.42	6.82	19	31.14	8.52	18	6.4%	-0.47 [-1.13, 0.18]
Chen et al., 2020a	33.71	4.5	21	32.01	4.86	21	6.6%	0.36 [-0.25, 0.97]
Chen et al., 2020b	33.71	4.5	21	30.48	4.39	21	6.5%	0.71 [0.09, 1.34]
Chen et al., 2020c	33.71	4.5	21	23.81	3.85	21	5.7%	2.32 [1.52, 3.12]
Chen et al., 2020d	36.43	4.79	20	36.29	5.25	20	6.6%	0.03 [-0.59, 0.65]
Chen et al., 2020e	36.43	4.79	20	34.54	4.87	20	6.5%	0.38 [-0.24, 1.01]
Chen et al., 2020f	36.43	4.79	20	30.09	5	20	6.2%	1.27 [0.58, 1.95]
Hapsari & Xiong, 2015a	36.4	6.4	20	34.7	5.8	20	6.6%	0.27 [-0.35, 0.90]
Hapsari & Xiong, 2015b	36.4	6.4	20	33.7	5.6	20	6.5%	0.44 [-0.19, 1.07]
Hapsari & Xiong, 2015c	36.4	6.4	20	30.5	6.3	20	6.4%	0.91 [0.26, 1.57]
Hapsari & Xiong, 2015d	33.9	4.2	10	32.3	3.8	10	5.2%	0.38 [-0.50, 1.27]
Hapsari & Xiong, 2015e	33.9	4.2	10	32.5	4.2	10	5.2%	0.32 [-0.56, 1.20]
Hapsari & Xiong, 2015f	33.9	4.2	10	28.1	5.3	10	4.9%	1.16 [0.20, 2.13]
Tomac et al., 2020a	29.67	4.95	33	26.01	6.38	33	7.2%	0.63 [0.14, 1.13]
Tomac et al., 2020b	29.67	4.95	33	23.06	7.32	33	7.1%	1.05 [0.53, 1.56]
**Total (95% CI)**			**317**			**315**	**100.0%**	**0.55 [0.23, 0.87]**

Heterogeneity: $Tau^2 = 0.31$; $Chi^2 = 56.70$, df = 15 (P < 0.00001); $I^2 = 74\%$
Test for overall effect: Z = 3.37 (P = 0.0008)

2. Timed up and go test (s)

Study or Subgroup	Control Mean	Control SD	Control Total	HHS Mean	HHS SD	HHS Total	Weight	Std. Mean Difference IV, Fixed, 95% CI
Chen et al., 2020a	6.59	1.27	21	7.24	1.06	21	11.2%	-0.55 [-1.16, 0.07]
Chen et al., 2020b	6.59	1.27	21	7.64	1.38	21	10.8%	-0.78 [-1.41, -0.15]
Chen et al., 2020c	5.97	0.74	20	6.24	0.72	20	11.0%	-0.36 [-0.99, 0.26]
Chen et al., 2020d	5.97	0.74	20	6.53	0.82	20	10.4%	-0.70 [-1.34, -0.06]
Chen et al., 2020e	5.97	0.74	20	7.35	1.1	20	8.7%	-1.44 [-2.15, -0.74]
Hapsari & Xiong, 2015a	9.5	1.3	20	9.8	1.4	20	11.1%	-0.22 [-0.84, 0.40]
Hapsari & Xiong, 2015b	9.5	1.3	20	10.1	1.3	20	10.8%	-0.45 [-1.08, 0.18]
Hapsari & Xiong, 2015c	9.5	1.3	20	11.1	1.8	20	9.8%	-1.00 [-1.66, -0.34]
Hapsari & Xiong, 2015d	10.6	1.4	10	10.5	1.2	10	5.6%	0.07 [-0.80, 0.95]
Hapsari & Xiong, 2015e	10.6	1.4	10	11	1.5	10	5.5%	-0.26 [-1.15, 0.62]
Hapsari & Xiong, 2015f	10.6	1.4	10	11.8	1.6	10	5.1%	-0.76 [-1.68, 0.15]
**Total (95% CI)**			**192**			**192**	**100.0%**	**-0.60 [-0.81, -0.40]**

Heterogeneity: $Chi^2 = 12.51$, df = 10 (P = 0.25); $I^2 = 20\%$
Test for overall effect: Z = 5.72 (P < 0.00001)

3. Single leg stance (s)

Study or Subgroup	Control Mean	Control SD	Control Total	HHS Mean	HHS SD	HHS Total	Weight	Std. Mean Difference IV, Random, 95% CI
Lee et al., 2020a	32.7	8.33	15	1.95	5.28	15	23.1%	4.29 [2.92, 5.66]
Lee et al., 2020b	28.1	5.26	15	4.34	4.22	15	22.3%	4.85 [3.35, 6.35]
Tomac et al., 2020a	63.31	44.94	33	39.09	42	33	27.3%	0.55 [0.06, 1.04]
Tomac et al., 2020b	63.31	44.94	33	23.34	26.02	33	27.2%	1.08 [0.56, 1.59]
**Total (95% CI)**			**96**			**96**	**100.0%**	**2.52 [0.95, 4.09]**

Heterogeneity: $Tau^2 = 2.28$; $Chi^2 = 49.32$, df = 3 (P < 0.00001); $I^2 = 94\%$
Test for overall effect: Z = 3.15 (P = 0.002)

Online supplemental appendix 3 Figure 16 Meta-analysis of functional reach test, timed up and go test and single leg stance when wearing high-heeled shoes compared with flat shoes or barefoot. *IV* inverse variance, *CI* confidence interval, *HHS* high-heeled shoes.

1. Limits of stability test
COG movement velocity (°/s)

Study or Subgroup	Control Mean	Control SD	Control Total	HHS Mean	HHS SD	HHS Total	Weight	Std. Mean Difference IV, Random, 95% CI
Chen et al., 2020a	4.86	1.79	21	4.46	1.44	21	12.8%	0.24 [-0.37, 0.85]
Chen et al., 2020b	4.86	1.79	21	4.34	1.39	21	12.8%	0.32 [-0.29, 0.93]
Chen et al., 2020c	4.86	1.79	21	3.58	1.16	21	12.4%	0.83 [0.20, 1.47]
Hapsari & Xiong, 2015a	82.33	5.62	20	82	6.83	20	12.6%	0.05 [-0.57, 0.67]
Hapsari & Xiong, 2015b	82.33	5.62	20	77.67	8.48	20	12.3%	0.63 [-0.00, 1.27]
Hapsari & Xiong, 2015c	82.33	5.62	20	68.62	9.74	20	11.0%	1.69 [0.96, 2.42]
Hapsari & Xiong, 2015d	84.45	3.98	10	84.95	3.2	10	9.2%	-0.13 [-1.01, 0.75]
Hapsari & Xiong, 2015e	84.45	3.98	10	80.2	5.65	10	8.7%	0.83 [-0.09, 1.76]
Hapsari & Xiong, 2015f	84.45	3.98	10	77.45	6.53	10	8.2%	1.24 [0.26, 2.22]
**Total (95% CI)**			**153**			**153**	**100.0%**	**0.61 [0.24, 0.97]**

Heterogeneity: $Tau^2 = 0.17$; $Chi^2 = 18.73$, $df = 8$ ($P = 0.02$); $I^2 = 57\%$
Test for overall effect: $Z = 3.27$ ($P = 0.001$)

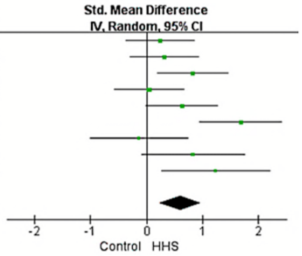

2. Limits of stability test
Directional control (%)

Study or Subgroup	Control Mean	Control SD	Control Total	HHS Mean	HHS SD	HHS Total	Weight	Std. Mean Difference IV, Fixed, 95% CI
Chen et al., 2020a	5.92	1.5	21	6.06	1.5	21	10.4%	-0.09 [-0.70, 0.51]
Chen et al., 2020b	5.92	1.5	21	5.63	1.5	21	10.3%	0.19 [-0.42, 0.80]
Chen et al., 2020c	5.92	1.5	21	5.39	1.6	21	10.2%	0.34 [-0.27, 0.94]
Chen et al., 2020d	5.74	1.1	20	5.76	1.6	20	9.9%	-0.01 [-0.63, 0.61]
Chen et al., 2020e	5.74	1.1	20	5.55	1.3	20	9.8%	0.15 [-0.47, 0.78]
Chen et al., 2020f	5.74	1.1	20	5.34	1.2	20	9.7%	0.34 [-0.28, 0.97]
Hapsari & Xiong, 2015a	80.7	6.5	20	78.7	9.7	20	9.8%	0.24 [-0.38, 0.86]
Hapsari & Xiong, 2015b	80.7	6.5	20	73.05	12.3	20	9.1%	0.76 [0.12, 1.41]
Hapsari & Xiong, 2015c	80.7	6.5	20	65.3	12.5	20	7.5%	1.52 [0.80, 2.23]
Hapsari & Xiong, 2015d	81.5	6.1	10	81.3	7.1	10	4.9%	0.03 [-0.85, 0.91]
Hapsari & Xiong, 2015e	81.5	6.1	10	76.1	7.8	10	4.5%	0.74 [-0.17, 1.65]
Hapsari & Xiong, 2015f	81.5	6.1	10	68.1	11.5	10	3.8%	1.39 [0.39, 2.39]
**Total (95% CI)**			**213**			**213**	**100.0%**	**0.39 [0.19, 0.58]**

Heterogeneity: $Chi^2 = 21.29$, $df = 11$ ($P = 0.03$); $I^2 = 48\%$
Test for overall effect: $Z = 3.88$ ($P = 0.0001$)

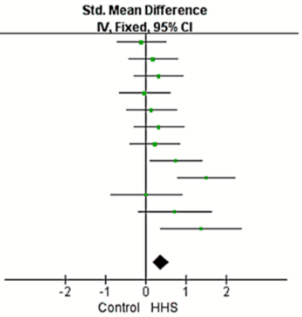

Online supplemental appendix 3 Figure 17 Meta-analysis of limits of stability test when wearing high-heeled shoes compared with flat shoes or barefoot. *COG* centre of gravity, *IV* inverse variance, *CI* confidence interval, *HHS* high-heeled shoes.

1. Mean COPx (mm)

	Control			HHS				Std. Mean Difference
Study or Subgroup	Mean	SD	Total	Mean	SD	Total	Weight	IV, Random, 95% CI
Truszcynska et al., 2016a	1.5	1	71	1.5	0.9	71	11.8%	0.00 [-0.33, 0.33]
Truszcynska et al., 2016b	1.5	1	71	2.2	1.1	71	11.7%	-0.66 [-1.00, -0.32]
Truszcynska et al., 2016c	1.7	1.3	71	1.9	1.5	71	11.8%	-0.14 [-0.47, 0.19]
Truszcynska et al., 2016d	1.7	1.3	71	3.3	1.8	71	11.7%	-1.01 [-1.36, -0.66]
Wan et al., 2018a	44	2.73	12	42.51	2.58	12	9.1%	0.54 [-0.28, 1.36]
Wan et al., 2018b	44	2.73	12	40.43	2.52	12	8.7%	1.31 [0.41, 2.21]
Wan et al., 2018c	44	2.73	12	38.87	3.16	12	8.3%	1.68 [0.72, 2.63]
Wan et al., 2018d	42.88	3.73	12	42.09	2.73	12	9.2%	0.23 [-0.57, 1.04]
Wan et al., 2018e	42.88	3.73	12	40.18	2.35	12	9.0%	0.84 [-0.00, 1.68]
Wan et al., 2018f	42.88	3.73	12	38.81	2.56	12	8.7%	1.23 [0.34, 2.11]
**Total (95% CI)**			**356**			**356**	**100.0%**	**0.29 [-0.18, 0.77]**

Heterogeneity: $Tau^2 = 0.48$; $Chi^2 = 75.33$, $df = 9$ ($P < 0.00001$); $I^2 = 88\%$

Test for overall effect: $Z = 1.20$ ($P = 0.23$)

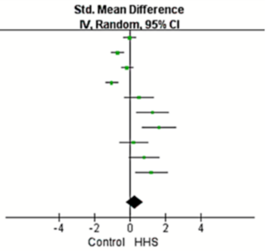

2. Mean COPy (mm)

	Control			HHS				Std. Mean Difference
Study or Subgroup	Mean	SD	Total	Mean	SD	Total	Weight	IV, Random, 95% CI
Truszcynska et al., 2016a	0.5	0.5	71	0.5	0.6	71	12.8%	0.00 [-0.33, 0.33]
Truszcynska et al., 2016b	0.5	0.5	71	0.7	1	71	12.7%	-0.25 [-0.58, 0.08]
Truszcynska et al., 2016c	0.5	0.7	71	0.5	0.5	71	12.8%	0.00 [-0.33, 0.33]
Truszcynska et al., 2016d	0.5	0.7	71	0.8	0.6	71	12.7%	-0.46 [-0.79, -0.12]
Wan et al., 2018a	81.14	18.34	12	93.67	17.14	12	9.2%	-0.68 [-1.51, 0.15]
Wan et al., 2018b	81.14	18.34	12	107.31	10.04	12	8.3%	-1.71 [-2.67, -0.75]
Wan et al., 2018c	81.14	18.34	12	118.04	13.15	12	7.7%	-2.23 [-3.29, -1.18]
Wan et al., 2018d	88.64	13.22	12	97.07	12.62	12	9.3%	-0.63 [-1.45, 0.19]
Wan et al., 2018e	88.64	13.22	12	111.79	9.21	12	8.0%	-1.96 [-2.97, -0.96]
Wan et al., 2018f	88.64	13.22	12	122.41	7.18	12	6.6%	-3.07 [-4.31, -1.82]
**Total (95% CI)**			**356**			**356**	**100.0%**	**-0.88 [-1.32, -0.44]**

Heterogeneity: $Tau^2 = 0.37$; $Chi^2 = 58.59$, $df = 9$ ($P < 0.00001$); $I^2 = 85\%$

Test for overall effect: $Z = 3.93$ ($P < 0.0001$)

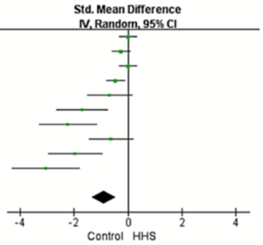

3. Mean COPx velocity (mm/s)

	Control			HHS				Std. Mean Difference
Study or Subgroup	Mean	SD	Total	Mean	SD	Total	Weight	IV, Random, 95% CI
Truszcynska et al., 2016a	3.6	0.8	71	4.4	1.3	71	11.0%	-0.74 [-1.08, -0.40]
Truszcynska et al., 2016b	3.6	0.8	71	7.4	2.7	71	10.9%	-1.90 [-2.30, -1.50]
Truszcynska et al., 2016c	4.3	1.5	71	5.6	2.1	71	11.0%	-0.71 [-1.05, -0.37]
Truszcynska et al., 2016d	4.3	1.5	71	12.4	7	71	10.9%	-1.59 [-1.97, -1.21]
Wan et al., 2018a	6.71	2.23	12	5.61	1.15	12	9.3%	0.60 [-0.22, 1.42]
Wan et al., 2018b	6.71	2.23	12	5.4	1.22	12	9.3%	0.70 [-0.13, 1.53]
Wan et al., 2018c	6.71	2.23	12	6	0.81	12	9.4%	0.41 [-0.40, 1.22]
Wan et al., 2018d	5.28	1.29	12	4.84	1.24	12	9.4%	0.34 [-0.47, 1.14]
Wan et al., 2018e	5.28	1.29	12	4.49	0.97	12	9.3%	0.67 [-0.16, 1.49]
Wan et al., 2018f	5.28	1.29	12	4.94	0.88	12	9.4%	0.30 [-0.51, 1.10]
**Total (95% CI)**			**356**			**356**	**100.0%**	**-0.26 [-0.84, 0.32]**

Heterogeneity: $Tau^2 = 0.76$; $Chi^2 = 105.60$, $df = 9$ ($P < 0.00001$); $I^2 = 91\%$

Test for overall effect: $Z = 0.88$ ($P = 0.38$)

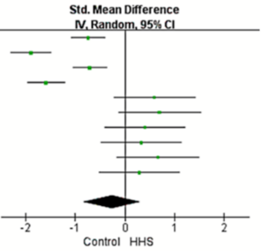

4. Mean COPy velocity (mm/s)

	Control			HHS				Std. Mean Difference
Study or Subgroup	Mean	SD	Total	Mean	SD	Total	Weight	IV, Random, 95% CI
Truszcynska et al., 2016a	2.7	0.6	71	2.8	1	71	12.5%	-0.12 [-0.45, 0.21]
Truszcynska et al., 2016b	2.7	0.6	71	3.3	1.3	71	12.4%	-0.59 [-0.93, -0.25]
Truszcynska et al., 2016c	2.7	0.9	71	2.9	0.9	71	12.4%	-0.22 [-0.55, 0.11]
Truszcynska et al., 2016d	2.7	0.9	71	3.8	1.8	71	12.4%	-0.77 [-1.11, -0.43]
Wan et al., 2018a	30.88	10.29	12	23.97	5.35	12	8.4%	0.81 [-0.03, 1.65]
Wan et al., 2018b	30.88	10.29	12	21.71	4.92	12	8.2%	1.10 [0.23, 1.97]
Wan et al., 2018c	30.88	10.29	12	26.56	4.5	12	8.6%	0.53 [-0.29, 1.34]
Wan et al., 2018d	22.37	6.54	12	18.45	4.96	12	8.5%	0.65 [-0.17, 1.48]
Wan et al., 2018e	22.37	6.54	12	16.06	3.68	12	8.1%	1.15 [0.27, 2.02]
Wan et al., 2018f	22.37	6.54	12	18.66	4.07	12	8.5%	0.66 [-0.17, 1.48]
**Total (95% CI)**			**356**			**356**	**100.0%**	**0.20 [-0.20, 0.59]**

Heterogeneity: $Tau^2 = 0.29$; $Chi^2 = 50.58$, $df = 9$ ($P < 0.00001$); $I^2 = 82\%$

Test for overall effect: $Z = 0.99$ ($P = 0.32$)

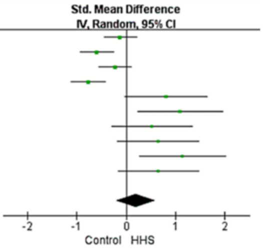

5. Medial-lateral COP during walking (cm)

	Control			HHS				Std. Mean Difference
Study or Subgroup	Mean	SD	Total	Mean	SD	Total	Weight	IV, Fixed, 95% CI
Hyun & Ryew, 2018	3.07	1.39	9	2.28	0.49	9	14.8%	0.72 [-0.24, 1.68]
Yu et al., 2021a	0.27	0.33	12	0.31	0.04	12	21.3%	-0.16 [-0.97, 0.64]
Yu et al., 2021b	0.27	0.33	12	0.3	0.04	12	21.3%	-0.12 [-0.92, 0.68]
Yu et al., 2021c	0.27	0.33	12	0.31	0.12	12	21.3%	-0.16 [-0.96, 0.65]
Yu et al., 2021d	0.27	0.33	12	0.32	0.02	12	21.3%	-0.21 [-1.01, 0.60]
**Total (95% CI)**			**57**			**57**	**100.0%**	**-0.03 [-0.40, 0.34]**

Heterogeneity: $Chi^2 = 2.79$, $df = 4$ ($P = 0.59$); $I^2 = 0\%$

Test for overall effect: $Z = 0.17$ ($P = 0.87$)

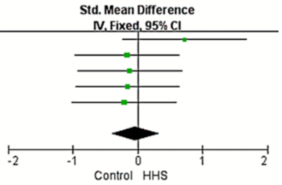

6. Anterior-posterior COP during walking (cm)

	Control			HHS				Std. Mean Difference
Study or Subgroup	Mean	SD	Total	Mean	SD	Total	Weight	IV, Fixed, 95% CI
Hyun & Ryew, 2018	19.83	1.23	9	18.42	2.31	9	14.8%	0.73 [-0.24, 1.69]
Yu et al., 2021a	0.24	0.35	12	0.23	0.06	12	21.3%	0.04 [-0.76, 0.84]
Yu et al., 2021b	0.24	0.35	12	0.19	0.02	12	21.2%	0.19 [-0.61, 1.00]
Yu et al., 2021c	0.24	0.35	12	0.22	0.01	12	21.3%	0.08 [-0.72, 0.88]
Yu et al., 2021d	0.24	0.35	12	0.23	0.03	12	21.3%	0.04 [-0.76, 0.84]
**Total (95% CI)**			**57**			**57**	**100.0%**	**0.18 [-0.19, 0.55]**

Heterogeneity: $Chi^2 = 1.54$, $df = 4$ ($P = 0.82$); $I^2 = 0\%$

Test for overall effect: $Z = 0.96$ ($P = 0.34$)

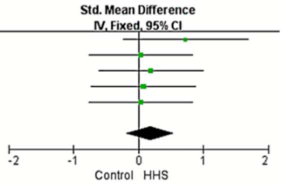

Online supplemental appendix 3 Figure 18 Meta-analysis of centre of pressure when wearing high-heeled shoes compared with flat shoes or barefoot. *COP* centre of pressure, *IV* inverse variance, *CI* confidence interval, *HHS* high-heeled shoes.
