# Supplementary material for: Effects of high-heeled shoes on lower extremity biomechanics and balance in females: a systematic review and meta-analysis
Source: BMC Public Health. 2023 Apr 20;23:726. doi: 10.1186/s12889-023-15641-8 (PMC10120101; doi:10.1186/s12889-023-15641-8)
Supplement: Supplementary file 6 — Additional file 6. [file 12889_2023_15641_MOESM6_ESM.pdf]

Additional file 6

The funnel plots of spatiotemporal, kinematics, kinetics, plantar pressure and balance outcomes

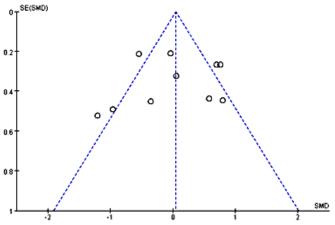

Stance time

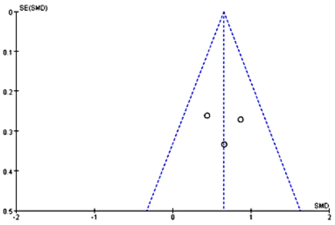

Swing time

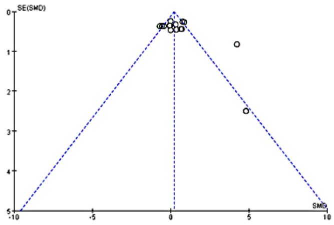

Stride time

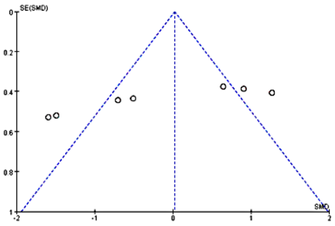

Single leg support time

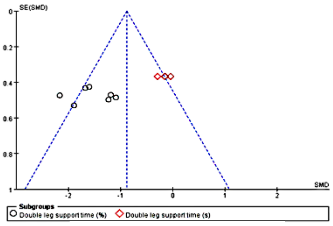

Double leg support time

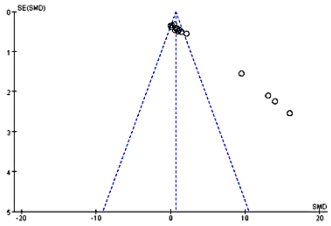

Step length

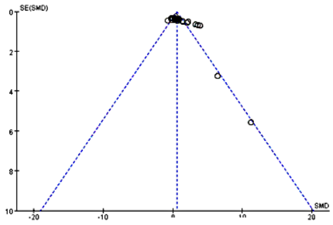

Stride length

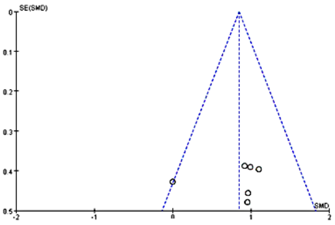

Step width

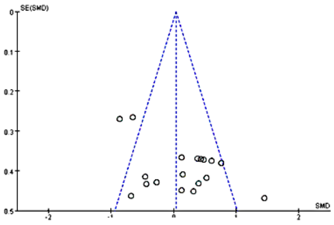

Step frequency

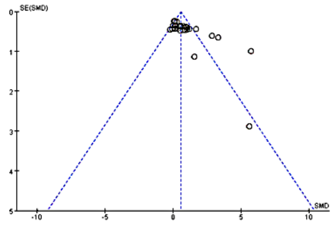

Walking velocity

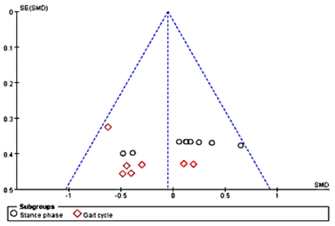

Hip flexion peak angle

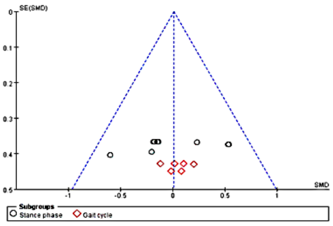

Hip extension peak angle

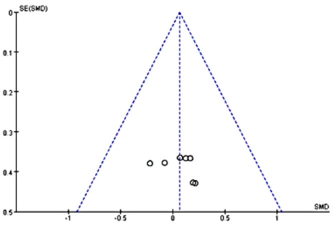

Hip flexion angle at foot strike

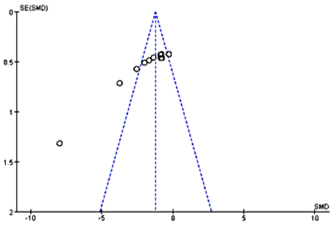

Hip ROM during the gait cycle

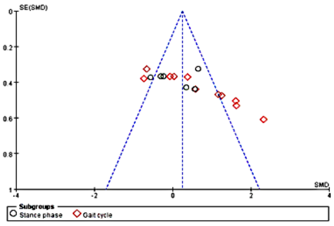

Knee flexion peak angle

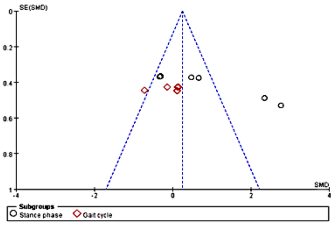

Knee extension peak angle

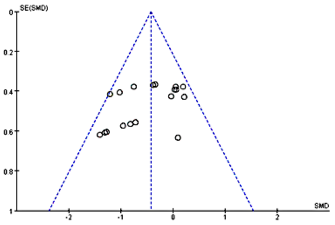

Knee flexion angle at foot strike

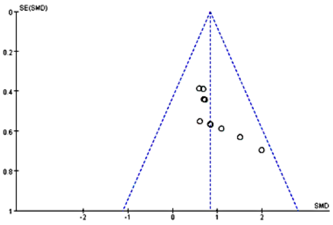

Knee flexion angle at toe-off

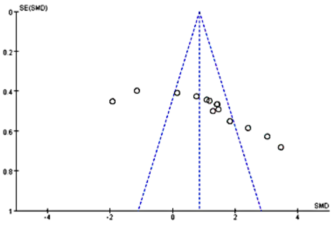

Knee ROM during the gait cycle

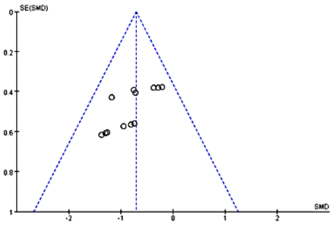

Knee flexion angle during the mid-stance

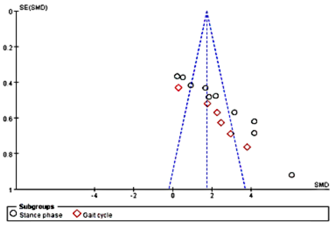

Ankle plantarflexion peak angle

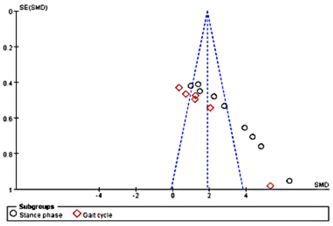

Ankle dorsiflexion peak angle

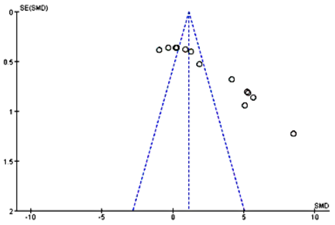

Ankle angle at foot strike

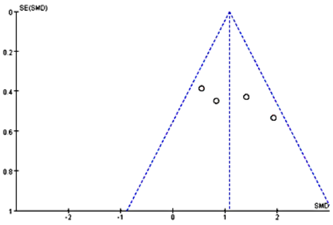

Ankle angle at toe-off

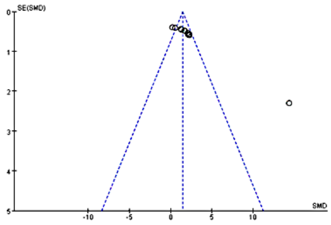

Ankle ROM during the gait cycle

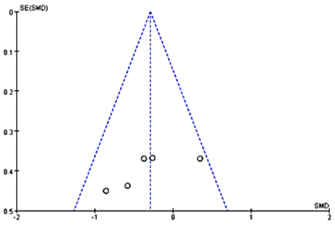

Rearfoot angle at foot strike

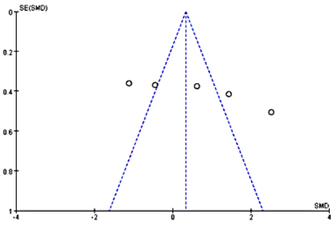

Rearfoot inversion peak angle during the stance phase

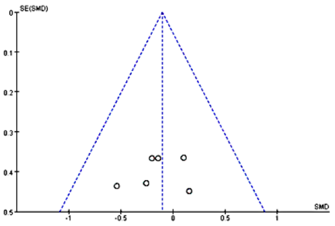

Hip flexion peak moment during the gait cycle

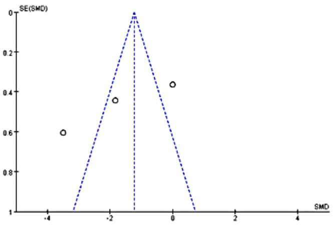

Hip extension peak moment during the gait cycle

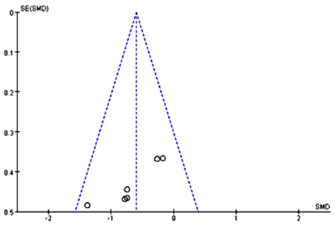

Knee flexion peak moment during the gait cycle

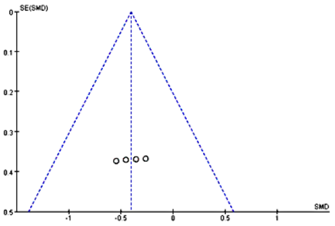

Knee extension peak moment during the gait cycle

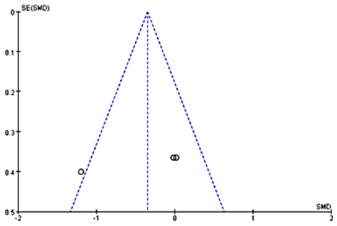

Knee adduction peak moment during the gait cycle

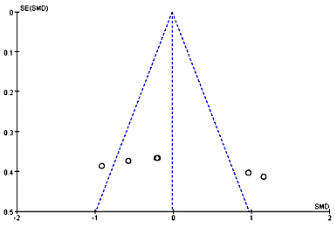

Knee abduction peak moment during the gait cycle

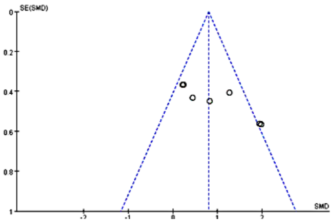

Ankle plantarflexion peak moment during the gait cycle

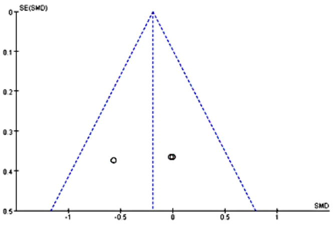

Ankle dorsiflexion peak moment during the gait cycle

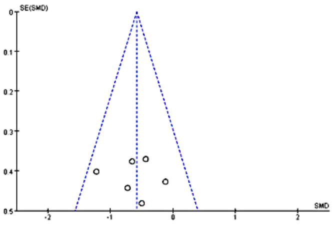

First peak vertical GRF

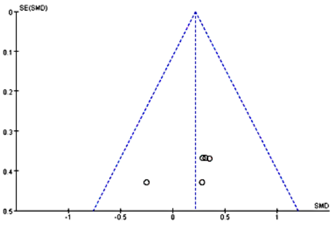

% time to first peak vertical GRF

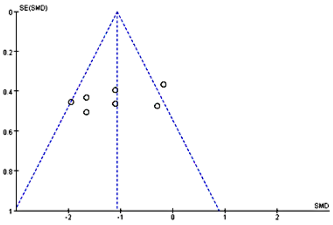

Second peak vertical GRF

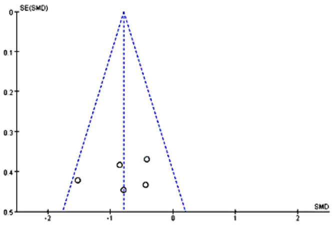

% time to second peak vertical GRF

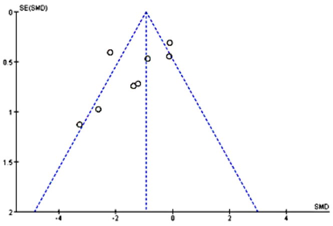

Impact force

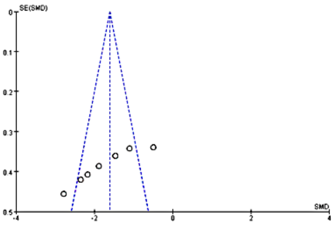

Maximum force in the hallux

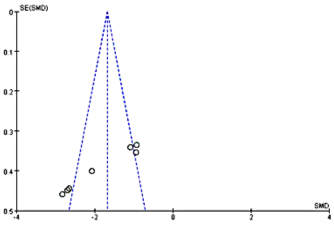

Maximum force in the other toes

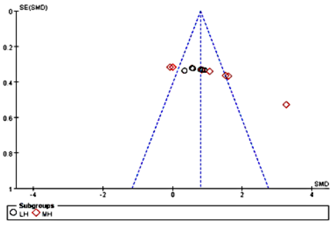

Maximum force in the heel

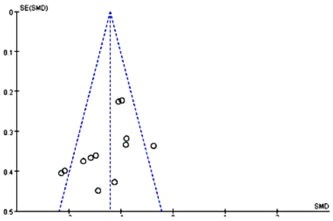

Peak pressure under the hallux

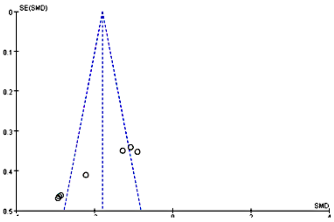

Peak pressure under other toes

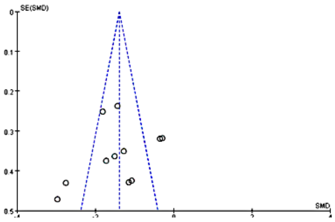

Peak pressure under the first metatarsals

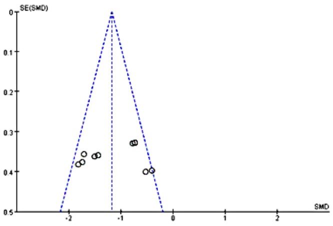

Peak pressure under the second and third metatarsals

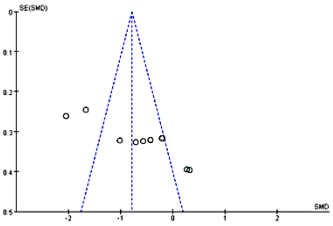

Peak pressure under the fourth and fifth metatarsals

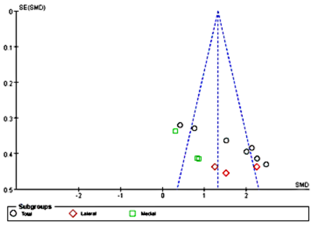

Peak pressure under the midfoot

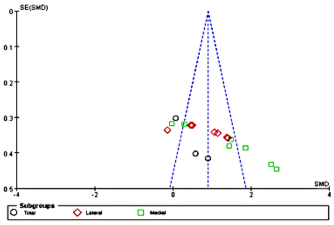

Peak pressure under the heel

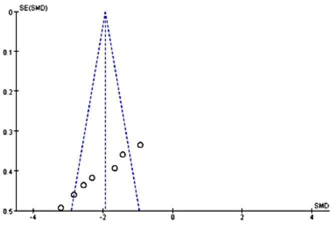

Contact area in the hallux

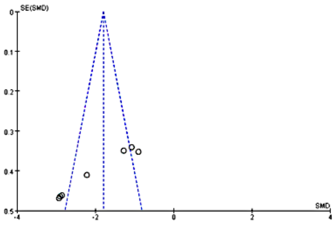

Contact area in other toes

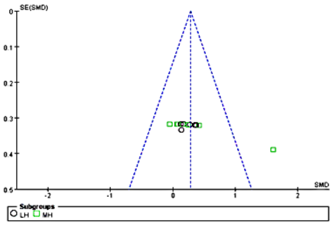

Contact area in the heel

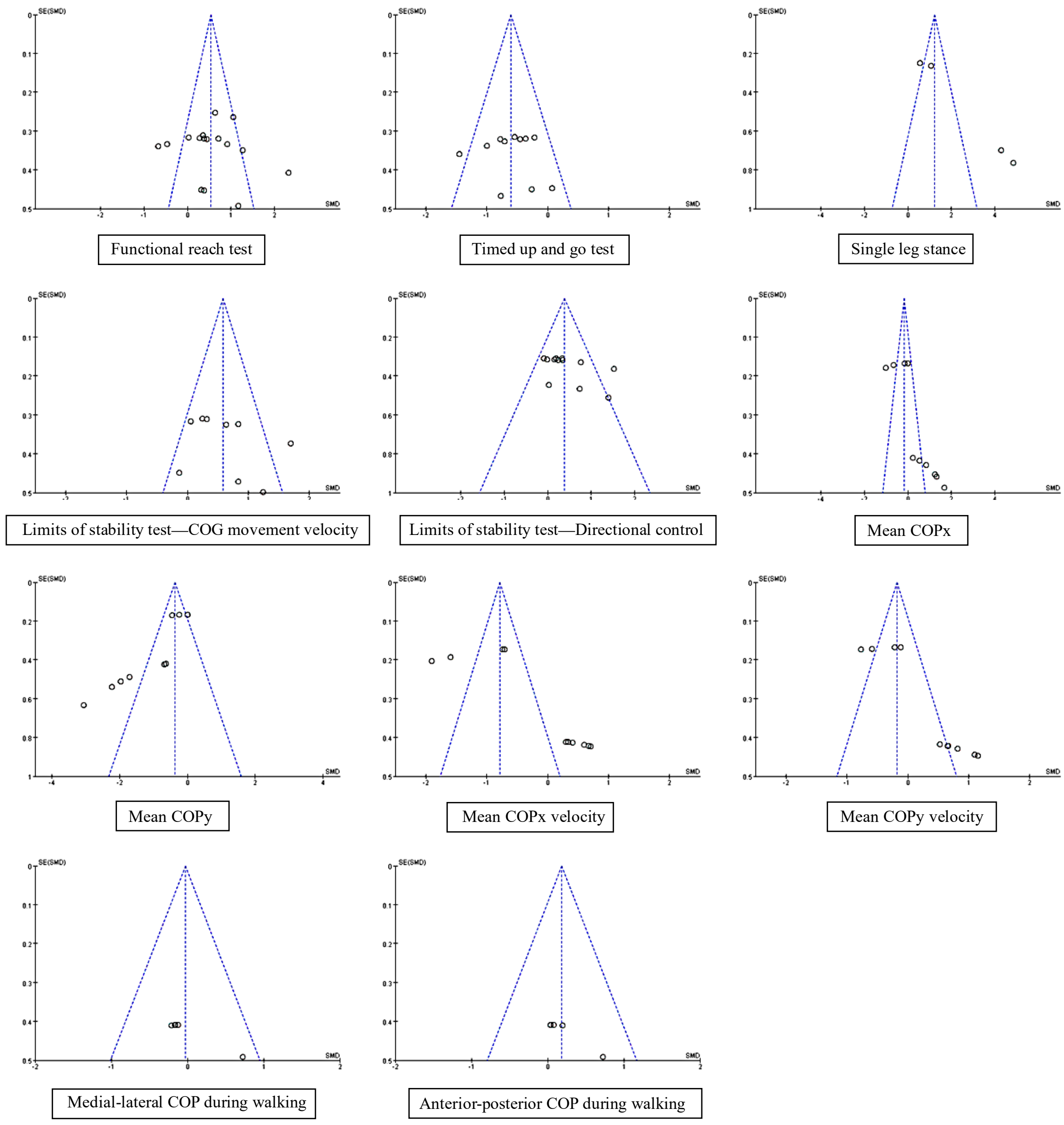

SMD standardized mean differences, *SE* effect sizes.
